# Alternative enzymatic pathways to penicillin antibiotics

**DOI:** 10.1038/s41467-026-72466-w

**Published:** 2026-04-30

**Authors:** Puja Saha, Guangcai Xu, Deepanjan Panda, Duncan Smith, Wei Li Thong, Luke Ward, Luis Bering, Sebastian Cuesta-Hoyos, Sarah A. Shepherd, Jason Micklefield

**Affiliations:** 1 Department of Chemistry, Imperial College London, Molecular Sciences Research Hub, London, UK; 2 Department of Chemistry and Manchester Institute of Biotechnology, The University of Manchester, Manchester, UK

**Keywords:** Ligases, Biocatalysis, Biosynthesis

## Abstract

The discovery of penicillin, more than a century ago, has been one of the most significant advances in medicine. Despite the growing threat of antimicrobial resistance, which has rendered many other antibiotics ineffective, penicillin derivatives remain among the most widely prescribed antibiotics. Penicillin is biosynthesised by a large nonribosomal peptide synthetase (NRPS) enzyme, which assembles a tripeptide precursor ACV. This intermediate is subsequently cyclised by isopenicillin N synthase (IPNS) to form penicillin. ACV is similar in structure to glutathione, a ubiquitous, tripeptide antioxidant essential for aerobic life forms. Unlike ACV, glutathione is assembled using simpler ligase enzymes rather than complex NRPS machinery. In this paper, we describe an alternative pathway to penicillins that uses stand-alone ligase and epimerase enzymes to generate peptide precursors, which can be transformed to penicillin derivatives using an engineered IPNS enzyme. Unlike the native NRPS assembly line, the ligase pathway provides direct access to therapeutically relevant penicillin G, penicillin V and ampicillin, which are currently produced by semi-synthesis.

## Abstract (web summary)

Penicillin derivatives remain among the most widely prescribed antibiotics. In this study, the authors report an alternative pathway to penicillins that uses standalone ligase and epimerase enzymes to generate peptide precursors, which can be transformed into penicillin derivatives using an engineered isopenicillin N synthase enzyme, providing direct access to therapeutically relevant penicillin G, penicillin V and ampicillin, which are currently produced by semisynthesis.

## Introduction

**Fig. 1:**
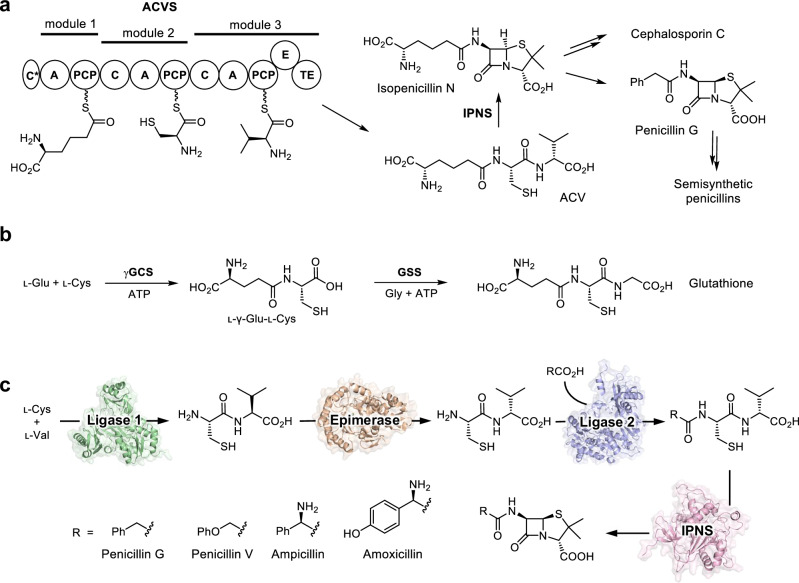
Alternative ligase route to penicillin. **a** Biosynthesis of β-lactam antibiotics proceeds via NRPS assembly of the ACV tripeptide. **b** Biosynthesis of glutathione with ATP-grasp ligase enzymes (γGCS and GSS). **c** Alternative pathway to penicillin derivatives (this work).

The discovery of penicillin was one of the most significant breakthroughs of the last century, ushering in the era of antibiotics. Even 80 years after its discovery, penicillin and other β-lactam antibiotics remain amongst the most widely used classes of antibiotic treatments^1^. The use of β-lactam antibiotics was hampered by the emergence of resistance in the form of β-lactamase enzymes^2–4^. However, the introduction of β-lactamase inhibitors, such as clavulanic acid, has led to combination therapies that remain very effective^1,5,6^. Penicillin and related cephalosporins are biosynthesised by various bacterial and fungal species. An ʟ-δ-(α-aminoadipoyl)-ʟ-cysteinyl-ᴅ-valine synthetase (ACVS) from the non-ribosomal peptide synthetase (NRPS) family of enzymes assembles the ACV tripeptide^7–10^. Isopenicillin N synthase (IPNS) catalyses the cyclisation of ACV, generating isopenicillin N^11–13^, at which point the pathway diverges to produce penicillin G and cephalosporin C (Fig. 1a)^7,8^.

The most effective penicillin derivatives in use today, including amoxicillin, are currently produced by a semi-synthetic route. First, penicillin G is produced by fermentation and then a biotransformation with penicillin G acylase is used to remove the phenylacetate side chain. The resulting 6-aminopenicillanic acid (6-APA) is isolated and subsequent chemical/enzymatic steps are used to produce amoxicillin, ampicillin and other penicillin derivatives^14–16^. Although this route is well established, supply chain issues and recent increases in respiratory infections have led to major shortages of amoxicillin^17^. Alternative routes to penicillin derivatives would be useful. One possibility would be to engineer ACV synthetase, replacing the first NRPS module that activates and incorporates α-aminoadipic acid, with modules that introduce alternative acyl groups. For instance, an NRPS that incorporates phenylglycine (Pg) or 4-hydroxyphenylglycine (Hpg) may provide tripeptides which upon cyclisation by IPNS, would generate ampicillin and amoxicillin, respectively. Although progress has been made in engineering NRPS assembly lines, this has involved exchanging NRPS components from closely related NRPS systems, leading to conservative structural/peptide sequence changes, in typically low yields^18–21^. ACV synthetase has unusual features, including condensation via the α-aminoadipate side chain carboxyl group; this, combined with the necessity to epimerise Pg or Hpg to the ᴅ-configuration before condensation with ʟ-Cys, makes the engineering of ACVS challenging. Analysis of NRPS protein sequences to known or predicted NRPS products reveals very few evolutionarily related and potentially compatible NRPS components that might be fused together, using existing NRPS engineering strategies, to deliver the required tripeptides^18–21^.

Given the challenges associated with NRPS engineering, we considered alternative enzymatic routes to penicillin derivatives. We were inspired by the structural similarity of ACV to glutathione, the ubiquitous tripeptide antioxidant, which is essential for aerobic life. Despite its structural similarity to ACV, glutathione is assembled by two ligase enzymes (Fig. 1b). First, glutamate–cysteine ligase (GCL, alternatively known as γ-glutamyl cysteine synthetase, γ-GCS) couples ʟ-Glu and ʟ-Cys. The resulting dipeptide ʟ-γ-Glu-ʟ-Cys is then ligated to Gly by glutathione synthetase (GSS) to form the glutathione^22,23^. The two ligases (GCL and GSS) both possess the ATP-grasp fold utilising ATP to activate carboxylic acids, forming acyl-phosphate intermediates which are attacked by amine nucleophiles to generate peptide bonds^24,25^. Unlike NRPS, which are very large and complex enzyme systems, comprising multiple domains (ACVS has 10 domains, ca. 3770 amino acid residues), the ATP-grasp ligases are relatively simple stand-alone enzymes (*E. coli* GCL and GSS have 518 and 316 amino acid residues, respectively). In addition to glutathione, other essential peptides, including the peptidoglycan pentapeptide component of bacterial cell walls, are assembled by simple ligase enzymes^26^.

In this work, we describe an alternative route to penicillins, repurposing an ATP-grasp enzyme to ligate ʟ-Cys and ʟ-Val along with an epimerase to generate ʟ-Cys-ᴅ-Val. Engineered acyl-CoA ligase enzymes are then used to deliver a number of *N*-acyl-ʟ-Cys-ᴅ-Val peptides that are cyclised by an IPNS variant to generate penicillin derivatives.

## Results

### Cysteine-valine ligation

**Fig. 2:**
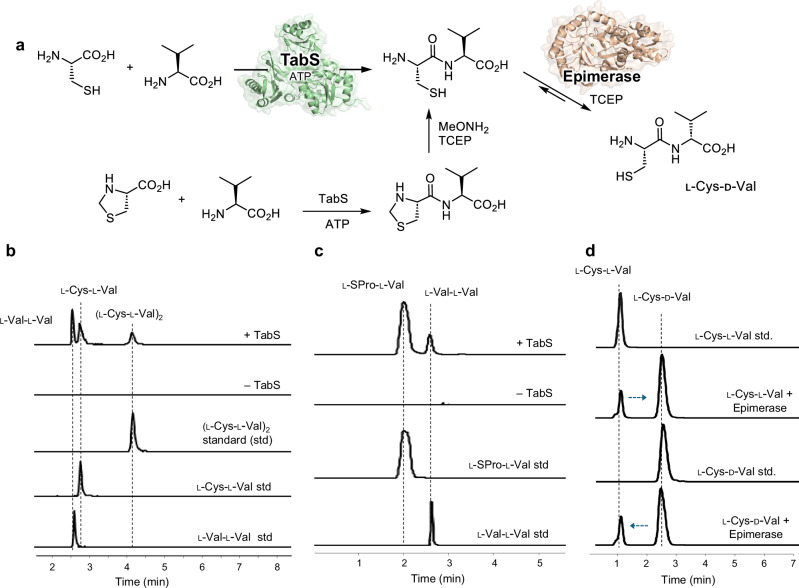
l-Cys-d-Val derived by TabS and *T. maritima* epimerase. **a** Ligase and epimerase mediated production of ʟ-Cys-d-Val dipeptide from ʟ-Cys or ʟ-SPro and ʟ-Val. **b** TabS assays with ʟ-Cys and ʟ-Val, showing the formation of ʟ-Cys-ʟ-Val dipeptide and its dimeric disulphide form (ʟ-Cys-ʟ-Val)_2_ along with the ʟ-Val-ʟ-Val side product. **c** TabS coupling of ʟ-SPro and ʟ-Val producing ʟ-SPro-ʟ-Val, which upon treatment with methoxyamine hydrochloride (MeONH_2_.HCl) generates ʟ-Cys-ʟ-Val. **d** Epimerisation assay showing the epimerisation of ʟ-Cys-ʟ-Val and ʟ-Cys-d-Val using the *T. maritima* epimerase. Source data for fig. b-d are provided as a Source Data file.

We envisaged a route to the target penicillin derivatives, using a stand-alone ligase to generate a ʟ-Cys-ᴅ-Val dipeptide followed by acylation of the N-terminal Cys (Fig. 1c). We reasoned this route could exploit the Cys of the dipeptide to spontaneously couple with thioesters, analogous to native chemical ligation (NCL)^27,28^. Such a route would also be highly convergent as any thioester could ligate with the core ʟ-Cys-ᴅ-Val dipeptide. Although ᴅ-Ala-ᴅ-Ala ligases are involved in peptidoglycan biosynthesis, ligases accepting ᴅ-amino acids are rare^29,30^. There are, on the other hand, many ʟ-amino acid ligases (Lal), belonging to the ATP-grasp family that have been shown to produce various dipeptides^31–35^. Of the Lals tested so far, TabS from *Pseudomonas syringae*, which is involved in the biosynthesis of tabtoxin (another β-lactam natural product), exhibits the broadest substrate scope^31,32^. Although not reported to generate ʟ-Cys-ʟ-Val, the promiscuity of TabS prompted us to test this enzyme first (Fig. 2a). Incubation of TabS with a 1:1 ratio of ʟ-Cys and ʟ-Val, along with ATP, resulted in ʟ-Cys-ʟ-Val and its disulphide dimer in ~40% yield, along with 13% of ʟ-Val-ʟ-Val side product. By adjusting the molar ratio to 2:1 and 3:1 ʟ-Cys to ʟ-Val, the yields of the dipeptide increased to 53% and 67%, respectively, with lower amounts of 6–7% of ʟ-Val-ʟ-Val evident (Supplementary Fig. 2). In addition to disulphide formation, HPLC purification of the product ʟ-Cys-ʟ-Val was complicated by its close retention time to ʟ-Val-ʟ-Val (Fig. 2b and Supplementary Fig. 16). Disulphide formation would not occur in the reducing environment of the cell and can be suppressed in vitro through the addition of reductants. Nevertheless, for synthetic expediency we also explored TabS ligation of ʟ-Val with ʟ-Thioproline (ʟ-SPro) formed from the condensation of ʟ-Cys with formaldehyde (Fig. 2a). Incubating ʟ-SPro and ʟ-Val (2:1) with TabS and ATP gave the dipeptide ʟ-SPro-ʟ-Val in a yield of 63%, with only a minor amount of ʟ-Val-ʟ-Val present (ca. 9%) (Fig. 2c). The 3:1 reaction mixture of ʟ-SPro and ʟ-Val afforded ʟ-SPro-ʟ-Val with a yield of 81% along with a 7.5% yield of ʟ-Val-ʟ-Val dipeptide (Supplementary Fig. 2). Given that this dipeptide product is more easily purified due to the absence of disulphide formation, the TabS ligation of ʟ-SPro and ʟ-Val can be carried out on a preparative scale (Supplementary Fig. 17). Incubation of ʟ-SPro-ʟ-Val with methoxyamine hydrochloride (MeONH_2_.HCl) and TCEP, afforded ʟ-Cys-ʟ-Val in >95% yield (Supplementary Fig. 3).

### Valine epimerization

Enzymes from the enolase superfamily have been identified that epimerise the C-terminal residues of dipeptides, including ʟ-Ala-ᴅ/ʟ-Glu epimerase from *B. subtilis* or *E. coli*, which are proposed to be involved in recycling peptidoglycan derived from bacterial cell walls^36,37^. Genome mining and structural studies reveal that many diverse dipeptide epimerases are present in bacteria, which are predicted to possess different dipeptide specificity^38,39^. One of these epimerases from *Thermotoga maritima* was shown to epimerise a wide range of dipeptide substrates, including dipeptides possessing hydrophobic amino acids at the C-terminus. Although the *T. maritima* epimerase had not been shown to epimerise ʟ-Cys-ʟ-Val, we performed molecular docking studies using its available crystal structure (PDB: 3DEQ)^38^, which was solved in complex with ʟ-Ala-ʟ-Leu ligand. The docking studies suggested that the enzyme is likely to accommodate ʟ-Cys-ʟ-Val within its active site (Supplementary Fig. 4). As predicted, the *T. maritima* epimerase does epimerise ʟ-Cys-ʟ-Val generating ʟ-Cys-d-Val as the major diastereomer (~80%) (Fig. 2d). We also incubated ʟ-Cys-d-Val with the epimerase which also resulted in the same ratio of epimers (80:20 LD vs LL) confirming that the thermodynamic equilibrium position had been reached and this favours the desired epimer (Fig. 2d). The *T. maritima* epimerase showed very low activity with the ʟ-SPro-ʟ-Val dipeptide and we therefore proceeded to the next step with ʟ-Cys-d-Val dipeptide.

### Dipeptide *N*-acylation

**Fig. 3:**
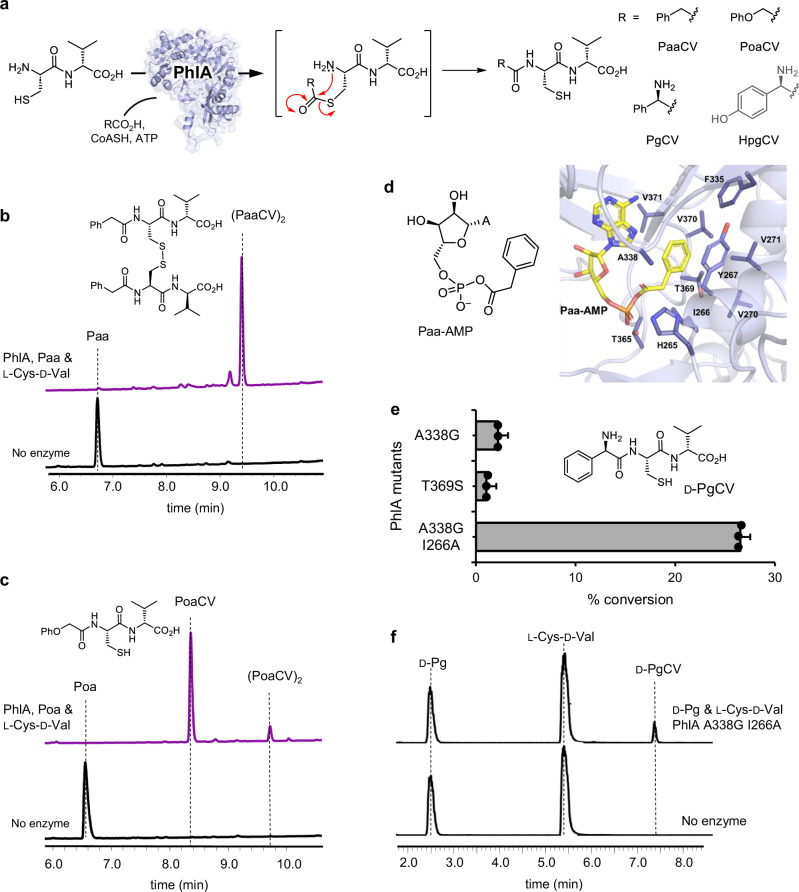
*N*-acylation of ʟ-Cys-ᴅ-Val dipeptide by CoA ligase enzyme. **a** Scheme to synthesise tripeptide-like molecules from phenylacetic acid, phenoxyacetic acid, d-Phenylglycine or d-Hydroxyphenylglycine by a CoA ligase-mediated thio-esterification and subsequent native chemical ligation with ʟ-Cys-d-Val. HPLC profiles of the in vitro assays with PhlA CoA ligase, ʟ-Cys-d-Val and (**b**) phenylacetic acid (Paa), or (**c**) phenoxyacetic acid (Poa). **d** AlphaFold model of PhlA CoA ligase with the intermediate phenylacetyl adenylate (Paa-AMP) docked in the putative active site. **e** The % conversion (determined in triplicate by LC–MS using calibration curves with synthetic standards; data are represented as mean values (*n* = 3) and error bars represent standard error) for the ligation of d-Pg and ʟ-Cys-d-Val forming ᴅ-PgCV catalysed by PhlA mutants. **f** LC-MS (EIC) trace showing the formation of ᴅ-PgCV by the PhlA mutant A338G I266A. Source data are provided as a Source Data file.

The adenylate-forming (ANL) superfamily of enzymes includes many acyl-CoA synthetases (ACS) that produce acyl-CoA thioesters via acyl-AMP intermediates^40^. The acyl-CoAs can serve as substrates for the acyltransferase enzyme that transfers the acyl substituent from coenzyme A (CoASH) to a nucleophilic acceptor substrate^41^. The presence of the N-terminal Cys residue in dipeptide acceptor substrate (ʟ-Cys-d-Val) precludes the need for an acyl transferase. Provided a CoA ligase can efficiently thiolate the acyl donor, the resulting acyl-CoA should undergo spontaneous *trans*-thioesterification with the N-terminal Cys thiol group and a subsequent intramolecular S- to *N*-acyl shift^27,28^ to afford the desired *N*-acylated-ʟ-Cys-ᴅ-Val (Fig. 3a). We chose to explore the activity of PhlA (Pc22g14900) from *Penicillium chrysogenum,* which activates phenylacetic acid (Paa), producing the phenyl acetyl-CoA thioester (Paa-CoA), which serves as a substrate for isopenicillin *N*-acyltransferase (IAT) in penicillin G biosynthesis^42–44^. In addition to PhlA, three other acyl-CoA synthetases were selected from *P. chrysogenum* [PhlB, PhlC and ary4 (Pc21g20650)] (Supplementary Fig. 5a). The four ligases were each incubated with Paa or phenoxyacetic acid (Poa, side chain acyl group of penicillin V), coenzyme A, ATP and the ʟ-Cys-d-Val dipeptide. Of the four tested, PhlA gave the highest conversions to the *N*-acylated products (Supplementary Fig. 5b, c). Paa-ʟ-Cys-d-Val (PaaCV) was produced in 69.9 ± 2.5%, predominantly as the disulphide dimer (PaaCV)_2_ (Fig. 3b and Supplementary Fig. 6), whilst Poa-ʟ-Cys-d-Val (PoaCV) was produced in 81.7 ± 1.9 % largely as the free thiol (Fig. 3c and Supplementary Fig. 7). The PhlA reactions were performed on a preparative scale, providing PaaCV and PoaCV with 40% and 52% isolated yields, respectively (Supplementary Figs. 18–19). Additionally, starting from ʟ-Cys-ʟ-Val, we also demonstrated a preparative-scale enzyme cascade by combining the epimerase and PhlA enzymes to synthesise PaaCV and PoaCV in isolated yields of 30% and 34%, respectively (Supplementary Figs. 20–21).

PaaCV and PoaCV can serve as precursors for Penicillin G and V, respectively. In addition, we also sought to develop routes to tripeptide precursors for ampicillin, ᴅ-phenylglycine-ʟ-Cys-ᴅ-Val (PgCV) and for amoxicillin, ᴅ-hydroxyphenylglycine-ʟ-Cys-ᴅ-Val (HpgCV). The wild-type PhlA enzyme cannot activate either ᴅ-phenylglycine (ᴅ-Pg) or ᴅ-hydroxyphenylglycine (ᴅ-Hpg). However, PhlA does exhibit moderate activity with ᴅ-phenylalanine (ᴅ-Phe)^44^, generating the tripeptide ᴅ-Phe-ʟ-Cys-ᴅ-Val in the presence of coenzyme A and ʟ-Cys-ᴅ-Val (Supplementary Fig. 8). Considering this, we envisaged that rational engineering of PhlA could alter its selectivity towards ᴅ-Pg and ᴅ-Hpg. As there is no crystal structure of PhlA from *Penicillium chrysogenum* available, we generated an AlphaFold model of PhlA, with the adenylate intermediates of its native substrate (Paa-AMP) docked into the active site. The structural model revealed 11 amino acid residues in the large N-terminal domain lining the Paa binding pocket (Fig. 3d). The non-canonical substrates ᴅ-Pg and ᴅ-Hpg are structurally similar to Paa but contain an additional α-amine moiety. To accommodate ᴅ-Pg in the PhlA substrate binding pocket, we initially selected residues I266, A338, T365 and T369, which are located near the CH_2_ moiety, for mutagenesis. Five single-point mutants (I266A, A338G, T365A, T369S and T369A) were constructed and tested for activity against ᴅ-Pg. Among these, the A338G and T369S variants unlocked the activity with ᴅ-Pg, forming the tripeptide PgCV with 1–2% conversion (Fig. 3e). We further engineered PhlA by introducing a series of double mutations on top of the A338G variant, identifying A338G/I266A as a significantly improved variant, producing PgCV with 26.6 ± 0.03% conversion (Fig. 3e, f and NMR spectra in Supplementary Fig. 22). Additional rounds of mutagenesis did not yield further improvements in activity with ᴅ-Pg (Supplementary Table 1). Also, none of the mutant PhlA enzymes exhibited activity with ᴅ-Hpg, likely due to the presence of the phenol side chain. In the PhlA active site, the aromatic ring of the Paa-AMP intermediate is predominantly surrounded by hydrophobic residues, including V270, V271, F335, V370 and V371 (Fig. 3d). Further engineering of the PhlA active site would likely be required to accommodate the more polar phenol group of ᴅ-Hpg.

### Cyclisation of peptides into β-lactam antibiotics

**Fig. 4:**
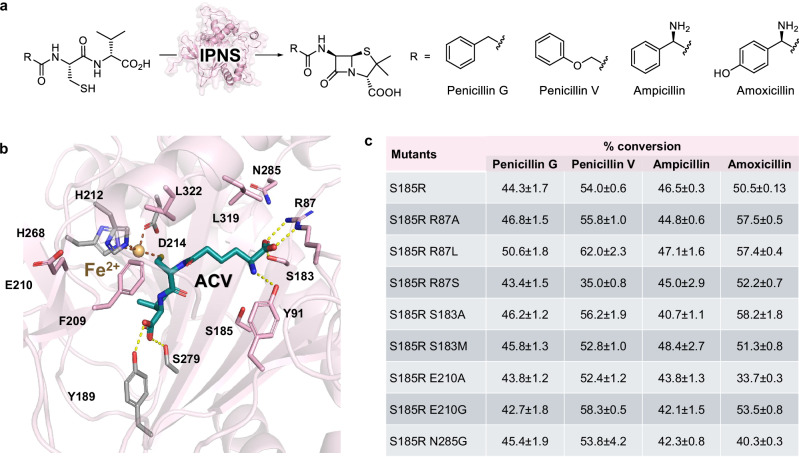
Cyclisation of *N*-acyl-l-Cys-d-Val tripeptide using isopenicillin N synthase (IPNS). **a** IPNS-mediated formation of different β-lactam antibiotics from precursor *N*-acyl-ʟ-Cys-d-Val peptides. **b** AlphaFold 3 model of caIPNS bound to ACV and iron. **c** % conversion of penicillin G, penicillin V, Ampicillin and Amoxicillin formed by caIPNS mutants from *N*-acyl-ʟ-Cys-d-Val peptide precursors (determined in triplicate by LC–MS using calibration curves with synthetic standards; data are represented as mean values (*n* = 3). Source data are provided as a Source Data file.

The next and final step is the bicyclisation of *N*-acyl-ʟ-Cys-ᴅ-Val precursors into penicillin variants (Fig. 4a). For this purpose, we envisaged using isopenicillin N synthase (IPNS), from the native penicillin pathway^11–13,45–47^. The IPNS enzyme is a non-heme, Fe^2+^-dependent oxidase enzyme that catalyses the formation of isopenicillin N from the ACV tripeptide (ʟ-α-Aminoadipyl-ʟ-Cys-ᴅ-Val) in the presence of O_2_ (Fig. 1a)^11–13,48^. Early studies indicated that this enzyme could tolerate some variation at the N-terminus of the tripeptide substrate and the *Streptomyces lactamdurans* IPNS has been used to cyclise the penicillin G precursor, PaaCV, with 20-times less efficiency than that for ACV tripeptide^49,50^. We thus set out to engineer IPNS to accept non-native peptides. The IPNS enzyme has been found in both bacteria and fungi. In our initial studies, we used a codon-optimised IPNS from *Streptomyces clavuligerus* (scIPNS). Previously, mutagenesis of a fungal IPNS from *Aspergillus nidulans* (anIPNS) identified a mutant V185R, which has a broader substrate scope^51^. Based on this, the equivalent position S185 of the bacterial scIPNS was mutated to Lys, Arg, Glu, His, Asn and Gln, with S185R showing the highest activity with alternative substrates PaaCV and PoaCV. The introduction of the S185R mutation did, however, reduce levels of protein production significantly. Consequently, we explored the production levels of various alternative bacterial IPNS orthologues and observed that IPNS from *Streptomyces cattleya* (caIPNS) was produced at the highest level (Supplementary Table 2). Additionally, IPNS enzymes appear to be unstable under conventional in vitro assay conditions employing Fe²⁺/ascorbate. This instability is most likely due to the oxidative stress generated from Fe/ascorbate-induced ROS, which may inactivate the enzyme over the assay time scale^52^. To mitigate this issue, catalase was included in the assay mixture, which significantly improved productivity compared to the standard Fe²⁺/ascorbate buffer (Supplementary Fig. 9). The caIPNS wt and S185R mutant were then screened with the four peptide precursors PaaCV, PoaCV, PgCV and HpgCV. LC-MS analysis indicated that wt caIPNS did not exhibit any catalytic activity towards any of the non-native peptides, but the caIPNS S185R resulted in the formation of penicillin G, penicillin V, ampicillin and amoxicillin in promising yields of 44.3 ± 1.7%, 54.0 ± 0.6%, 46.5 ± 0.3% and 50.5 ± 0.13% yield (Supplementary Figs. 10–11), respectively.

To identify more efficient IPNS variants for non-native peptides, we expanded our mutagenesis beyond the S185R mutation. An AlphaFold3 model of caIPNS bound to its native substrate ACV and Fe^2+^ was generated using the Protenix server^53^. This model revealed a similar constellation of active-site residues as in the crystal structure of fungal anIPNS (PDB 1BK0)^11^ (Fig. 4b and Supplementary Fig. 12). Based on proximity to the α-aminoadipic acid side chain, we selected residues R87, Y91, S183, N285 and L319 for mutagenesis. Additionally, we targeted residues F209, E210 and L322 near the C-terminus, since the C-terminal glutamine that coordinates the iron is displaced upon substrate binding^11^. Each of these residues was individually randomised using the NDT codon. The resulting mutant libraries were screened against HpgCV using cell lysates and product formation was quantified by HPLC in multiwell plates. Sequencing identified 8 mutants with improved amoxicillin production. These double mutants were purified and tested against four non-native substrates. Among them, the S185R R87L variant showed the highest activity with PaaCV, PoaCV and HpgCV (Fig. 4c), yielding Penicillin G (50.6 ± 1.8% conversion), Penicillin V (62 ± 2.3% conversion) and Amoxicillin (57.4 ± 0.4% conversion) (Supplementary Figs. 10–11). However, for PgCV, this double mutant displayed comparable activity to S185R, with ca. 47% ampicillin formation. The S185R mutation likely improves accommodation of the non-native *N*-acyl-ʟ-Cys-ᴅ-Val substrates in the IPNS active site, while the R87L mutation may enhance activity by eliminating a mismatched positive charge and introducing a hydrophobic pocket favourable for phenyl-containing substrates. Supporting this, S185R R87A/L/S variants were inactive toward the native ACV tripeptide, whereas mutants such as S183A/M, E210A/G and N285G retained activity with the native substrate (Supplementary Fig. 13). In summary, minimal structure-guided engineering allowed us to identify single and double point IPNS mutants capable of processing diverse non-native *N*-acyl-ʟ-Cys-ʟ-Val substrates. While our efforts focused on producing penicillins with known antimicrobial activity, the substrate flexibility of these IPNS mutants suggests a broader potential.

### Biocatalytic cascades for penicillin production

**Fig. 5:**
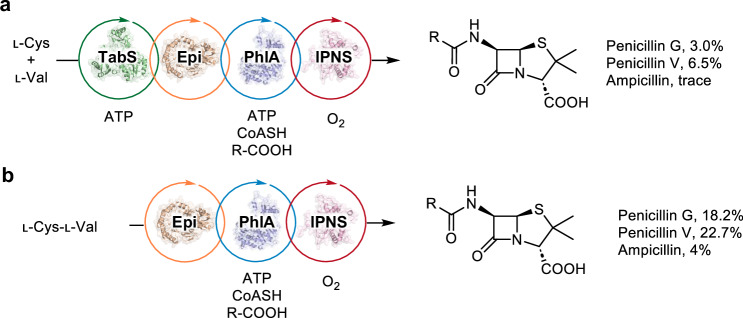
Schematic representation of the engineered biocatalytic cascades for penicillin synthesis. **a** The complete four-enzyme cascade (top) comprises TabS, epimerase (Epi), PhlA CoA ligase (PhlA) and isopenicillin N synthase (IPNS) enzymes (yields are calculated based on the limiting substrate ʟ-Val). To improve the overall efficiency, (**b**) a modified three-cascade excluding the TabS step was implemented (bottom), initiating from the epimerase reaction and proceeding through PhlA and IPNS, resulting in improved penicillin yields (calculated based on ʟ-Cys-ʟ-Val).

After establishing each enzymatic step of the alternative pathway, we sought to develop a four-enzyme cascade for de novo total in vitro biosynthesis of β-lactam antibiotics (Fig. 5a). ʟ-Cys and ʟ-Val were first incubated with ATP and TabS. When the ligation was complete, the epimerase was then added, along with additional TCEP to reduce any disulphides formed. Following epimerisation, the third enzyme (PhlA) is introduced to the reaction mixture, accompanied by a carboxylic acid substrate (Poa, Paa or d-Pg) and more cofactors (ATP and CoASH). After a further incubation period, the reaction mixture was extracted with an organic solvent and the crude *N*-acyl-l-Cys-d-Val intermediates were re-dissolved in fresh buffer containing IPNS and catalase to afford the final β-lactam antibiotics. The solvent extraction procedure was necessary because IPNS is inactivated by TCEP and possibly other components of the previous enzymatic reactions. Using this two-part four enzyme cascade process, the three antibiotics were successfully produced, albeit in low overall yields, with penicillin G and penicillin V obtained in 3.0% and 6.5% yield, respectively and ampicillin detected only in trace amounts. The progress of the cascade was monitored at each stage by LC-MS (Supplementary Fig. 14).

To improve the efficiency of the cascade, we evaluated the catalytic rates of the individual enzymes. This revealed that TabS has substantially lower activity than other enzymes, while IPNS also displayed moderate activity with non-native substrates (Supplementary Table 3). Moreover, the large excess of ʟ-Cys required in the TabS ligation led to the formation of side products that interfered with downstream transformations. We therefore implemented an alternative three-enzyme cascade that excluded the initial TabS ligation (Fig. 5b). Starting from ʟ-Cys-ʟ-Val, this shorter cascade resulted in 3–6 fold improved overall yields, affording penicillin G, penicillin V and ampicillin in 18.2%, 22.7% and 4% yields respectively (Supplementary Fig. 15). We anticipate that future protein and reaction-engineering efforts will be required to improve the performance of the cascade reactions. For example, engineering TabS to improve its catalytic activity and selectivity may enable the use of stoichiometric amounts of ʟ-Cys and ʟ-Val, which should significantly benefit the cascade processes. Further IPNS engineering is also likely to be necessary. Finally, the cascades presented here have not been fully optimised and could potentially be improved by more systematic process development, including the use of immobilised enzymes, flow processes and/or co-factor recycling systems^54,55^. Extension to an in vivo cascade may be feasible in the future. However, the use of NCL could be complicated due to endogenous thioesters (e.g., acetyl-CoA) and may require a different ligase enzyme for *N*-acylation of the l-Cys-d-Val precursor.

## Discussion

Penicillin is derived from a peptide precursor assembled by ACVS (Fig. 1a), a complex NRPS enzyme comprising ten distinct domains, that requires post-translational modification to function. In this study, we demonstrate an alternative enzymatic route to penicillin antibiotics, including simpler stand-alone ligase and epimerase enzymes, to generate penicillin derivatives. An ATP-grasp ligase (TabS) was employed to condense l-Cys and l-Val, resulting in the dipeptide ʟ-Cys-l-Val. This dipeptide was subsequently epimerised to l-Cys-d-Val by an epimerase from *T. maritima*, with the thermodynamic equilibrium favouring the l-Cys-d-Val diastereoisomer (80%). Acyl-CoA synthetase (PhlA) is then used to acylate the dipeptide, exploiting *S*- to *N*-acyl migration, to generate *N*-acyl-l-Cys-d-Val peptides, which are cyclised to penicillin derivatives by IPNS variants. This alternative pathway offers greater flexibility than the native NRPS system, affording access to non-natural penicillin that are currently produced by a semi-synthetic route. Furthermore, we demonstrate that the new enzymes can be deployed in cascade reactions to produce the target penicillin, without the isolation and purification of intermediates. With further protein engineering, it may be possible to improve the selectivity and efficiency of the enzymes, particularly TabS and IPNS, to enhance the outputs of these cascade reactions. To date, there is only one other example, we are aware of, where an entirely de novo enzyme cascade has been developed as a possible route to a commercially relevant synthetic pharmaceutical, islatrivir^56^. In this example, many rounds of directed evolution were required to optimise each enzyme in a two-part cascade that delivered islatrivir in 51% overall yield, with one intermediate isolation/purification step.

In addition to the penicillin antibiotics, there are also many other peptide-derived therapeutic agents currently used in the clinic. Most of the approved peptide-based pharmaceuticals are produced by chemical synthesis, which is step- and atom-inefficient, relying on costly protected amino acid precursors, deleterious reagents and large volumes of harmful organic solvents. Alternative enzymatic methods for assembling peptides and derivatives would be valuable. Most peptides present in nature are, however, produced by ribosomes and NRPS enzymes, which work well in a cell, but can be difficult to engineer and reconstitute in vitro. We envisage that simpler ATP-dependent ligase enzymes and possibly epimerases, such as those described here, may be more versatile, easier to engineer and could offer novel pathways to many peptide therapeutics or other useful peptide products^57–59^.

## Methods

### General procedures

Chemicals, molecular biology reagents, media components, buffers and solvents were obtained from Sigma Aldrich, Fluorochem, Fisher Scientific, Formedium and New England Biolabs. The dipeptide and tripeptide precursors were obtained from Bachem and Biomatik. Primers were procured from Integrated DNA Technologies (IDT) and codon-optimised synthetic genes were purchased from Twist Bioscience. DNA sequencing was performed by Eurofins Genomics. Sterilisation was performed by autoclaving at 121 °C for 15 min. *E. coli* strains were stored at −80 °C in 30% (v/v) glycerol. Microbial cultures were incubated in Innova 44 shaking incubators. Analytical RP-HPLC was performed using Shimadzu UFLC-XR Analytical HPLC with a Diode Array Detector. Semi-preparative HPLC was performed on a Shimadzu Preparative HPLC system. Data analysis for RP-HPLC was carried out with the in-built Shimadzu LabSolutions Software. The LC–MS was performed on an Agilent 6560/6546 quadrupole time-of-flight (Q-TOF)/Agilent 1290 Infinity LC system and the data were analysed by Qualitative Analysis B.06.00 software. NMR spectra were recorded on Bruker Avance III spectrometers (400 MHz and 500 MHz) with TopSpin and IconNMR. NMR data were processed using MestReNova version 11 software.

### Protein expression and purification

BL21(DE3) competent cells were transformed with plasmids encoding the gene of interest. A 10 mL culture of LB with appropriate antibiotic was inoculated with a single colony of *E. coli* BL21(DE3) cells containing the plasmid and incubated overnight at 37 °C with 180 rpm shaking. 8 mL of this pre-culture was used to inoculate 0.8 L 2×YT-autoinduction medium (Formedium) in 2 L baffled flasks supplemented with 2 drops of Sigma Antifoam 204 and antibiotic (100 μg/mL Ampicillin for pET21 constructs and 50 μg/mL Kanamycin for pET28 constructs). The cultures were incubated at 37 °C for 5 h, then at 18 °C for a further 20 h with 180 rpm shaking. Cell pellets were then harvested by centrifugation, washed with 1× phosphate-buffered saline (PBS) and frozen at −20 °C. The pellet from 0.8 L of culture was resuspended in 40 mL of lysis buffer (50 mM Tris.HCl, pH 8.0, 300 mM NaCl, 20 mM imidazole, 10% (v/v) glycerol) and sonicated on ice using a 4-tip sonicator (Qsonica Q500 system) at 50% power for 30 min in bursts of 5 s followed by 5 s of cooling. The lysates were clarified by centrifugation at 14000 × *g* for 45 min at 4 °C and the supernatant was collected and combined with Ni-NTA agarose (Qiagen), pre-equilibrated in lysis buffer. After mixing for a minimum of 30 min at 4 °C, the suspension was passed through a gravity-flow column. The resin was then washed with 20 column volumes of wash buffer (50 mM Tris.HCl, pH 8.0, 300 mM NaCl, 30 mM imidazole, 10% (v/v) glycerol) and then eluted with 10 column volumes of elution buffer (50 mM Tris.HCl, pH 8.0, 300 mM NaCl, 250 mM imidazole, 10% (v/v) glycerol). The eluted protein was then loaded into a Vivaspin 20 centrifugal concentrator MWCO 30 kDa (Sartorius) and concentrated by centrifugation at 3500 × *g* at 4 °C. Afterward, buffer exchange was performed with storage buffer (50 mM Tris.HCl, pH 8.0, 100 mM NaCl, 10% (v/v) glycerol) to remove imidazole and the protein solution was again concentrated using the centrifugal concentrator. To confirm the purity of the protein in the solution, sodium dodecyl sulfate-polyacrylamide gel electrophoresis (SDS-PAGE using ThermoFisher Novex WedgeWell 4–16% Tris-glycine gels) was performed. The protein concentration was then determined by nanodrop measurement of A280 using the extinction coefficients calculated using the ProtParam tool. The concentrated protein solutions were then frozen rapidly in liquid nitrogen and were stored at −80 °C.

### TabS assay

The standard assay mixture for TabS (200 µL total volume) contained 10 mM of ʟ-Cys/ʟ-SPro, 5 mM of ʟ-Val, 10 mM ATP (pH 9.0), 10 mM MgCl_2_, 2 mM of TCEP (pH 9.0) and 10 μM of TabS in 50 mM Tris.HCl buffer (pH 9.0). The reaction was incubated at 35 °C with shaking at 600 rpm. After 20 h, an equal volume of methanol was added to the reaction mixture and then centrifuged at 14000 × *g* for 10 min and the supernatant was then analysed by high-resolution liquid chromatography-mass spectrometry (positive mode Q-TOF LC-MS, Agilent Technologies). The analytical conditions were as follows: Phenomenex HILIC Luna 200 Å column (5 µm, 150 × 4.6 mm); mobile phase A—1:1 mixture of 10 mM ammonium formate, pH 3.0 and acetonitrile and mobile phase B—9:1 mixture of acetonitrile and 10 mM ammonium formate, pH 3.0 (0–1 min, 70% B; 1–7 min, 70–40% B; 7–8 min, 40–10% B; 8–12 min, 10% B; 12–13 min, 10–70% B and finally the column equilibration step 13–20 min, 70% B); flow rate, 0.6 ml/min; column temperature 40 °C.

### Epimerase assay

The epimerisation assays were carried out in 20 mM Tris.HCl buffer, pH 8.0 containing 1 mM of dipeptide substrate (reference standard ʟ-Cys-ʟ-Val was acquired from Biomatik), 10 mM MgCl_2_, 2 mM TCEP (pH 8.0) and 5 μM enzyme (50 µL total volume). The reaction was incubated at 40 °C for 16 h with shaking at 600 rpm. The reaction mixture was then resuspended in an equal volume of methanol, centrifuged at 14000 × *g* for 10 min and the supernatant was collected and analysed by positive mode high-resolution LC-MS. The analytical conditions were: Phenomenex Luna Omega Polar C18 100 Å column (5 µm, 100 × 2.1 mm); mobile phase A—water + 0.1 % formic acid and mobile phase B—MeOH + 0.1 % formic acid (0–1 min, 5% B; 1–9 min, 5–95% B; 9–10 min, 95% B; 10–11 min, 95–5% B; 11–12 min, 5% B); 0.5 mL/min flow rate and column temperature 40 °C.

### CoA ligase assay

Assays with CoA ligases (200 µL total volume) were set up using 3 mM ATP (pH 8.0), 1.5 mM Coenzyme A (CoASH), 10 mM MgCl_2_, 3 mM Phenylacetic acid (Paa) or Phenoxyacetic acid (Poa) (pH 8.0), 2 mM TCEP (pH 8.0), 3 mM ʟ-Cys-ᴅ-Val (standard reference ʟ-Cys-ᴅ-Val was obtained from Bachem) and 4 µM CoA ligase enzyme in 50 mM Tris.HCl buffer (pH 8.0). The reaction mixtures were incubated overnight at 30 °C with shaking at 600 rpm. After 16–18 h incubation, the products were acidified, extracted with ethyl acetate, evaporated and dissolved in 100% methanol and then analysed by HPLC and LC-MS.

HPLC condition: Phenomenex Kinetex XB-C18 100 Å column (5 µm, 100 × 4.6 mm); mobile phase A—water + 0.1 % formic acid and mobile phase B—methanol + 0.1 % formic acid (0–1 min, 5% B; 1–9 min, 5–95% B; 9–12 min, 95% B; 12–13 min, 95–5% B; 13–15 min, 5% B); 0.5 mL/min flow rate and column temperature 40 °C.

LC-MS condition: Phenomenex Luna Omega Polar C18 100 Å column (5 µm, 100 × 2.1 mm); mobile phase A—water + 0.1 % formic acid and mobile phase B—MeOH + 0.1 % formic acid (0–1 min, 5% B; 1–9 min, 5–95% B; 9–10 min, 95% B; 10–11 min, 95–5% B; 11–12 min, 5% B); 0.5 mL/min flow rate and column temperature 40 °C.

In case of ᴅ-PgCV, assays were set up containing 3 mM ATP (pH 8.0), 1.5 mM CoASH, 10 mM MgCl_2_, 1 mM ᴅ-Phenylglycine (ᴅ-Pg) (pH 8.0), 2 mM TCEP (pH 8.0), 1 mM ʟ-Cys-ᴅ-Val and 10 µM CoA ligase enzyme in 50 mM Tris.HCl buffer (pH 8.0). The reaction mixtures were incubated overnight at 30 °C with shaking at 600 rpm. After 16–18h incubation, the samples were mixed with an equal volume of methanol, centrifuged at 14000 × *g* for 10 min, and the supernatant was collected and analyzed by LC-MS.

LC-MS condition: Phenomenex Kinetex XB-C18 100 Å column (2.6 µm, 100 × 4.6 mm); mobile phase A—water + 0.1 % formic acid and mobile phase B—MeOH + 0.1 % formic acid (0–1 min, 5% B; 1–9 min, 5–95% B; 9–10 min, 95% B; 10–11 min, 95–5% B; 11–12 min, 5% B); 0.5 mL/min flow rate and column temperature 40 °C.

### IPNS assay

For IPNS assay, 0.5 mM ACV (purchased from Bachem) or PoaCV/PaaCV (synthesised in lab) or HpgCV/PgCV (obtained commercially from Bachem & Biomatik, respectively) was pre-incubated with 1 mM DTT in Tris.HCl storage buffer (50 mM Tris.HCl, pH 8.0, 100 mM NaCl, 10% (v/v) glycerol) for 1 h to keep the precursor tripeptide in a reduced state (100 µL total volume). After 1 h incubation, 50U/µL catalase (Sigma) and 5 or 10 µM IPNS (5 µM IPNS for ACV assay and 10 µM IPNS for alternative substrates) were added and the reactions were incubated at 25 °C for 2 h. The assays were then quenched by addition of 1 volume of acetonitrile (ACN), centrifuged at 14000 × *g* for 10 min at 4 °C. The supernatant was then collected and analysed by high-resolution positive mode Q-TOF LC-MS. The LC-MS conditions were: mobile phase A- water + 0.1 % formic acid and mobile phase B- ACN + 0.1 % formic acid (0–1 min, 5% B; 1–9 min, 5–95% B; 9–10 min, 95% B; 10–11 min, 95–5% B; 11–12 min, 5% B); 0.5 mL/min flow rate and column temperature 25 °C. For the ACV/HpgCV/PgCV-IPNS assay, a Phenomenex Kinetex XB-C18 100 Å column (2.6 µm, 100 × 4.6 mm) was specifically used.

### Reporting summary

Further information on research design is available in the Nature Portfolio Reporting Summary linked to this article.

## Supplementary information

Supplementary Information

Reporting Summary

Transparent Peer Review file

## Source data

Source Data

## Data Availability

The amino acid sequences of the enzymes used in this study are presented in the Supplementary Information file. Enzyme structures used for modeling and docking studies can be accessed on rcbs.org using PDB IDs that are mentioned in the article. The AlphaFold structure of PhlA can be accessed on https://alphafold.ebi.ac.uk/entry/B6HVC6 (AF-B6HVC6-F1-v4). All the remaining data are available in the main text, the supplementary materials and from the corresponding author upon request. Source data are also provided with this paper. Correspondence and requests for materials should be addressed to J.M. Source data are provided with this paper.
