# Supplementary material for: Alternative enzymatic pathways to penicillin antibiotics
Source: Nat Commun. 2026 Apr 30;17:5891. doi: 10.1038/s41467-026-72466-w (PMC13338154; doi:10.1038/s41467-026-72466-w)
Supplement: Supplementary file 1 — Supplementary Information [file 41467_2026_72466_MOESM1_ESM.pdf]

Supplementary Information

Alternative Enzymatic Pathways to Penicillin Antibiotics

Puja Saha,[1,2] Guangcai Xu,[1,2] Deepanjan Panda,[1,2] Duncan Smith,[2] Wei Li Thong,[2] Luke Ward,[2] Luis Bering,[2] Sebastian Cuesta-Hoyos,[2] Sarah A. Shepherd,[2] & Jason Micklefield*[1,2]

[1]Department of Chemistry, Imperial College London, Molecular Sciences Research Hub, 82 Wood Lane, London W12 0BZ, UK.

[2]Department of Chemistry and Manchester Institute of Biotechnology, The University of Manchester, 131 Princess Street, Manchester M1 7DN, UK.

*Corresponding authors Email:
j.micklefield@imperial.ac.uk

Table of Contents

Supplementary Method 2
- **Cloning & Mutagenesis** 2
- **Computational Modelling & Docking Studies** 2
- **Chemical conversion of L-SPro-L-Val to L-Cys-L-Val dipeptide** 3
- **Biocatalytic cascades for penicillin synthesis** 3
- **Measurement of specific activity of enzymes** 4
- **Preparative scale enzymatic reactions** 5
- **General synthetic route for PoaCV and PaaCV** 8
- **Enzyme Sequences** 13
- **Primers used for PhlA mutation** 15

Supplementary Tables & Figures 16
- **NMR spectra of enzymatically synthesised compounds** 30
- **NMR spectra of chemically synthesised compounds** 41

Supplementary References 52

Supplementary Method

Cloning & Mutagenesis

Genes encoding wild-type TabS, Epimerase, CoA ligases and isopenicillin N synthase (IPNS) were procured either with N-terminal or C-terminal His_6 tag from Twist Bioscience. Sequences were codon-optimised using Twist's proprietary algorithms and cloned into either pET28 or pET21 vectors. *E. coli* DH5α cells were used for plasmid storage and *E. coli* BL21(DE3) cells were used for protein production. Site-directed mutagenesis for PhlA was performed using the NEB Q5 mutagenesis protocol with primers designed via NEBaseChanger.

Screening of IPNS mutant library: Amino acid residues near the α-aminoadipic acid moiety of ACV substrate in the IPNS active site were targeted for site-saturation mutagenesis. The plasmid libraries were created by NEB Q5 mutagenesis procedure using primers containing NDT codons and each site was targeted individually. After PCR amplification, the PCR products were subjected to KLD reaction (Q5 Site-Directed mutagenesis kit) and subsequently used to transform the chemically competent *E. coli* BL21(DE3) cells. Individual colonies were picked into the wells of a 96 deep-well-plate containing 0.5 mL LB medium supplemented with antibiotic and incubated at 37°C overnight. These were sub-cultured into 96 deep-well-plates filled with 1.2 mL of 2×YT autoinduction medium, which were incubated at 37°C for 5 hours followed by at 18°C for 20 hours. Glycerol stocks were prepared by mixing 100 µL of overnight culture with 100 µL of 60% (v/v) glycerol, sealing the plates with sterile adhesive foils, and storing them at -80°C. Cell pellets from the remaining culture were harvested by centrifugation and stored at -20°C. The plates were thawed at room temperature for 30 minutes, then the pellets were resuspended in 150 µL of B-PER reagent (Thermo Scientific™). After 30 minutes at room temperature, 20 µL of lysate was combined with 30 µL of IPNS assay mix in Tris.HCl storage buffer (50 mM Tris.HCl, pH 8.0, 100 mM NaCl, 10% (v/v) glycerol) to give an assay mixture containing 4 mM HpgCV, 4 mM DTT, and 50U/µL catalase solution. The assays were incubated at 25°C for 30 minutes and quenched by addition of 50 µL of methanol. After 5 minutes, 150 µL of water was added and the samples was centrifuged for 30 minutes at 4°C. The supernatants were transferred to a clean 384-well plate for LC analysis.

Computational Modelling & Docking Studies

T. maritima epimerase: A model of L-Cys-L-Val was docked into the X-ray crystal structure of *Thermotoga maritima* epimerase (PDB: 3DEQ)[S1]. The grid box was positioned over the active site with dimensions of 17 Å × 17 Å × 18 Å along the X, Y, and Z axes, respectively. A spacing of 0.5 Å was used, and the exhaustiveness parameter was set to 32. Full flexibility was allowed for the L-Cys-L-Val dipeptide. The calculation was performed using Autodock4[S2].

PhlA CoA ligase: A model of Paa-AMP was docked into the AlphaFold predicted structure of PhlA (AF-B6HVC6-F1-v4, https://alphafold.ebi.ac.uk/entry/B6HVC6). The docking pocket was identified by examining the active site in Chain A of the X-ray crystal structure of *Nicotiana tabacum* 4-coumarate CoA ligase (PDB: 5BSW)[S3]. The centre of the calculation box was set at coordinates X = 18.94, Y = 0.66, and Z = −12.81, with a box size of 18 Å and a grid spacing of 1

Å. The exhaustiveness parameter was set to 32, and full flexibility was enabled for the Paa-AMP molecule. The calculation was performed using Autodock VINA.[S4]

Isopenicillin N synthase (IPNS): The structural model of IPNS from *Streptomyces cattleya* (caIPNS) bound to its native substrate ACV (L-α-aminoadipoyl-L-cysteinyl-D-valine) and a catalytic Fe^{2+} ion was generated using the Protenix server (https://protenix-server.com), which implements AlphaFold3 for protein-ligand complex prediction. The IPNS amino acid sequence was submitted with ACV specified as ligand and Fe^{2+} as a metal ion. The prediction was performed with the multiple sequence alignment option enabled (use msa = true), and using a model seed of 28259. The resulting model (sample 0) was aligned with the crystal structure of fungal IPNS (PDB: 1BK0)[S5] using PyMOL (v.2.5, Schrödinger LLC) for comparative analysis of the substrate binding pocket.

Chemical conversion of L-SPro-L-Val to L-Cys-L-Val dipeptide

50 mM L-SPro-L-Val dipeptide was dissolved in a 200 mM solution of disodium hydrogen phosphate (Na_2HPO_4) containing 100 mM TCEP.HCl and 200 mM methoxyamine hydrochloride ($MeONH_2$.HCl). The pH of the solution was then adjusted to 4.0 using HCl and the mixture was agitated at 70 °C for 18 h to afford the L-Cys-L-Val product.

Biocatalytic cascades for penicillin synthesis

Four-enzyme cascade assay: The cascade assay was initiated with an assay mixture (1 ml total volume) consisting of 10 mM L-Cys, 5 mM L-Val, 10 mM ATP (pH 9.0), 10 mM $MgCl_2$, 4 mM of TCEP (pH 9.0) and 10 μM TabS in 50 mM Tris.HCl buffer (pH 9.0). The reaction was incubated at 35°C with shaking at 600 rpm for 16 h. The reaction mixture was then adjusted to pH 8.0 using HCl, followed by the addition of 10 mM $MgCl_2$, 4 mM TCEP (pH 8.0), and 5 μM epimerase and further incubated at 40° C for 16 hr with shaking at 600 rpm. Subsequently, 12 mM ATP (pH 8.0), 3 mM CoASH, 10 mM $MgCl_2$, 12 mM Poa/Paa/D-Pg, 5 mM TCEP and 5 μM PhlA WT or 10 μM PhlA A338G I266A variant were added, and the reaction was incubated at 30°C for 16 h with shaking at 600 rpm. Following this step, the CoA ligase products were processed prior to the final IPNS reaction. Poa and Paa derivatives were extracted with ethyl acetate, whereas for D-Pg the reaction was quenched with an equal volume of methanol to precipitate protein, centrifuged, and the supernatant was evaporated and subsequently subjected to final IPNS reaction. The evaporated reaction mixture was then dissolved in Tris.HCl storage buffer (50 mM Tris.HCl, pH 8.0, 100 mM NaCl, 10% (v/v) glycerol) with 12 mM DTT. After 1 h incubation, 50 U/μL catalase (Sigma) and 10 μM IPNS were added, and the reactions were incubated at 25°C for 2 h. The assays were then quenched by adding 1 volume of acetonitrile (ACN), followed by centrifugation at 14000 × g for 10 min at 4°C. The supernatant was then collected and analysed by high-resolution LC-MS.

Three-enzyme cascade assay: The 3-enzyme cascade assay was initiated with the epimerase enzyme, using a reaction mixture (1 ml total volume) containing 1 mM L-Cys-L-Val, 10 mM $MgCl_2$, 2 mM TCEP (pH 8.0) and 5 μM epimerase in 20 mM Tris.HCl buffer (pH 8.0). The reaction was incubated at 40°C with shaking at 600 rpm for 16 h. The CoA ligase reaction was

then initiated by adding 3 mM ATP (pH 8.0), 1.5 mM CoASH, 10 mM $MgCl_2$, 2 mM Poa/Paa/D-Pg, 2 mM TCEP and either 5 µM PhlA WT or 10 µM PhlA A338G I266A variant. The reaction was then incubated at 30° C for 16 h with shaking at 600 rpm. After completion of the CoA ligase reaction, the reaction mixtures were processed either with ethyl acetate or methanol, then evaporated, and subjected to the final IPNS reaction. The dried extract was reconstituted in Tris.HCl storage buffer (50 mM Tris.HCl, pH 8.0, 100 mM NaCl, 10% (v/v) glycerol) supplemented with 4 mM DTT. Catalase (50 U/µL; Sigma) and 10 µM IPNS were then added, and the mixture was incubated at 25°C for 2 h. The reactions were quenched by the addition of one volume of acetonitrile (ACN), followed by centrifugation at 14,000 g for 10 min at 4°C. The resulting supernatants were collected and analysed by high-resolution LC–MS

Measurement of specific activity of enzymes

The specific activity of TabS was measured using the EnzCheck Inorganic Phosphate Assay Kit (ThermoFisher). Reactions (200 µL) were set up in 96-well UV-transparent plates and contained 10 mM L-Cys or L-SPro, 5 mM L-Val, 5 mM TCEP, 2 mM ATP, 10 mM $MgCl_2$, 0.2 mM MESG (2-amino-6-mercapto-7-methylpurine riboside), 1 U mL^{-1} PNP (purine nucleoside phosphorylase), and 5 µM TabS in 50 mM Tris.HCl buffer (pH 9.0). Reaction mixtures without amino acid substrates were pre-incubated at 30°C for 10 min and initiated by substrate addition. Pi release was monitored at 360 nm at 30°C for 60 min using a Synergy HT plate reader. Reagent concentrations were optimised to avoid rate limitations. Initial rates were determined from Pi calibration curves generated using Pi standards in the absence of TabS. Specific activity is reported as U g^{-1} (1 U is defined as 1 µmol min^{-1} Pi produced under the assay conditions).

The specific activity of Epimerase was determined by measuring the initial activity of the reaction using LC-MS. Reactions (50 µL) containing 1 mM L-Cys-L-Val, 20 mM $MgCl_2$, 2 mM TCEP, and 0.25 µM Epimerase in 20 mM Tris.HCl buffer (pH 8.0) were incubated at 40°C with 500 rpm shaking. Samples were taken at 1-10 mins (2 mins intervals) and quickly quenched by mixing with 50 µL of 1 M HCl, followed by analysis via LC-MS. Initial rates were determined from L-Cys-D-Val calibration curves. Specific activity is reported as U g^{-1} (1 U is defined as 1 µmol min^{-1} L-Cys-D-Val produced under the assay conditions).

The specific activity of PhlA WT enzyme was measured using the EnzCheck Inorganic Pyrophosphate Assay Kit (ThermoFisher). Reactions (200 µL) were set up in 96-well UV-transparent plates and contained Poa (2 mM) or Paa (2 mM), 2 mM CoASH, 3 mM ATP, 5 mM $MgCl_2$, 0.2 mM MESG (2-amino-6-mercapto-7-methylpurine riboside), 1 U mL^{-1} PNP (purine nucleoside phosphorylase), 0.3 U mL^{-1} inorganic pyrophosphatase and 40 nM PhlA in 50 mM Tris.HCl buffer (pH 8.0). Reaction mixtures lacking Poa or Paa were pre-incubated at 30°C for 10 min and initiated by substrate addition. PPi release was monitored at 360 nm at 30°C for 60 min using a Synergy HT plate reader. Reagent concentrations were optimised to avoid rate limitations. Initial rates were determined from PPi calibration curves generated using PPi standards in the absence of PhlA. Specific activity is reported as U g^{-1} (1 U is defined as 1 µmol min^{-1} PPi produced).

As the yield of the PhlA A338G I266A mutant enzyme for D-Pg is low, its specific activity was determined by measuring the initial reaction rate using LC-MS. Reactions (50 µL) containing 1 mM D-Pg, 1 mM L-Cys-D-Val, 3 mM ATP, 1.5 mM CoASH,10 mM $MgCl_2$, 2 mM TCEP, and 10 µM Epimerase in 50 mM Tris.HCl buffer (pH 8.0) were incubated at 30°C with

600 rpm shaking. Samples were taken at 15 min, 30 min, 45 min, 55 min and 80 min intervals and quickly quenched by mixing with 50 µL of 100% methanol, followed by analysis via LC-MS. Initial rates were determined from D-Pg-L-Cys-D-Val calibration curves. Specific activity is reported as U g^{-1} (1 U is defined as 1 µmol min^{-1} D-Pg-L-Cys-D-Val produced under the assay conditions).

The specific activities of the IPNS enzymes (S185R and S185R R87L variants) were determined by measuring the initial activity of the reaction using LC-MS. The solutions (50 µL) of 0.5 mM PaaCV/PoaCV/D-PgCV and 1 mM DTT were pre-incubated in Tris.HCl storage buffer (50 mM Tris.HCl, pH 8.0, 100 mM NaCl, 10% (v/v) glycerol) for 1 h. The reaction was then initiated by the addition of 50U/µL catalase (Sigma) and 0.25 µM IPNS and incubated at 25 °C with 600 rpm shaking. Samples were taken at 0-30 min (PoaCV/PaaCV) or 0-10 mins (PgCV/HpgCV) and quickly quenched by mixing with 50 µL of 100% methanol, followed by analysis via LC-MS. Specific activity is reported as U g^{-1} (1 U is defined as 1 µmol min^{-1} β-lactam variant produced under the assay conditions).

Preparative scale enzymatic reactions

Preparative scale TabS catalysed formation of dipeptides

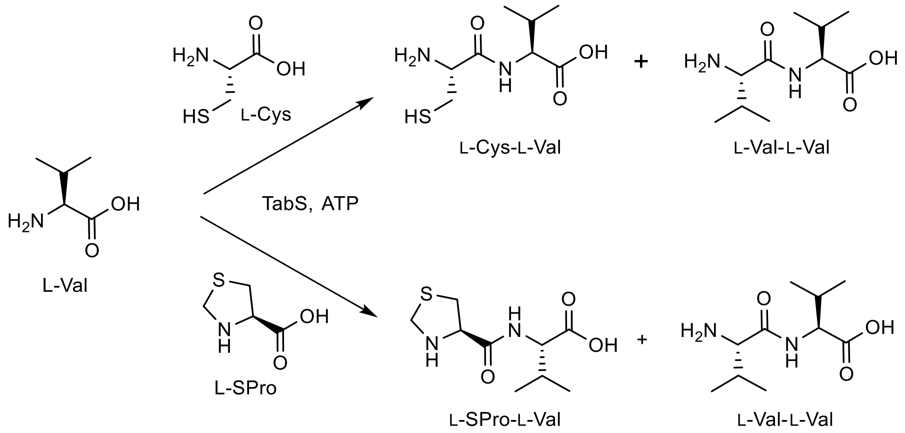

A reaction mixture (50 mL) consisting of L-Val (5 mM, 1.0 equiv), L-Cys/L-SPro (10 mM, 2.0 equiv), ATP (10 mM, pH 9.0), TCEP (2 mM, pH 9.0), $MgCl_2$ (10 mM) and purified TabS (5 µM) were prepared in 50 mM Tris.HCl buffer (pH 9.0) and incubated at 35°C with 200 rpm shaking for 18 h. After incubation, an equal volume of methanol was added to the reaction mixture and centrifuged at 14000 × g for 15 min. The supernatant was then frozen and evaporated. 5 mL of 50% methanol solution was then added, the sample was vortexed thoroughly, and centrifuged at 14,000 g for 10 min. The supernatant was then subjected to preparative HPLC (Luna HILIC 5um 200 Å, LC Column 150 x 10 mm, mobile phase A - 1:1 mixture of 10 mM ammonium formate, pH 3.0 and acetonitrile and mobile phase B - 9:1 mixture of acetonitrile and 10 mM ammonium formate, pH 3.0 (0–2 min, 70% B; 2–14 min, 70–40% B; 14–16 min, 40–10% B; 16-24 min, 10% B; 24-26 min, 10–70% B and finally the column equilibration step 26-40 min, 70% B); flow rate, 5ml/min flow rate) to obtain the dipeptides.

L-Cys-L-Val: **^{1}H NMR** (400 MHz, DMSO-d^6) δ 8.50 (s_{br}, 1H), 4,14(s, 1H), 3.77-3.75 (m, 1H), 3.18- 3.13(m, 1H), 2.95- 2.89 (m, 1H), 2.12- 2.03 (m, 1H), 0.90- 0.88(m, 6H). **^{13}C NMR** (100 MHz, DMSO-d^6) δ 172.2, 166.9, 57.4, 53.5, 29.4, 25.3, 18.9, 17.6.

L-SPro-L-Val: **^{1}H NMR** (400 MHz, DMSO-d^6) δ 7.98 (d, *J* = 8 Hz, 1H), 4.17 (dd, *J* = 8, 4 Hz, 1H), 4.13-4.07(m, 2H), 3.98(t, *J* = 4 Hz, 1H), 2.96-2.93 (m, 2H), 2.11-2.06 (m, 1H), 0.87-0.84 (m, 6H). **^{13}C NMR** (100 MHz, DMSO-d^6) δ 172.7, 170.4, 65.7, 56.8, 53.8, 35.5, 30.1, 19.1, 17.7.

Preparative scale CoA ligase mediated synthesis of N-acyl-L-Cys-D-Val

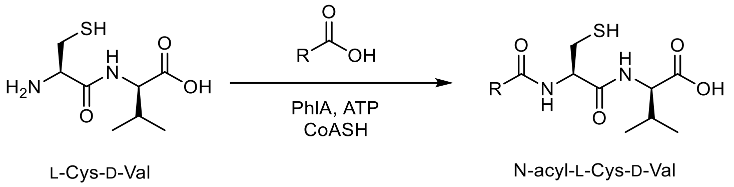

A reaction mixture (10 mL) consisting of Paa/Poa/D-Pg (3 mM, 1.0 equiv, stock solution prepared in Tris.HCl buffer, pH 8.0), L-Cys-D-Val (3 mM, 1.0 equiv; in case for D-PgCV, 1.5 mM L-Cys-D-Val was used to reduce the formation of dimer between D-PgCV & unreacted L-Cys-D-Val), ATP (6 mM, pH 8.0), CoASH (1.5 mM), TCEP (4 mM, pH 8.0), $MgCl_2$ (10mM) and purified CoA ligase PhlA (5 µM) were prepared in 50 mM Tris.HCl buffer (pH 8.0) and incubated at 30°C with 200 rpm shaking for 18 h. After incubation, the reaction mixture was acidified to pH 2 and then extracted with 10 ml of ethyl acetate three times. The organic layers were combined and concentrated *in vacuo* to obtain the crude product. The crude material was then dissolved in 100% methanol solution and subjected to preparative-HPLC. For D-PgCV, the reaction was quenched with equal volume of methanol, centrifuged at 14000 × g for 15 min and the supernatant was then frozen, lyophilised, dissolved in 50% methanol and subjected to preparative-HPLC. Purification was carried out by preparative HPLC (Gemini 5 µm NX-C18 110 Å, LC Column 250 x 10 mm, mobile phase A - water + 0.1% formic acid and mobile phase B - methanol + 0.1% formic acid, 5-95% B gradient over 30 min, 5mL/min flow rate) to obtain the purified product (N-acyl-L-Cys-D-Val).

PaaCV: **^{1}H NMR** (400 MHz, DMSO-d^6) δ 8.43(d, *J* = 8.4 Hz, 1H), 8.11 (d, *J* = 8.6 Hz, 1H), 7.27- 7.19 (m, 5H), 4.74-4.68 (m, 1H), 4.13- 4.10 (m, 1H), 3.12- 3.08 (m, 1H), 2.93- 2.88 (m, 1H), 2.06-1.98 (m, 1H), 0.84- 0.80 (m, 6H). **^{13}C NMR** (100 MHz, DMSO-d^6) δ 170.7, 170.1, 169.4, 136.6, 129.5, 128.6, 126.7, 60.9, 52.4, 42.5, 30.5, 23.2, 19.6, 18.3.

PoaCV: **^{1}H NMR** (400 MHz, DMSO-d^6) δ 8.26 (d, *J* = 8 Hz, 1H), 8.15 (d, *J* = 8 Hz, 1H), 7.33-7.29 (m, 2H), 6.98-6.96 (m, 3H), 4.68-4.63 (m, 1H), 4.58 (s, 2H), 4.21-4.17 (m, 1H), 2.51 (s, 2H), 2.11-2.06 (m, 1H), 0.89-0.87 (m, 6H). **^{13}C NMR** (100 MHz, DMSO-d^6) δ 173.2, 169.9, 168.1, 158.1, 130.0, 121.7, 115.1, 67.1, 57.7, 54.5, 30.5, 27.2, 19.7, 18.3.

D-PgCV: **^{1}H NMR** (500 MHz, DMSO-d^6) δ 7.42 (d, *J* = 5 Hz, 1H), 7.30-7.28 (m, 2H), 7.08-7,03 (m,3H), 4.52-4.48 (m, 2H), 4.08 (s, 1H), 2.07-1.97 (m, 1H), 0.85-0.82 (m, 6H).

Preparative scale enzyme cascade combining epimerase and CoA ligase

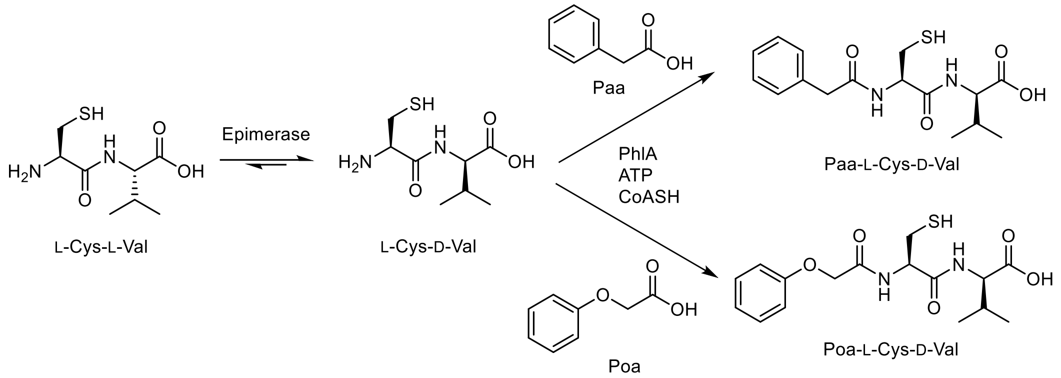

A reaction mixture (40 mL) containing L-Cys-L-Val (5 mM, 1.0 equiv), $MgCl_2$ (20 mM), TCEP (8 mM, pH 8.0), and purified epimerase (5 µM) in 50 mM Tris.HCl buffer (pH 8.0) was incubated at 40°C with shaking at 200 rpm. After 24 hours, Paa or Poa (2.4 mL of 100 mM stock solution in Tris.HCl buffer, pH 8.0, 1.2 equiv), ATP (2.4 mL of 100 mM stock solution in Tris.HCl buffer, pH 8.0 1.2 equiv), additional TCEP (2.4 mL of 100 mM stock solution in Tris.HCl buffer, pH 8.0), CoASH (0.6 mL of 100 mM stock solution in Tris.HCl buffer, pH 8.0, 0.3 equiv), and purified CoA-ligase PhlA (10 µM) were added. The reaction mixture was then incubated at 30°C with 200 rpm shaking for additional 20 hours. The reaction was quenched by acidification to pH 2-3 using 1.0 M HCl. The resulting mixture was extracted with EtOAc (3 × 40 mL). The combined organic layers were washed with brine, dried over $MgSO_4$, and concentrated *in vacuo* to obtain crude product. Purification was carried out by preparative HPLC (Gemini 5 µm NX-C18 110 Å, LC Column 250 x 10 mm, water/MeOH with 0.1% formic acid, 40% isocratic for Paa, 50% isocratic for Poa, 5mL/min flow rate) to obtain the purified product (Paa/Poa-L-Cys-D-Val).

PaaCV: ^{1}H NMR (400 MHz, MeOD) δ 7.45 – 7.15 (m, 5H), 4.59 (t, *J* = 6.4 Hz, 1H), 4.31 (d, *J* = 5.4 Hz, 1H), 3.60 (s, 2H), 2.89 (dd, *J* = 13.7, 6.1 Hz, 1H), 2.80 (dd, *J* = 13.8, 6.8 Hz, 1H), 2.24 – 2.06 (m, 1H), 0.92 (dd, *J* = 19.5, 6.8 Hz, 6H). **^{13}C NMR** (101 MHz, MeOD) δ 174.8, 174.2, 172.0, 136.6, 130.2, 129.7, 128.0, 59.2, 57.0, 43.6, 31.9, 26.9, 19.7, 18.2.
PoaCV: ^{1}H NMR (400 MHz, MeOD) δ 7.34 – 7.27 (m, 2H), 7.04 – 6.96 (m, 3H), 4.73 (t, *J* = 6.1 Hz, 1H), 4.60 (s, 2H), 4.34 (s_{br}, 1H), 3.01 – 2.80 (m, 2H), 2.28 – 2.12 (m, 1H), 0.97 (dd, *J* = 11.3, 6.8 Hz, 6H). **^{13}C NMR** (101 MHz, MeOD) δ 171.7, 171.2, 159.1, 130.7, 122.9, 115.9, 68.1, 59.6, 56.1, 31.9, 27.3, 19.8, 18.3.

Preparative scale synthesis of β-lactams by IPNS

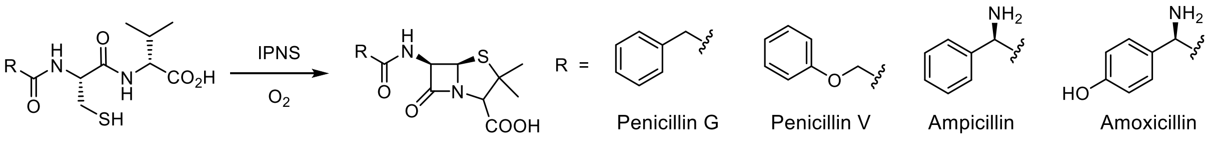

For IPNS assay, a reaction mixture (50 mL) of 5 mM tripeptide (PaaCV /PoaCV/PgCV/HpgCV) was pre-incubated with 7.5 mM of DTT (1.5 equiv) in Tris.HCl storage buffer (50 mM Tris.HCl, pH 8.0, 100 mM NaCl, 10% v/v glycerol) for 1 h to keep the precursor in a reduced state. After incubation, 50U/µL catalase (Sigma) and 10 µM IPNS were added, and the reactions were

incubated at 25°C for 2 h. The assays were then quenched by addition of 1 volume of acetonitrile, centrifuged at 14000 × g for 10 min at 4°C. The supernatant was then collected, kept always at 4°C and subjected to preparative HPLC (Gemini 5 µm NX-C18 110 Å, LC Column 250 x 10 mm, mobile phase A - water + 0.1% formic acid and mobile phase B - methanol + 0.1% formic acid, 5-95% B gradient over 30 min, 5ml/min flow rate) to obtain the β-lactams.

Penicillin G: ^{1}H NMR (400 MHz, DMSO-d^6) δ 8.16 (s, 1H), 7.38-7.27 (m, 5H), 4.46-4.45 (m, 2H), 4.41-4.37 (m, 3H), 1.26 (s, 3H), 1.17 (s, 3H). **^{13}C NMR** (125 MHz, DMSO-d^6) δ 176.8, 176.4, 165.2, 154.3, 142.9, 134.3, 134.0, 132.4, 77.7, 75.7, 68.3, 65.7, 43.7, 32.5.
Penicillin V: ^{1}H NMR (400 MHz, DMSO-d^6) δ 8.39 (d, *J* = 8 Hz, 1H), 7.32-7.28 (m, 2H), 6.99-6.92 (m, 3H), 5.40-5.37 (m, 2H), 4.63(s, 2H), 3.85(s, 1H), 1.51 (s, 3H), 1.46 (s, 3H). **^{13}C NMR** (125 MHz, DMSO-d^6) δ 172.3, 168.3, 167.6, 157.6, 129.5, 121.2, 114.5, 74.2, 66.8, 66.2, 64.6, 57.2, 32.2, 27.4.
Ampicillin: ^{1}H NMR (400 MHz, DMSO-d^6) δ 12.77 (s_{br}, 1H), 8.60 (s, 1H), 7.80 (s, 1H), 7.37-7.30 (m, 5H), 5.09 (s, 1H), 4.95 (s, 1H), 3.83 (s, 1H), 3.58 (s, 1H), 1.56 (s, 3H), 1.22 (s, 3H). **^{13}C NMR** (125 MHz, DMSO-d^6) δ 170.4, 167.0, 166.4, 139.0, 128.3, 127.7, 72.8, 68.8, 60.2, 59.1, 58.6, 26.6.
Amoxicillin: ^{1}H NMR (400 MHz, DMSO-d^6) δ 9.44 (s, 1H), 8.49 (s, 1H), 7.68 (s, 1H), 7.08(d, *J* = 4 Hz, 2H), 6.73 (d, *J* = 8 Hz, 2H), 5.07 (s, 1H), 4.82(s, 1H), 3.79 (s, 1H), 3.57 (s, 1H), 3.51 (s, 1H), 1.55 (s, 3H), 1.21 (s, 3H). **^{13}C NMR** (125 MHz, DMSO-d^6) δ 175.6, 172.6, 171.6, 162.3, 134.6, 134.0, 120.2, 78.11, 73.9, 65.4, 64.3, 63.3, 31.9.

General synthetic route for PoaCV and PaaCV

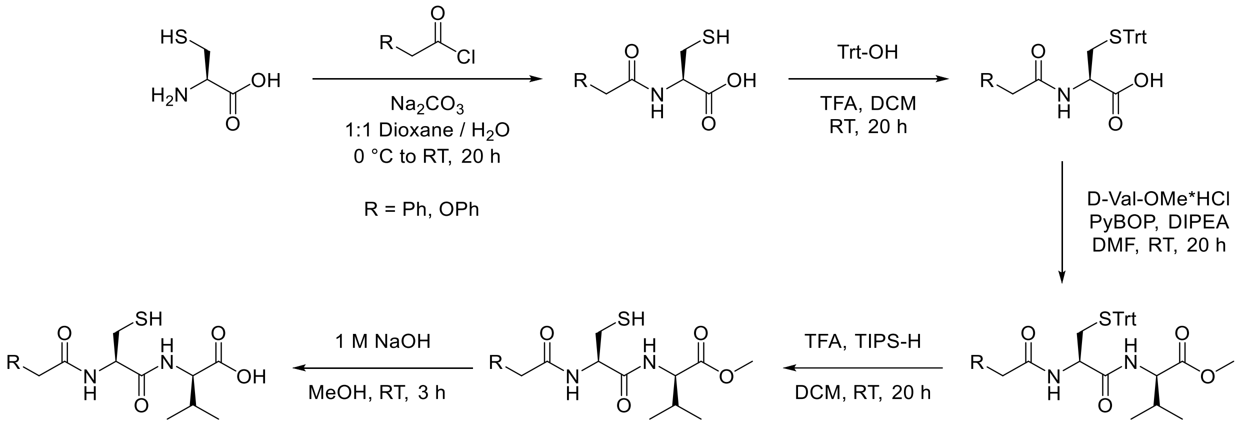

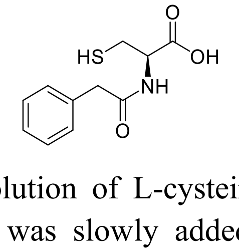

(2-Phenylacetyl)-L-cysteine. To a solution of L-cysteine (1.33 g, 11 mmol, 1.1 equiv) and Na_2CO_3 in degassed water (25 mL) was slowly added a solution of phenylacetyl chloride (1.55 g, 10 mmol, 1 equiv) in degassed 1,4-dioxane (25 mL) at 0°C under nitrogen gas atmosphere and stirring was continued at room temperature for 18 h. The reaction was diluted with water (50 mL), basified with 2 M NaOH (5 mL) and washed with Et_2O (30 mL) and

acidified to pH 1-2 with 6 M HCl. The aqueous layer was extracted with EtOAc (2x50 mL), the combined organic layers were dried over $MgSO_4$ and concentrated under reduced pressure to yield the desired product as a white solid (2.38 g, 9.95 mmol, 99%). **^{1}H NMR** (400 MHz, DMSO) δ 12.86 (s, 1H), 8.42 (d, *J* = 7.9 Hz, 1H), 7.31 – 7.18 (m, 5H), 4.40 (td, *J* = 7.5, 4.6 Hz, 1H), 3.52 (s, 2H), 2.91 – 2.72 (m, 2H), 2.38 (t, *J* = 8.5 Hz, 1H). **^{13}C NMR** (101 MHz, DMSO) δ 171.64, 170.29, 136.23, 129.08, 128.19, 126.36, 54.42, 41.89, 25.65. **HR-MS:** calc. for $[M+H]^+$ $C_{11}H_{14}NO_3S$ 240.0689, found 240.0698 (Δppm = 3.8 ppm).

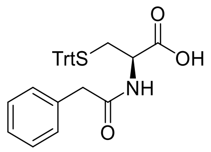

***N*-(2-phenylacetyl)-*S*-trityl-L-cysteine.** To a solution of (2-phenylacetyl)-L-cysteine (1.2 g, 5 mmol, 1 equiv) and triphenylmethanol (1.37 g, 5.25 mmol, 1.05 equiv) in dry DCM (10 mL), trifluoroacetic acid (1.27 mL, 19.25 mmol, 3.85 equiv) was dropwise added at 0°C under nitrogen gas atmosphere and stirring was continued at room temperature for 18 h. The reaction was diluted with DCM (40 mL) and washed with brine (30 mL). The aqueous layer was extracted with DCM (2x40 mL), the combined organic layers were dried over $MgSO_4$ and concentrated under reduced pressure. Silica gel column chromatography (elute: DCM / MeOH) afforded the desired product as a white amorphous solid (2.17 g, 4.51 mmol, 90%). **^{1}H NMR** (400 MHz, DMSO) δ 8.54 (d, *J* = 8.1 Hz, 1H), 7.36 – 7.18 (m, 20H), 4.24 (td, *J* = 8.4, 4.9 Hz, 1H), 3.55 – 3.46 (m, 2H), 2.56 (dd, *J* = 11.9, 8.5 Hz, 1H), 2.43 (dd, *J* = 12.1, 5.0 Hz, 1H). **^{13}C NMR** (101 MHz, DMSO) δ 171.74, 170.14, 144.24, 136.23, 129.11, 128.15, 128.11, 126.85, 126.36, 66.12, 51.32, 41.93, 33.30. **HR-MS:** calc. for $[M+Na]^+$ $C_{30}H_{27}NO_3SNa$ 504.1604, found : 504.1606 (Δppm = 0.45 ppm).

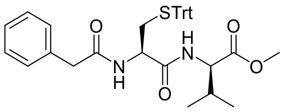

Methyl *N*-(2-phenylacetyl)-*S*-trityl-L-cysteinyl-D-valinate. To a solution of *N*-(2-phenylacetyl)-*S*-trityl-L-cysteine (963 mg, 2 mmol, 1 equiv), L-valine methyl ester hydrochloride (402 mg, 2.4 mmol, 1.2 equiv) and PyBOP (1.14 g, 2.2 mmol, 1.1 equiv) in DMF (10 mL) was added DIPEA (1.05 mL, 6 mmol, 3 equiv) and the reaction was stirred for 20 h at room temperature. The reaction was diluted with DCM (75 mL) and successively washed with sat. $NaHCO_3$ solution (30 mL), 1 M HCl (30 mL) and brine (30 mL). The organic layer was dried over $MgSO_4$ and concentrated under reduced pressure. Silica gel column chromatography (elute: n-hexane / EtOAc) afforded the desired product as a white amorphous solid (900 mg, 1.51 mmol, 75%). **^{1}H NMR** (400 MHz, DMSO) δ 8.38 (d, *J* = 8.5 Hz, 1H), 8.33 (d, *J* = 8.5 Hz, 1H), 7.33 – 7.22 (m, 20H), 4.66 – 4.57 (m, 1H), 4.15 (dd, *J* = 8.5, 6.3 Hz, 1H), 3.60 (s, 3H), 3.56 – 3.45 (m, 2H), 2.34 – 2.21 (m, 2H), 1.98 (h, *J* = 6.7 Hz, 1H), 0.77 (d, *J* = 6.8 Hz, 6H). **^{13}C NMR** (101 MHz, DMSO) δ 171.71, 170.07, 169.95, 144.18, 136.29, 129.06, 129.02, 128.10, 128.02, 126.69, 126.28, 65.59, 57.20, 51.74, 50.89, 41.88, 34.86, 30.00, 18.92, 17.88. **HR-MS:** calc. for $[M+Na]^+$ $C_{36}H_{38}N_2O_4SNa$ = 617.2445, found 617.2452 (Δppm = 1.26 ppm).

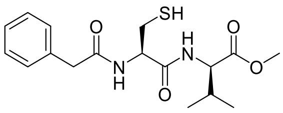

Methyl (2-phenylacetyl)-L-cysteinyl-D-valinate. To a solution of methyl *N*-(2-phenylacetyl)-*S*-trityl-L-cysteinyl-D-valinate (475 mg, 0.8 mmol, 1 equiv) and triisopropylsilane (819 µL, 4 mmol, 5 equiv) in DCM (8 mL), trifluoroacetic acid (260 µL, 4 mmol, 5 equiv) was dropwise added at 0°C and stirring was continued at room temperature for 18 h. The reaction was diluted with DCM (50 mL) and washed with sat. $NaHCO_3$ solution (20 mL). The aqueous layer was extracted with DCM (30 mL) and the combined organic layers were dried over $MgSO_4$ and concentrated under reduced pressure. Silica gel column chromatography (elute: n-hexane / EtOAc) afforded the desired product as a white amorphous solid (263 mg, 0.75 mmol, 93%). **^{1}H NMR** (400 MHz, DMSO) δ 8.34 (d, *J* = 8.5 Hz, 1H), 8.30 (d, *J* = 8.3 Hz, 1H), 7.32 – 7.16 (m, 5H), 4.56 (q, *J* = 7.4 Hz, 1H), 4.22 (dd, *J* = 8.5, 6.3 Hz, 1H), 3.63 (s, 3H), 3.51 (t, *J* = 2.5 Hz, 2H), 2.85 – 2.59 (m, 2H), 2.17 (s, 1H), 2.03 (q, *J* = 6.7 Hz, 1H), 0.85 (d, *J* = 6.8 Hz, 6H). **^{13}C NMR** (101 MHz, DMSO) δ 171.81, 170.21, 169.95, 136.28, 129.07, 128.16, 126.32, 57.28, 54.52, 51.76, 41.99, 30.08, 26.62, 19.00, 18.09. **HR-MS:** calc. for $[M+H]^+$ $C_{17}H_{25}N_2O_4S$ 353.1530, found 353.1539 (Δppm = 2.69 ppm).

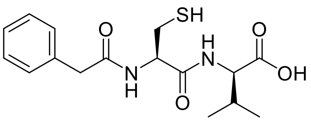

(2-Phenylacetyl)-L-cysteinyl-D-valine. To a solution of methyl (2-phenylacetyl)-L-cysteinyl-D-valinate (221 mg, 0.6 mmol, 1 equiv) in degassed MeOH (18 mL) was added 1 M NaOH solution (1.8 mL) under nitrogen gas atmosphere and the reaction was stirred for 3 h at room temperature. The organic solvent was removed under reduced pressure and the residue was taken up with EtOAc (30 mL) and 1 M HCl (30 mL). The aqueous layer was extracted with EtOAc (30 mL) and the combined organic layers were dried over $MgSO_4$ and concentrated under reduced pressure. The isolated product was suspended with Et_2O (10 mL), filtered, and dried *in vacuo* to afford the desired product as a white solid (180 mg, 0.51 mmol, 85%). **^{1}H NMR** (400 MHz, DMSO) δ 12.70 (s, 1H), 8.29 (d, *J* = 8.3 Hz, 1H), 8.16 (d, *J* = 8.7 Hz, 1H), 7.40 – 7.10 (m, 5H), 4.56 (td, *J* = 7.7, 5.5 Hz, 1H), 4.18 (dd, *J* = 8.7, 5.6 Hz, 1H), 3.51 (d, *J* = 2.7 Hz, 2H), 2.84 – 2.60 (m, 2H), 2.16 (t, *J* = 8.2 Hz, 1H), 2.04 (p, *J* = 6.5 Hz, 1H), 0.85 (t, *J* = 6.9 Hz, 6H). **^{13}C NMR** (101 MHz, DMSO) δ 172.77, 170.22, 169.82, 136.29, 129.08, 128.18, 126.32, 57.06, 54.60, 42.02, 30.05, 26.73, 19.18, 17.85. **HR-MS:** calc. for $[M+Na]^+$ $C_{16}H_{22}N_2O_4SNa$ 361.1192, found 361.1207 (Δppm = 4.29 ppm).

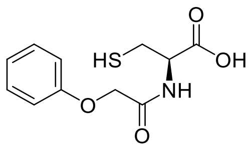

(2-phenoxyacetyl)-L-cysteine. To a solution of L-cysteine (1.33 g, 11 mmol, 1.1 equiv) and Na_2CO_3 in degassed water (25 mL) was slowly added a solution of phenoxyacetyl chloride (1.71 g, 10 mmol, 1 equiv) in degassed 1,4-dioxane (25 mL) at 0°C under nitrogen gas atmosphere and stirring was continued at room temperature for 18 h. The reaction was diluted with water (50 mL), basified with 2 M NaOH (5 mL) and washed with Et_2O (30 mL) and acidified to pH 1-2 with 6 M HCl. The precipitate was filtered, washed with water, and dried *in*

vacuo to yield the desired product as a white solid (1.76 g, 6.89 mmol, 99%). **^{1}H NMR** (400 MHz, DMSO) δ 13.02 (s, 1H), 8.30 (d, *J* = 7.9 Hz, 1H), 7.30 (t, *J* = 7.9 Hz, 2H), 6.98 (d, *J* = 7.8 Hz, 3H), 4.58 (s, 2H), 4.49 (td, *J* = 7.6, 4.5 Hz, 1H), 3.00 – 2.78 (m, 2H), 2.39 (t, *J* = 8.5 Hz, 1H). **^{13}C NMR** (101 MHz, DMSO) δ 171.37, 167.93, 157.70, 129.52, 121.26, 114.78, 66.67, 54.07, 25.39. **HR-MS:** calc. for $[M+H]^+$ $C_{11}H_{14}NO_4S$ 255.0565, found 256.0648 (Δppm = 3.9 ppm).

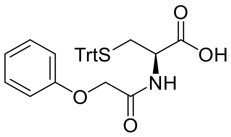

***N*-(2-phenoxyacetyl)-*S*-trityl-L-cysteine.** To a solution of (2-phenoxyacetyl)-L-cysteine (765 mg, 3 mmol, 1 equiv) and triphenylmethanol (820 mg, 3.15 mmol, 1.05 equiv) in dry DCM (6 mL), trifluoroacetic acid (394 µL, 6 mmol, 3.85 equiv) was dropwise added at 0°C under nitrogen gas atmosphere and stirring was continued at room temperature for 18 h. The reaction was diluted with DCM (30 mL) and washed with brine (20 mL). The aqueous layer was extracted with DCM (2x30 mL), the combined organic layers were dried over $MgSO_4$ and concentrated under reduced pressure. Silica gel column chromatography (elute: DCM / MeOH) afforded the desired product as a white amorphous solid (1.25 g, 2.52 mmol, 84%). **^{1}H NMR** (400 MHz, DMSO) δ 12.24 (s, 1H), 7.74 (d, *J* = 8.2 Hz, 1H), 6.66 – 6.52 (m, 17H), 6.27 (dd, *J* = 16.8, 7.9 Hz, 3H), 3.62 (td, *J* = 8.6, 4.8 Hz, 1H), 2.08 – 1.81 (m, 2H), 1.81 – 1.77 (m, 1H). **^{13}C NMR** (101 MHz, DMSO) δ 171.43, 167.77, 157.73, 144.25, 129.47, 129.13, 128.10, 126.86, 121.24, 114.81, 66.67, 66.24, 51.02, 33.00. **HR-MS:** calc. for $[M+Na]^+$ $C_{30}H_{27}NO_4SNa$ 520.1553, found 520.1564 (Δppm = 2.21 ppm).

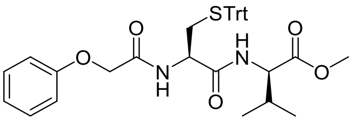

Methyl *N*-(2-phenoxyacetyl)-*S*-trityl-L-cysteinyl-D-valinate. To a solution of *N*-(2-phenoxyacetyl)-*S*-trityl-L-cysteine (479 mg, 1 mmol, 1 equiv), L-valine methyl ester hydrochloride (201 mg, 1.2 mmol, 1.2 equiv) and PyBOP (572 mg, 1.1 mmol, 1.1 equiv) in DMF (5 mL) was added DIPEA (523 µL, 3 mmol, 3 equiv) and the reaction was stirred for 20 h at room temperature. The reaction was diluted with DCM (50 mL) and successively washed with sat. $NaHCO_3$ solution (20 mL), 1 M HCl (20 mL) and brine (20 mL). The organic layer was dried over $MgSO_4$ and concentrated under reduced pressure. Silica gel column chromatography (elute: n-hexane / EtOAc) afforded the desired product as a white amorphous solid (516 mg, 0.84 mmol, 84%). **^{1}H NMR** (400 MHz, DMSO) δ 8.42 (d, *J* = 8.5 Hz, 1H), 8.18 (d, *J* = 8.6 Hz, 1H), 7.34 – 7.23 (m, 17H), 6.98 – 6.91 (m, 3H), 4.67 (q, *J* = 7.2 Hz, 1H), 4.56 (s, 2H), 4.19 – 4.11 (m, 1H), 3.66 – 3.56 (m, 3H), 2.39 – 2.28 (m, 2H), 2.05 – 1.95 (m, 1H), 0.79 (d, *J* = 6.9 Hz, 6H). **^{13}C NMR** (101 MHz, DMSO) δ 171.70, 169.74, 167.40, 157.68, 144.15, 129.50, 129.03, 128.07, 126.78, 121.20, 114.66, 66.52, 65.72, 57.30, 51.80, 50.52, 34.84, 30.02, 18.96, 17.94. **HR-MS:** calc. for $[M+Na]^+$ $C_{36}H_{38}N_2O_5SNa$ 633.2394, found 633.2411 (Δppm = 2.84 ppm).

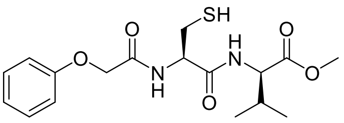

Methyl (2-phenoxyacetyl)-L-cysteinyl-D-valinate. To a solution of methyl *N*-(2-phenoxyacetyl)-*S*-trityl-L-cysteinyl-D-valinate (427 mg, 0.7 mmol, 1 equiv) and triisopropylsilane (756 µL, 3.5 mmol, 5 equiv) in DCM (7 mL), trifluoroacetic acid (230 µL, 3.5 mmol, 5 equiv) was dropwise added at 0°C and stirring was continued at room temperature for 18 h. The reaction was diluted with DCM (50 mL) and washed with sat. $NaHCO_3$ solution (20 mL). The aqueous layer was extracted with DCM (30 mL) and the combined organic layers were dried over $MgSO_4$ and concentrated under reduced pressure. Silica gel column chromatography (elute: n-hexane / EtOAc) afforded the desired product as a white amorphous solid (248 mg, 0.67 mmol, 96%). **^{1}H NMR** (400 MHz, DMSO) δ 8.44 (d, *J* = 8.4 Hz, 1H), 8.14 (d, *J* = 8.3 Hz, 1H), 7.34 – 7.25 (m, 2H), 7.02 – 6.91 (m, 3H), 4.69 – 4.60 (m, 1H), 4.57 (s, 2H), 4.22 (dd, *J* = 8.4, 6.3 Hz, 1H), 3.64 (s, 3H), 2.89 – 2.75 (m, 2H), 2.19 (d, *J* = 7.9 Hz, 1H), 2.10 – 2.00 (m, 1H), 0.87 (dd, *J* = 6.8, 2.1 Hz, 6H). **^{13}C NMR** (101 MHz, DMSO) δ 171.81, 169.63, 167.67, 157.67, 129.51, 121.21, 114.69, 66.63, 57.37, 53.98, 51.80, 30.05, 26.65, 19.02, 18.14. **HR-MS:** calc. for $[M+Na]^+$ $C_{17}H_{24}N_2O_5SNa$ 391.1298, found 391.1309 (Δppm = 2.95 ppm).

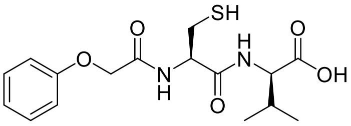

(2-Phenoxyacetyl)-L-cysteinyl-D-valine. To a solution of methyl (2-phenoxyacetyl)-L-cysteinyl-D-valinate (238 mg, 0.645 mmol, 1 equiv) in degassed MeOH (12 mL) was added 1 M NaOH solution (1.94 mL) under nitrogen gas atmosphere and the reaction was stirred for 3 h at room temperature. The solvent was removed under reduced pressure and the residue was taken up with EtOAc (30 mL) and 1 M HCl (30 mL). The aqueous layer was extracted with EtOAc (30 mL) and the combined organic layers were dried over $MgSO_4$ and concentrated under reduced pressure to afford the desired product a white solid (220 mg, 0.62 mmol, 96%). **^{1}H NMR** (400 MHz, DMSO) δ 12.71 (s, 1H), 8.28 (d, *J* = 8.7 Hz, 1H), 8.14 (d, *J* = 8.3 Hz, 1H), 7.30 (t, *J* = 7.8 Hz, 2H), 7.05 – 6.87 (m, 3H), 4.62 (d, *J* = 40.7 Hz, 3H), 4.20 (dd, *J* = 8.7, 5.7 Hz, 1H), 2.81 (td, *J* = 13.9, 6.4 Hz, 2H), 2.27 – 1.95 (m, 2H), 0.88 (dd, *J* = 6.9, 4.2 Hz, 6H). **^{13}C NMR** (101 MHz, DMSO) δ 172.79, 169.55, 167.72, 157.71, 129.56, 121.27, 114.74, 66.71, 57.20, 54.07, 30.06, 26.80, 19.24, 17.92. **HR-MS:** calc. for $[M+H]^+$ $C_{16}H_{23}N_2O_5S$ 355.1322, found 355.1338 (Δppm = 4.46 ppm).

Enzyme Sequences

TabS:

Amino acid sequence:

```
MGSSHHHHHHSSGLVPRGSHMTQAKENILVVVDGYSSGSQLPTLMAESGWKCVHVSSSANPPEYYLRTYHKDEYIAHFEYQGDIQS
LASAVEAWHPAAVLPGTESGVIVADLLAAALQLPGNDPSTSLARRDKYTMHESLKAVGLRSMDHFLAVDRDALSAWAERGSWPVVI
KPQASAGTDSVTFCADQGELLESFDQLFGTVNQLGERNNAVLAQRLLVGPEYFINGVSGHGKHLITEIWRADKLPAPDGGWIYDRA
VLFDPTSPEMQEIVRYVHGVLDALGIRYGANHTELIVTADGPTLIECASRLSGGLHRPAANYAVGASQLDLVGKLVREGESAIDDI
LQTWQPHRYALWQVQFISNQEGVVARSSYDELLKTLKSNAWLQRAPKEGDTVVKTVDLFSSPGIVFMSHADGNVLHDDYRTVREWE
RTSRLFSVQ*
```

Coding sequence:

```
ATGGGCAGCAGCCATCATCATCATCATCACAGCAGCGGCCTGGTGCCGCGCGGCAGCCATATGACTCAGGCTAAAGAAAATATTCT
TGTCGTGGTAGATGGGTATTCCTCGGGCAGTCAGCTTCCCACGTTGATGGCGGAAAGTGGGTGGAAGTGTGTTCATGTGTCATCAT
CAGCGAACCCGCCGGAATATTACTTGCGCACCTACCACAAGGATGAATACATTGCCCATTTTGAGTATCAGGGCGATATTCAAAGT
CTGGCCTCCGCCGTAGAAGCGTGGCATCCAGCGGCTGTACTGCCTGGGACCGAGTCCGGGGTGATCGTTGCCGACCTGCTGGCGGC
CGCCTTACAGCTGCCGGGTAACGACCCGTCCACCAGTCTGGCTCGCCGCGATAAATATACGATGCATGAATCATTAAAAGCGGTGG
GTTTGCGCTCCATGGACCATTTCCTGGCTGTTGATCGTGATGCATTGTCGGCTTGGGCAGAACGTGGCTCATGGCCGGTGGTAATT
AAGCCTCAGGCGTCGGCGGGCACAGATTCCGTTACCTTCTGTGCGGATCAAGGTGAACTGCTTGAAAGCTTTGATCAGTTATTCGG
CACGGTTAACCAGCTTGGCGAACGTAACAACGCGGTTTTGGCGCAACGTTTACTGGTCGGGCCAGAGTATTTTATCAATGGTGTTT
CTGGCCACGGCAAACACCTGATCACCGAAATTTGGCGTGCCGATAAACTTCCCGCCCCGGATGGCGGTTGGATTTACGATCGCGCG
GTTTTGTTCGACCCGACTTCTCCCGAAATGCAGGAAATCGTGCGCTATGTTCATGGCGTGTTGGATGCCTTGGGAATCCGCTACGG
TGCCAACCATACGGAGTTAATTGTGACGGCCGACGGTCCGACTCTCATTGAATGCGCCTCCCGCCTGTCAGGGGGTCTGCATCGTC
CTGCGGCCAACTACGCAGTCGGCGCGTCGCAACTTGACCTGGTTGGTAAACTGGTTCGTGAGGGTGAATCAGCTATTGATGATATT
TTGCAGACCTGGCAACCCCATCGCTATGCCCTGTGGCAGGTCCAGTTTATTAGCAACCAAGAGGGCGTCGTTGCACGTTCCAGCTA
TGACGAGTTACTGAAAACATTGAAAAGCAATGCGTGGCTTCAGCGTGCACCCAAAGAGGGCGATACGGTCGTGAAGACGGTGGACC
TGTTTAGCTCGCCAGGAATCGTGTTTATGTCCCACGCAGATGGCAACGTCCTGCATGACGACTATCGCACGGTACGCGAATGGGAA
CGCACCAGTCGTCTGTTCTCTGTGCAATGA
```

Epimerase:

Amino acid sequence:

```
MGSSHHHHHHSSGLVPRGSHMSRIVNVKLSLKRYEYEKPFHITGSVSSESRNVEVEIVLESGVKGYGEASPSFRVNGERVEALLAI
ENAVREMITGIDVRNYARIFEITDRLFGFPSLKAAVQFATLDALSQELGTQVCYLLGGKRDEIETDKTVGIDTVENRVKEAKKIFE
EGFRVIKIKVGENLKEDIEAVEEIAKVTRGAKYIVDANMGYTQKEAVEFARAVYQKGIDIAVYEQPVRREDIEGLKFVRFHSPFPV
AADESARTKFDVMRLVKEEAVDYVNIKLMKSGISDALAIVEIAESSGLKLMIGCMGESSLGINQSVHFALGTGAFEFHDLDSHLML
KEEVFRGKFIQDGPRMRVKDQ*
```

Coding sequence:

```
ATGGGCAGCAGCCATCATCATCATCATCACAGCAGCGGCCTGGTGCCGCGCGGCAGCCATATGTCGAGGATCGTGAACGTGAAGCT
TTCTCTCAAGAGATACGAATACGAAAAGCCGTTTCACATAACTGGAAGCGTATCTTCCGAAAGCAGAAATGTCGAGGTTGAAATCG
TTTTGGAAAGTGGTGTCAAAGGGTACGGAGAAGCGTCTCCCTCCTTCAGAGTGAACGGTGAAAGGGTGGAAGCACTTCTTGCAATA
GAAAACGCTGTGAGGGAGATGATCACAGGCATCGATGTGCGAAACTATGCCAGGATCTTCGAGATCACAGACAGGCTCTTCGGGTT
TCCGAGTTTGAAAGCAGCCGTTCAATTTGCCACACTGGACGCTCTCTCTCAAGAACTTGGAACACAGGTCTGTTATCTCCTTGGAG
GAAAAAGAGATGAGATAGAAACGGACAAAACAGTTGGGATAGACACCGTCGAAAACCGTGTGAAGGAGGCAAAGAAGATATTCGAA
GAGGGTTTCAGAGTGATCAAGATAAAAGTTGGAGAGAATTTGAAGGAAGACATCGAAGCCGTGGAAGAGATAGCGAAAGTCACCCG
TGGAGCGAAGTACATAGTTGACGCGAACATGGGATACACCCAGAAGGAGGCGGTGGAGTTCGCGAGAGCAGTATATCAAAAAGGAA
TAGACATCGCTGTGTACGAACAACCTGTGAGGAGGGAAGACATAGAAGGCTTGAAGTTCGTGAGGTTCCACTCTCCGTTTCCCGTC
GCGGCGGACGAATCCGCGAGGACGAAGTTCGATGTGATGAGGCTTGTGAAGGAAGAAGCGGTTGATTACGTGAACATAAAACTCAT
GAAGTCGGGAATTTCCGACGCTCTTGCCATAGTGGAAATAGCAGAATCGTCAGGTTTGAAGCTCATGATCGGATGCATGGGAGAGT
CCTCGCTCGGAATAAATCAGAGTGTTCATTTTGCGCTTGGAACGGGAGCTTTTGAGTTTCATGACCTCGACAGTCACCTGATGCTG
AAGGAAGAAGTTTTCAGAGGAAAATTCATCCAGGATGGTCCGAGAATGAGGGTGAAAGATCAATGA
```

PhlA:

Amino acid sequence:

```
MVFLPPKESGQLDPIPDNIPISEFMLNERYGRVRHASSRDPYTCGITGKSYSSKEVANRVDSLARSLSKEFGWAPNEGSEWDKTLA
VFALNTIDSLPLFWAVHRLGGVLTPANASYSAAELTHQLLDSKAKALVTCVPLLSISLEAAAKAGLPKNRIYLLDVPEQLLGGVKP
PAGYKSVSELTQAGKSLPPVDELRWSAGEGARRTAFVCYSSGTSGLPKGVMISHRNVIANTLQIKAFEQNYRDGGGTKPASTEVAL
GLLPQSHIYALVVIGHAGAYRGDQTIVLPKFELKSYLNAIQQYKISALFLVPPIIIHMLGTQDVCSKYDLSSVTSLFTGAAPLGME
TAADFLKLYPNILIRQGYGLTETCTVVSSTHPHDIWLGSSGALLPGVEARIVTPENKEITTYDSPGELVVRSPSVVLGYLNNEKAT
AETFVDGWMRTGDEAVIRRSPKGIEHVFIVDRIKELIKVKGLQVAPAELEAHILAHPDVSDCAVIAIPDDRAGEVPKAIVVKSASA
GSDESVSQALVKYVEDHKARHKWLKGGIRFVDAIPKSPSGKILRRLIRDQEKEARRKAGSKILEHHHHHH*
```

Coding sequence:

```
ATGGTCTTCCTGCCGCCGAAAGAATCTGGACAGCTGGATCCGATCCCGGATAACATCCCGATTTCAGAATTCATGCTTAACGAACG
TTACGGCCGTGTCCGCCATGCATCGTCTCGCGATCCCTATACATGCGGGATCACGGGTAAAAGCTATAGCTCAAAGGAAGTTGCAA
ACCGTGTGGATTCTTTAGCACGCAGCCTGTCCAAAGAGTTCGGCTGGGCACCAAACGAGGGCAGCGAGTGGGACAAAACCTTAGCG
GTTTTCGCTTTGAATACCATTGACTCGCTGCCTTTATTTTGGGCGGTGCATCGTCTTGGTGGCGTCCTGACCCCAGCGAATGCTTC
CTATTCGGCGGCAGAATTGACCCACCAACTCCTGGACAGCAAAGCTAAAGCATTAGTTACGTGCGTGCCGCTGCTTAGCATTAGTC
TCGAGGCCGCCGCAAAGGCCGGACTGCCTAAGAATCGTATTTATCTGTTGGACGTCCCAGAACAACTGCTGGGTGGTGTGAAACCA
```

CCGGCTGGCTATAAATCAGTGTCAGAGTTAACGCAAGCCGGCAAATCGCTGCCCCCGGTCGATGAGCTGCGTTGGAGTGCCGGCGA
GGGCGCGCGTCGTACCGCCTTCGTTTGTTATAGTAGCGGTACTTCAGGCCTTCCCAAGGGCGTTATGATTTCGCATCGTAATGTTA
TTGCAAACACTCTGCAAATTAAAGCCTTCGAACAAAATTATCGTGACGGCGGCGGGACCAAACCGGCAAGCACCGAAGTAGCGCTG
GGCCTGTTGCCTCAATCCCACATTTACGCACTGGTTGTTATCGGTCACGCCGGTGCCTATCGCGGAGATCAGACCATTGTGCTGCC
GAAGTTTGAGTTAAAGTCGTATTTAAATGCGATTCAGCAATATAAGATTTCAGCATTGTTTCTGGTTCCGCCTATTATTATCCATA
TGTTAGGTACCCAGGATGTCTGTAGCAAATACGATCTCAGCAGCGTCACCTCGCTGTTTACCGGCGCAGCCCCTCTTGGGATGGAA
ACCGCGGCGGACTTTCTGAAGCTGTATCCTAATATCCTCATTCGTCAGGGCTATGGCCTTACCGAAACCTGTACCGTAGTCTCAAG
CACGCATCCACATGACATTTGGCTTGGAAGTTCAGGAGCACTTCTTCCGGGCGTGGAAGCCCGTATCGTTACCCCGGAGAATAAAG
AGATTACCACATATGATAGCCCAGGGGAGTTAGTTGTTCGCTCTCCGTCAGTAGTTTTAGGGTACCTGAATAATGAGAAGGCGACT
GCGGAAACCTTCGTTGATGGGTGGATGCGCACCGGTGATGAAGCGGTTATTCGCCGCTCACCCAAAGGAATTGAACATGTTTTCAT
CGTGGACCGTATTAAAGAACTGATTAAAGTGAAAGGGCTCCAGGTGGCTCCGGCTGAGCTGGAAGCGCACATTCTGGCACATCCAG
ACGTGAGCGATTGCGCAGTGATTGCGATTCCTGACGACCGCGCGGGCGAGGTTCCGAAAGCGATCGTCGTCAAAAGCGCATCGGCC
GGTAGCGATGAGTCAGTGAGCCAAGCCCTGGTTAAATACGTGGAAGATCATAAAGCGCGCCATAAATGGCTGAAAGGCGGCATTCG
TTTCGTTGACGCAATCCCTAAATCCCCATCAGGCAAAATCCTGCGCCGTTTAATTCGCGATCAGGAAAAGGAAGCTCGCCGTAAAG
CGGGCTCGAAAATTCTCGAGCACCACCACCACCACCACTGA

scIPNS (S185R):

Amino acid sequence:
MGSSHHHHHHSSGLVPRGSHMPVLMPSAHVPTIDISPLFGTDAAAKKRVAEEIHGACRGSGFFYATNHGVDVQQLQDVVNEFHGAM
TDQEKHDLAIHAYNPDNPHVRNGYYKAVPGRKAVESFCYLNPDFGEDHPMIAAGTPMHEVNLWPDEERHPRFRPFCEGYYRQMLKL
STVLM**R**GLALALGRPEHFFDAALAEQDSLSSVRLIRYPYLEEYPPVKTGPDGQLLSFEDHLDVSMITVLFQTQVQNLQVETVDGWR
DIPTSENDFLVNCGTYMAHVTNDYFPAPNHRVKFVNAERLSLPFFLNGGHEAVIEPFVPEGASEEVRNEALSYGDYLQHGLRALIV
KNGQT*

Coding sequence:
ATGGGCAGCAGCCATCATCATCATCATCACAGCAGCGGCCTGGTGCCGCGCGGCAGCCATATGCCAGTTTTGATGCCCAGTGCTCA
CGTACCCACGATTGACATCTCACCTCTTTTCGGGACGGACGCGGCAGCAAAGAAACGTGTTGCGGAAGAGATCCATGGCGCGTGTC
GCGGGAGTGGGTTCTTCTATGCGACAAACCATGGAGTTGACGTTCAACAACTCCAAGATGTAGTTAACGAGTTTCACGGTGCAATG
ACGGACCAAGAGAAACATGATCTCGCAATCCACGCGTATAACCCTGATAATCCCCACGTCCGTAACGGTTATTATAAGGCGGTTCC
CGGCCGTAAAGCGGTTGAAAGCTTTTGTTATCTCAATCCGGACTTTGGGGAAGACCACCCTATGATCGCTGCGGGTACTCCCATGC
ATGAGGTAAACTTGTGGCCCGACGAAGAACGTCATCCACGCTTCCGCCCGTTTTGTGAAGGTTACTATCGTCAGATGTTAAAATTG
TCAACCGTATTAATGCGTGGGTTAGCCTTAGCGTTAGGCCGCCCTGAGCACTTCTTCGACGCGGCGCTTGCAGAACAAGACTCATT
AAGCTCAGTT**cgt**TTGATTCGTTACCCCTACTTAGAAGAGTACCCACCCGTAAAGACGGGACCGGACGGTCAACTTTTATCCTTCG
AAGATCACCTTGACGTTTCGATGATTACTGTGCTTTTCCAAACGCAAGTTCAAAATTTACAGGTGGAAACGGTTGACGGCTGGCGC
GACATTCCTACATCGGAAAATGACTTTCTCGTTAATTGTGGTACGTACATGGCCCATGTTACGAATGATTATTTCCCAGCCCCTAA
TCACCGTGTGAAATTCGTAAATGCAGAACGCTTATCGTTACCCTTCTTCTTAAATGGTGGACACGAAGCGGTAATCGAACCATTTG
TTCCCGAGGGTGCGTCTGAGGAAGTCCGTAACGAAGCCTTATCGTACGGGGACTACCTTCAACATGGCTTACGCGCGTTGATTGTC
AAGAATGGACAAACATAA

caIPNS (S185R):

Amino acid sequence:
MGSSHHHHHHSSGLVPRGSHMPIPMPSAHVPTIDISPLSGSDADAKSRVAREIHDACRGSGFFYASHHGVDVQLLQDVVNEFHRTM
TDQEKYDLAIHAYNKANPHIRNGYYMAVKGKKAVESFCYLNPAFTDDHPMIKAGTPLHEVNRWPDEERHPRFRSFCESYYRQMLRL
STVIM**R**GLALALGKPEHFFDAALAEADTLSSVRLIRYPYLEDYPPVKTGPDGTKLSFEDHLDVSMITVLFQTEVQNLQVETVDGWR
DLPTSGEDFLVNCGTYLGHVTNDYFPAPNHRVKFVNAERLSLPFFLNAGHHAVIDPFVPEGAAEPVKNAPLPYGEYLQHGLRALIV
KNGQT*

Coding sequence:
ATGGGCAGCAGCCATCATCATCATCATCACAGCAGCGGCCTGGTGCCGCGCGGCAGCCATATGCCAATCCCAATGCCTTCTGCTCA
CGTACCCACTATCGACATCTCGCCTCTCTCTGGTAGTGACGCCGACGCAAAGTCTCGTGTCGCACGTGAGATCCACGACGCCTGTC
GTGGATCGGGGTTCTTCTACGCTTCCCACCACGGTGTAGACGTCCAATTACTTCAAGACGTTGTTAATGAGTTCCACCGTACTATG
ACGGACCAAGAGAAGTACGACCTTGCCATCCACGCTTACAATAAGGCAAATCCACACATCCGTAATGGATACTACATGGCAGTCAA
GGGAAAGAAGGCCGTCGAGTCGTTCTGTTACCTTAATCCAGCCTTCACGGACGACCACCCTATGATCAAGGCCGGAACTCCTCTTC
ACGAGGTTAATCGTTGGCCAGACGAGGAGCGTCACCCACGTTTCCGTTCGTTCTGTGAGTCCTACTACCGTCAAATGCTTCGTCTT
TCCACGGTAATCATGCGTGGTCTCGCTCTCGCACTTGGTAAGCCAGAGCACTTCTTCGACGCCGCATTAGCCGAGGCCGACACGTT
ATCATCCGTA**cgt**TTGATCCGTTACCCTTACTTAGAGGACTACCCACCAGTAAAGACGGGACCCGACGGTACGAAGTTAAGTTTCG
AGGACCACCTTGACGTATCGATGATCACAGTATTATTCCAAACTGAGGTTCAAAATCTTCAAGTTGAGACGGTTGACGGTTGGCGT
GACTTACCTACTTCAGGAGAGGACTTCTTGGTTAATTGTGGTACGTACTTGGGACACGTTACGAATGACTACTTCCCAGCACCCAA
TCACCGTGTCAAGTTCGTCAATGCCGAGCGTTTATCTCTTCCTTTCTTCCTTAATGCTGGGCACCACGCAGTTATCGACCCTTTCG
TACCTGAGGGAGCCGCAGAGCCTGTCAAGAATGCCCCTCTTCCTTACGGGGAGTACCTGCAACACGGTTTGCGTGCACTTATCGTA
AAGAATGGACAAACGTAA

Primers used for PhlA mutation

Mutation		Primer sequence (5'→3')
A338G	Forward	GTTTACCGGCGGCGCCCCTCTTGGGAT
	Reverse	AGCGAGGTGACGCTGCTG
I266A	Forward	TCAATCCCACGCGTACGCACTGGTTGTTATCG
	Reverse	GGCAACAGGCCCAGC
T365A	Forward	CTATGGCCTTGCGGAAACCTGTACCGT
	Reverse	CCCTGACGAATGAGGATATTAGGATACAGC
T369S	Forward	GAAACCTGTAGCGTAGTCTCAAGCACGCATC
	Reverse	GGTAAGGCCATAGCCCTGACGAATG
T369A	Forward	GAAACCTGTGCGGTAGTCTCAAGCACGCATC
	Reverse	GGTAAGGCCATAGCCCTGACGAATG
A338G I266A F335K	Forward	CACCTCGCTGAAAACCGGCGGCGCCCCTCTTG
	Reverse	ACGCTGCTGAGATCGTATTTGCTACAGACATCCTGG
A338G I266A Y267F	Forward	CAATCCCACGCGTTCGCACTGGTTGTT
	Reverse	AGGCAACAGGCCCAGC
A338G I266A V270N	Forward	CCCACGCGTACGCACTGAACGTTATCGGTCACGCCGGTGCCTAT
	Reverse	CGATAACGTTCAGTGCGTACGCGTGGGATTGAGGCAACAGGCCC
A338G I266A V271S	Forward	CACGCGTACGCACTGGTTAGCATCGGTCACGCCGGT
	Reverse	GGATTGAGGCAACAGGCCCA
A338G I266A T369P	Forward	GAAACCTGTCCGGTAGTCTCAAGCACGCATC
	Reverse	GGTAAGGCCATAGCCCTGACGAATG
A338G I266A S373M	Forward	GAAACCTGTACCGTAGTCTCAATGACG CAT C
	Reverse	GGTAAGGCCATAGCCCTGACGAATG
A338G I266A V270A V271A	Forward	CACGCGTACGCACTGGCGGCGATCGGTCACGCCGGT
	Reverse	GGATTGAGGCAACAGGCCCA
A338G I266A V270A V271S	Forward	CACGCGTACGCACTGGCGAGCATCGGTCACGCCGGT
	Reverse	GGATTGAGGCAACAGGCCCA
A338G I266A V270N V271A	Forward	CACGCGTACGCACTGAACGCGATCGGTCACGCCGGT
	Reverse	GGATTGAGGCAACAGGCCCA
A338G I266A V270N V271S	Forward	CACGCGTACGCACTGAACAGCATCGGTCACGCCGGT
	Reverse	GGATTGAGGCAACAGGCCCA
A338G I266A T369P S373M	Forward	GAAACCTGTCCGGTAGTCTCAATGACGCATC
	Reverse	GGTAAGGCCATAGCCCTGACGAATG

Supplementary Tables & Figures

PhlA mutant	% conversion	PhlA mutant	% conversion
1. WT	0	13. A338G I266A T365S	9.39±0.25
2. I266A	0	14. A338G I266A T369S	10.44±0.21
3. A338G	2.23±0.01	15. A338G I266A V270A	12.77±0.22
4. T365A	0	16. A338G I266A V271A	14.10±0.20
5. T369S	1.05±0.10	17. A338G I266A V270N	12.33±0.22
6. T369A	0	18. A338G I266A V271S	5.76±0.28
7. A338G I266A	26.62±0.02	19. A338G I266A T369P	0.94±0.02
8. A338G T365A	0	20. A338G I266A S373M	12.97±0.24
9. A338G T369A	1.72±0.05	21. A338G I266A V270A V271A	4.54±0.16
10. A338G T369S	1.62±0.02	22. A338G I266A V270A V271S	1.88±0.02
11. A338G I266A F335K	0	23. A338G I266A V270N V271A	2.53±0.09
12. A338G I266A Y267F	14.46±0.16	24. A338G I266A V270N V271S	0.85±0.02
		25. A338G I266A T369P S373M	3.73±0.10

Supplementary Table 1. % conversion for D-Pg-L-Cys-D-Val formed by PhlA mutants with D-Pg and L-Cys-D-Val substrates. (data are represented as mean values of three independent experiments and error bars represent standard error, n = 3).

Source of homologue[1]	Protein production[2] (mg/L)
S. tirandamycinicus	0.5
S. clavuligerus	1.5
S. jumonjinensis	2.5
S. griseus[3]	3.5
S. cattleya	4.0

[1] *S. = Streptomyces*
[2] Amount of soluble protein purified under standard conditions
[3]The mutation in this homologue is T185R

Supplementary Table 2. Production levels of S185R mutants of IPNS homologues from several Streptomyces species.

Enzymes	Substrate	Specific activity (mU/mg)
TabS	L-SPro + L-Val	10.52 ± 0.21
	L-Cys + L-Val	14.24 ± 0.21
Epimerase	L-Cys-L-Val	343.8 ± 12.8
PhlA	Paa + CoASH	718.9 ± 32.4
	Poa + CoASH	3236.6 ± 93.7
PhlA A338G I266A	D-Pg + CoASH + L-Cys-D-Val	40.81 ± 0.68
IPNS S185R	PaaCV	110.82 ±3.12
	PoaCV	11.58 ± 2.40
	PgCV	33.34 ± 0.76
	HpgCV	35.67 ± 1.52
IPNS S185R R87L	PaaCV	73.91 ± 2.58
	PoaCV	17.08 ± 3.24
	PgCV	122.85 ± 4.82
	HpgCV	52.76 ± 3.66

Supplementary Table 3. Specific activity of the enzymes used in the penicillin biosynthetic pathway. Specific activity (mU/mg) = initial rate (μM/min) / enzyme conc. (g/L) where 1 Unit (U) is the amount of enzyme activity that produces 1 μmol of product per minute under defined assay conditions. (data are represented as mean values of three independent experiments and error bars represent standard error, n = 3)

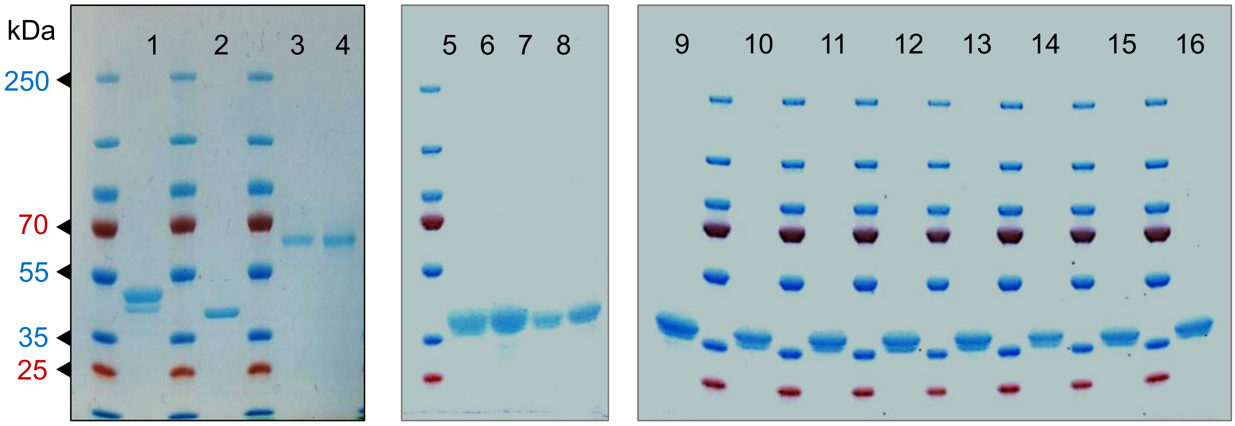

Supplementary Figure 1. SDS PAGE of the purified proteins: Lane **1**: TabS (48.3 kDa), **2**: Epimerase (40.8 kDa), **3-4**: PhlA WT and A338G I266A (63.7 kDa), **5**: scIPNS wt (39.2 kDa), **6**: scIPNS S185R (39.2 kDa), **7**: caIPNS wt (39.2 kDa), **8**: caIPNS S185R (39.2 kDa) and, **9**: caIPNS S185R R87A, **10**: caIPNS S185R R87L, **11**: caIPNS S185R R87S, **12**: caIPNS S185R S183A, **13**: caIPNS S185R S183M **14**: caIPNS S185R E210A, **15**: caIPNS S185R E210G, **16**: caIPNS S185R N285G. Ladder: PageRuler™ Plus Prestained Protein Ladder (10-250 kDa)

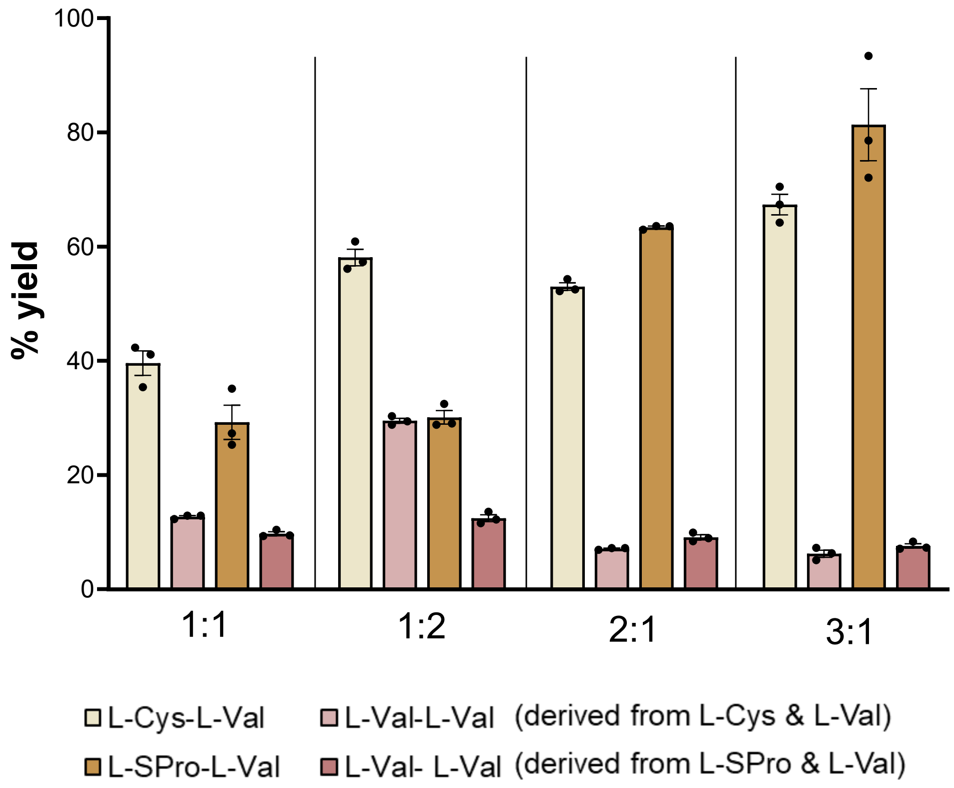

Supplementary Figure 2. Percent (%) yield of L-Cys-L-Val or L-SPro-L-Val and L-Val-L-Val dipeptide produced from the TabS-catalysed reaction using varying molar ratios of L-Cys/L-SPro to L-Val (1:1, 1:2, 2:1, and 3:1). [data are represented as mean values of 3 independent experiments and error bars represent standard error, n = 3; Source data are provided as a Source Data file]

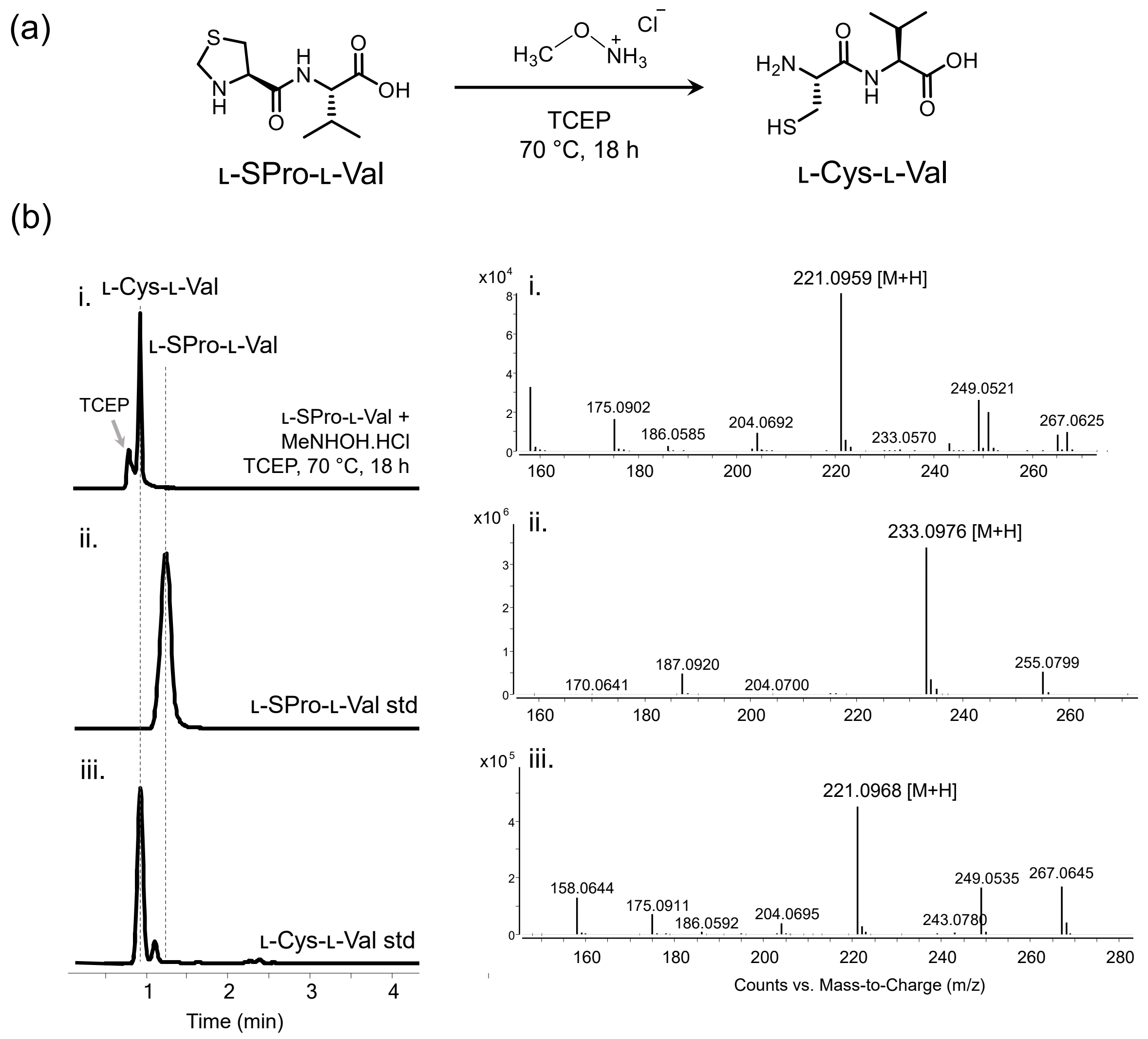

Supplementary Figure 3. (a) Conversion of L-SPro-L-Val into L-Cys-L-Val using methoxyamine hydrochloride ($MeONH_2$.HCl). (b) EIC and mass spectra of the (i) reaction mixture containing L-SPro-L-Val dipeptide, methoxyamine hydrochloride and TCEP showing formation of L-Cys-L-Val (m/z calcd. for $[M+H]^+$: 221.0954, observed 233.0959; Δppm = 2.3), (ii) L-SPro-L-Val synthetic standard (m/z calcd. for $[M+H]^+$: 233.0954, observed 233.0976; Δppm = 9.4) and (iii) the L-Cys-L-Val synthetic standard (m/z calcd. for $[M+H]^+$: 221.0954, observed 221.0968; Δppm = 6.3).

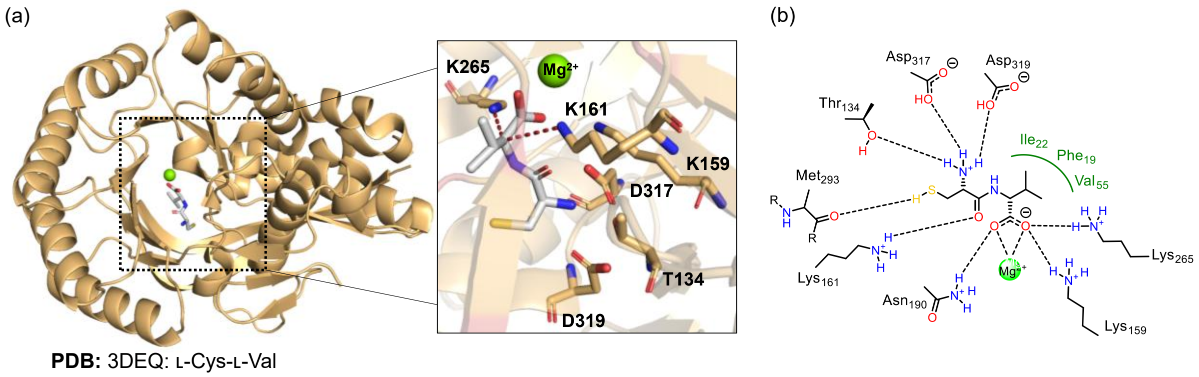

Supplementary Figure 4. (a) X-ray structure of *T. maritima* epimerase (PDB 3DEQ) with L-Cys-L-Val docked in the active site, with an expansion showing the putative substrate binding pocket and catalytic amino acid residues. **(b)** Schematic diagram showing putative amino acid residues contacting the L-Cys-L-Val substrate. Asp317 & Asp319, belonging to the epimerase DxD motif, and a Thr134 residue interact with α-ammonium group of the dipeptide and the Cys thiol group of the dipeptide make a H-bond contact with a backbone carbonyl group (Met293), which may help position the C-terminal (epimerised) residue of the dipeptide substrate in the active side pocket. The hydrophobic binding pocket formed by hydrophobic residues including Phe19, Ile22 and Val55, is likely to accommodate the L-Val side chain.

(a)

Biochemically characterized CoA ligases from the genome of P. chrysogenum

gene name	gene number	Activity
phlA	Pc22g14900	Phenylacetate-CoA ligase
phlB	Pc22g202700	Adipyl and fatty acyl-CoA ligase
phlC	Pc13g12270	Aryl (coumaroyl, phenylacetyl)-CoA ligase
ary4	Pc21g20650	Aminoacyl, phenylacetyl-CoA ligase

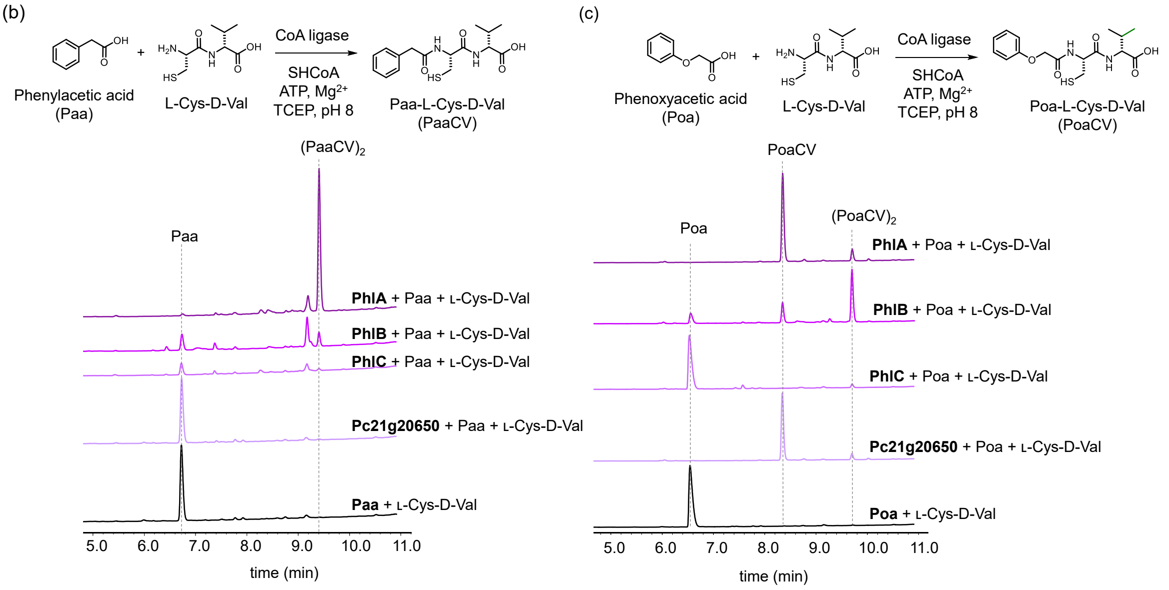

Supplementary Figure 5. (a) Biochemically characterised CoA ligases found in the genome of *P. chrysogenum*. (b-c) HPLC analysis of the *in vitro* enzymatic assay of *P. chrysogenum* CoA ligases with phenylacetic acid (Paa) or phenoxyacetic acid (Poa) and L-Cys-D-Val to produce PaaCV or PoaCV.

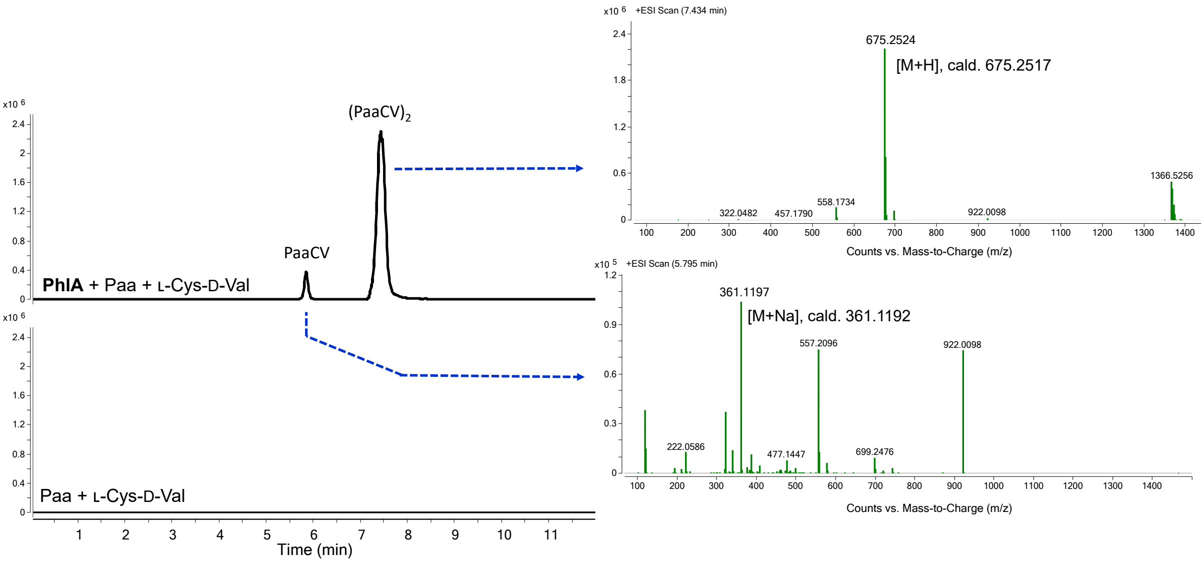

Supplementary Figure 6. LC-MS (EIC) traces and mass spectrum of the enzymatic conversion to PaaCV by PhlA CoA ligase from phenylacetic acid (Paa) and L-Cys-D-Val. (PaaCV)$_2$ (m/z calculated for $[M+H]^+$: 675.2517, observed 675.2524; Δppm = 1.04) and PaaCV (m/z calculated for $[M+H]^+$: 361.1192, observed 361.1197; Δppm = 1.4)

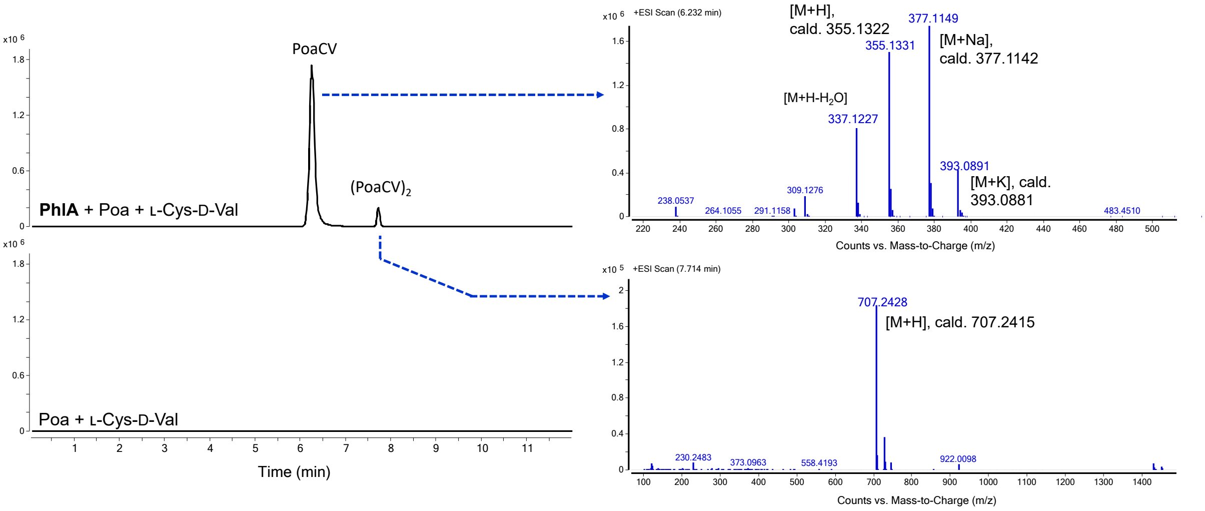

Supplementary Figure 7. LC-MS (EIC) traces and mass spectrum of the enzymatic conversion to PoaCV by PhlA CoA ligase from phenoxyacetic acid (Poa) and L-Cys-D-Val. PoaCV (m/z calculated for $[M+H]^+$: 355.1322, observed 355.1331 (Δppm = 2.5); $[M+Na]^+$: 377.1142, observed 377.1149 (Δppm = 1.85) & for (PoaCV)$_2$, m/z calcd. $[M+H]^+$: 707.2415, observed 707.2428 (Δppm = 1.84)

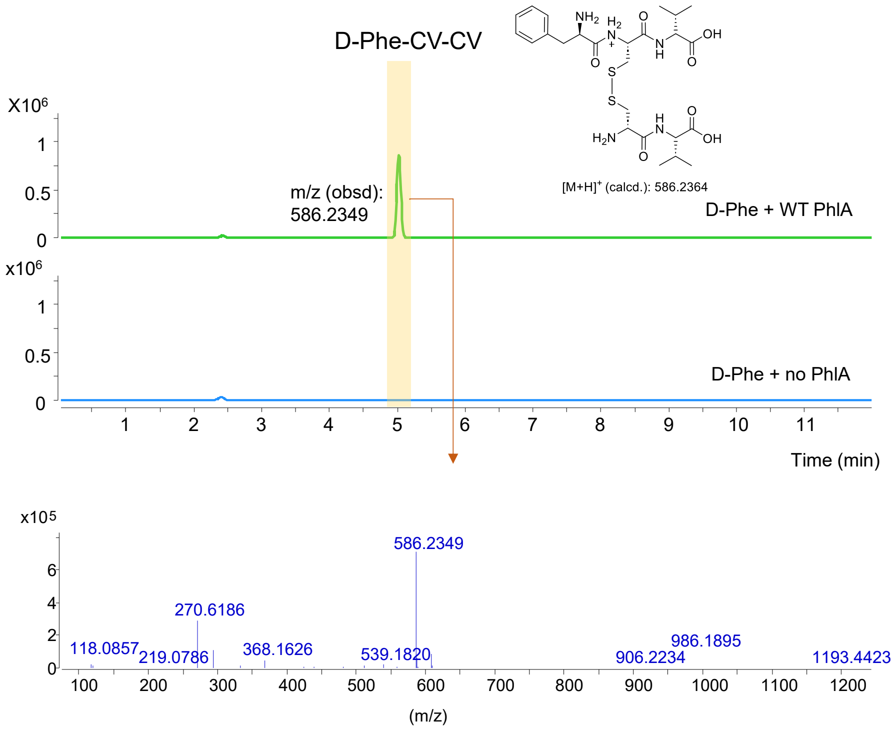

Supplementary Figure 8. LC-MS (EIC) traces and mass spectrum of the enzymatic conversion of D-Phenylalanine (D-Phe) and L-Cys-D-Val to D-PheCV catalysed by PhlA CoA ligase. Dimer of D-PheCV and CV, m/z calcd. $[M+H]^+$: 586.2364, observed 586.2349 (Δppm = 2.6)

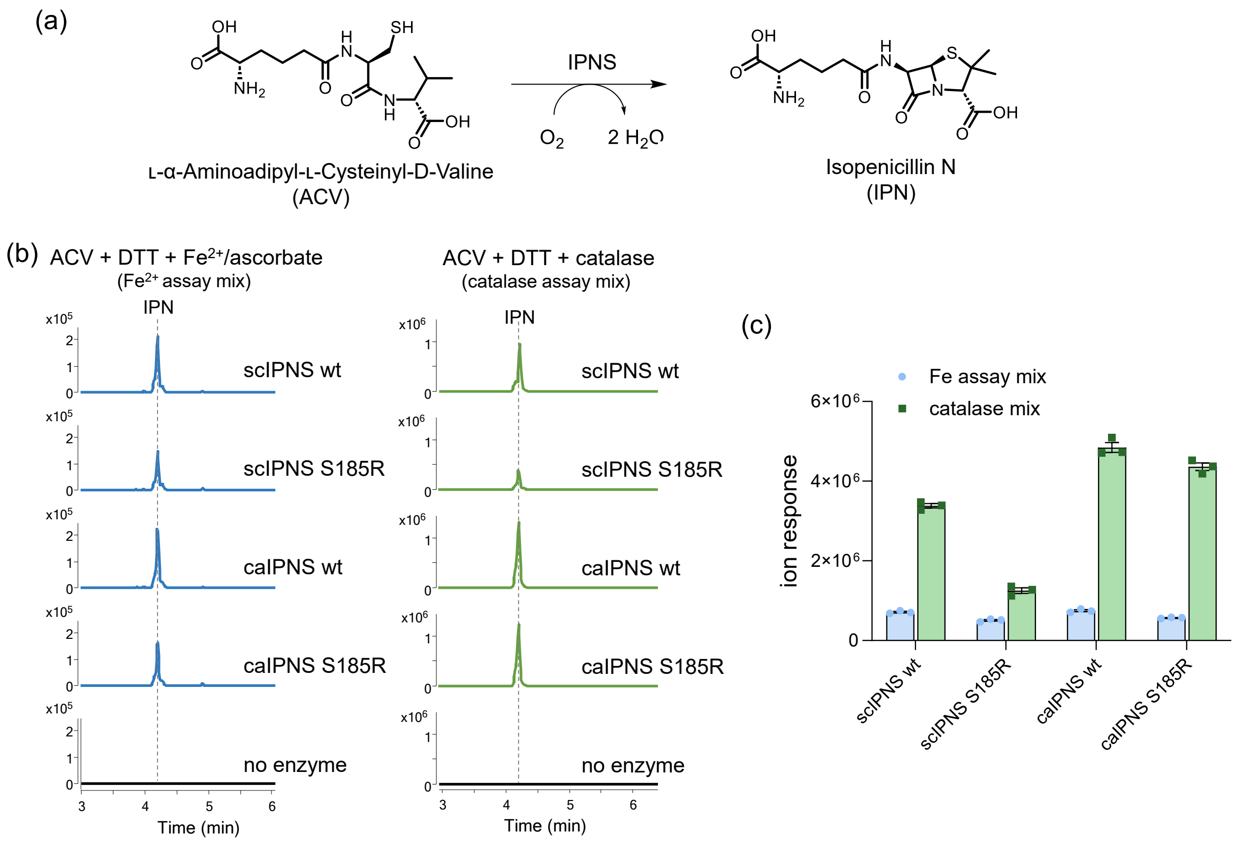

Supplementary Figure 9. (a) Isopenicillin N (IPN) formation from ACV by IPNS enzyme. (b) LC-MS (EIC) traces for the IPN formation from ACV tripeptide by wild type (wt) and S185R mutant of scIPNS and caIPNS in Fe^{2+}/ascorbate or catalase assay mix. (c) Bar chat showing ion responses for IPN formed by wt and S185R IPNS mutants in Fe^{2+}/ascorbate or catalase assay mix (data are represented as mean values of 3 independent experiments and error bars represent standard error, n = 3).

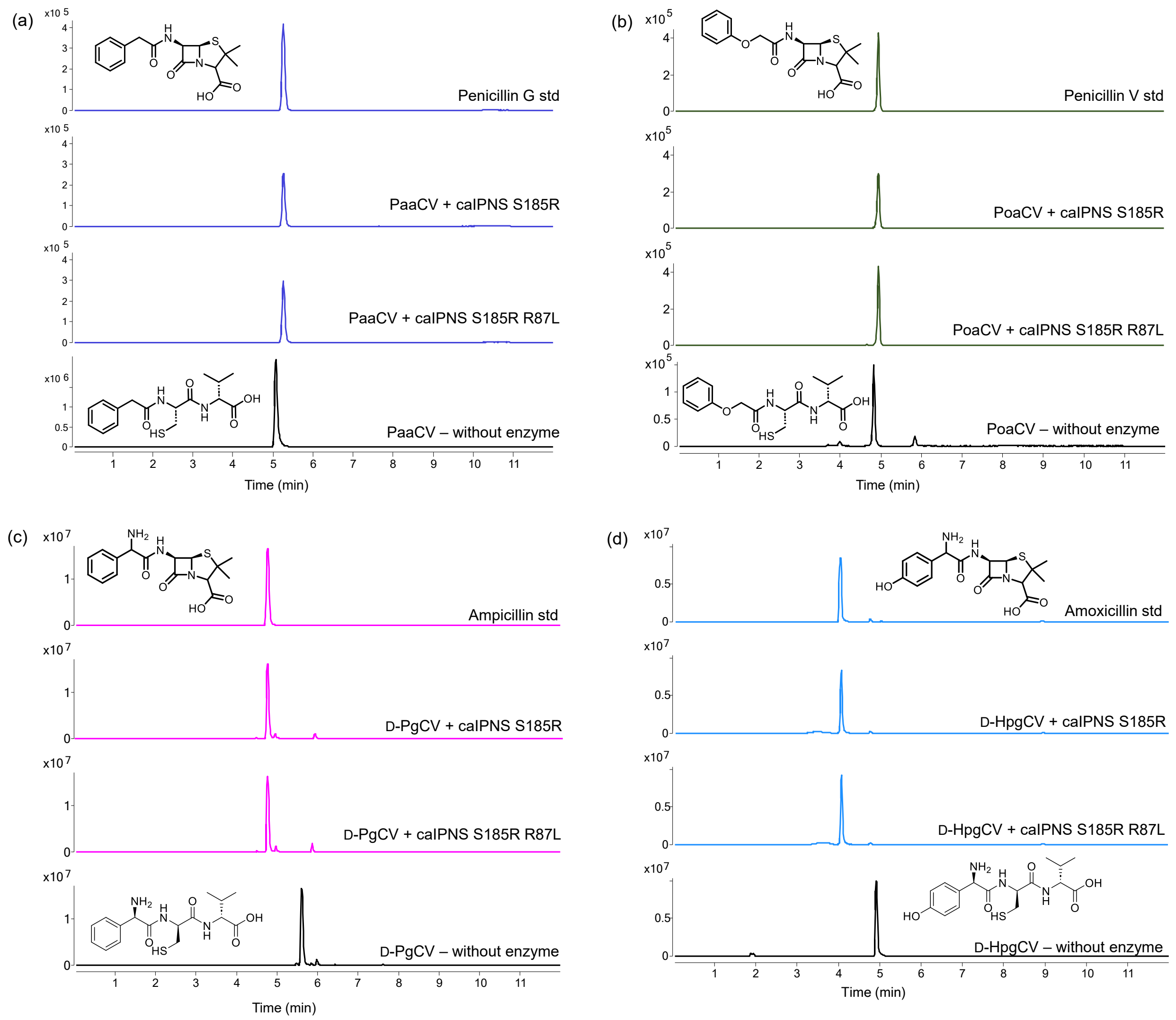

Supplementary Figure 10. EIC traces of (a) Penicillin G, (b) Penicillin V, (c) Ampicillin and (d) Amoxicillin produced from PaaCV, PoaCV, PgCV and HpgCV, respectively by caIPNS S185R and S185R R87L mutants in catalase assay mix.

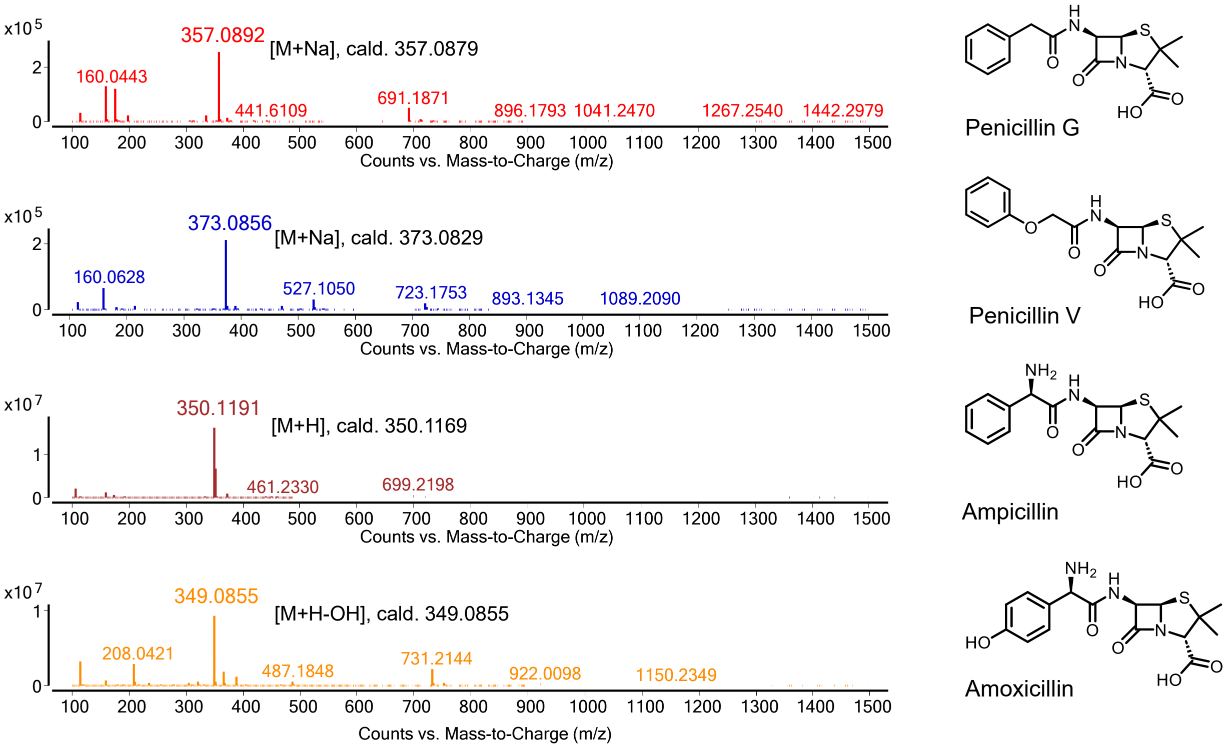

Supplementary Figure 11. MS spectrum of Penicillin G, V, Ampicillin and Amoxicillin formed in the *in vitro* assay from PaaCV/PoaCV/PgCV/HpgCV by caIPNS S185R R87L. Penicillin G m/z calcd. $[M+H]^+$: 357.0879, observed 357.0892 (Δppm = 3.6), Penicillin V m/z calcd. $[M+H]^+$: 373.0829, observed 373.0856 ((Δppm = 7.2), Ampicillin m/z calcd. $[M+H]^+$: 350.1169, observed 350.1191 ((Δppm = 6.3), Amoxicillin m/z calcd. $[M+H]^+$: 349.0855, observed 349.0855 ((Δppm = 0).

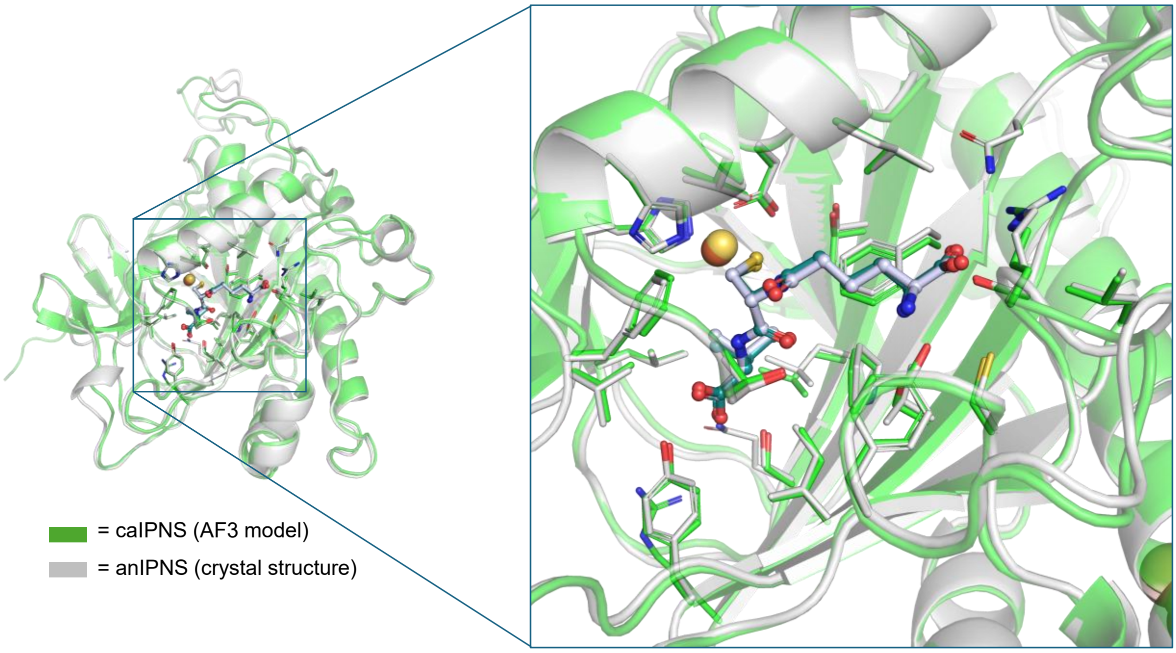

Supplementary Figure 12. AlphaFold3 model of caIPNS superimposed with crystal structure of anIPNS (PDB: 1BK0)

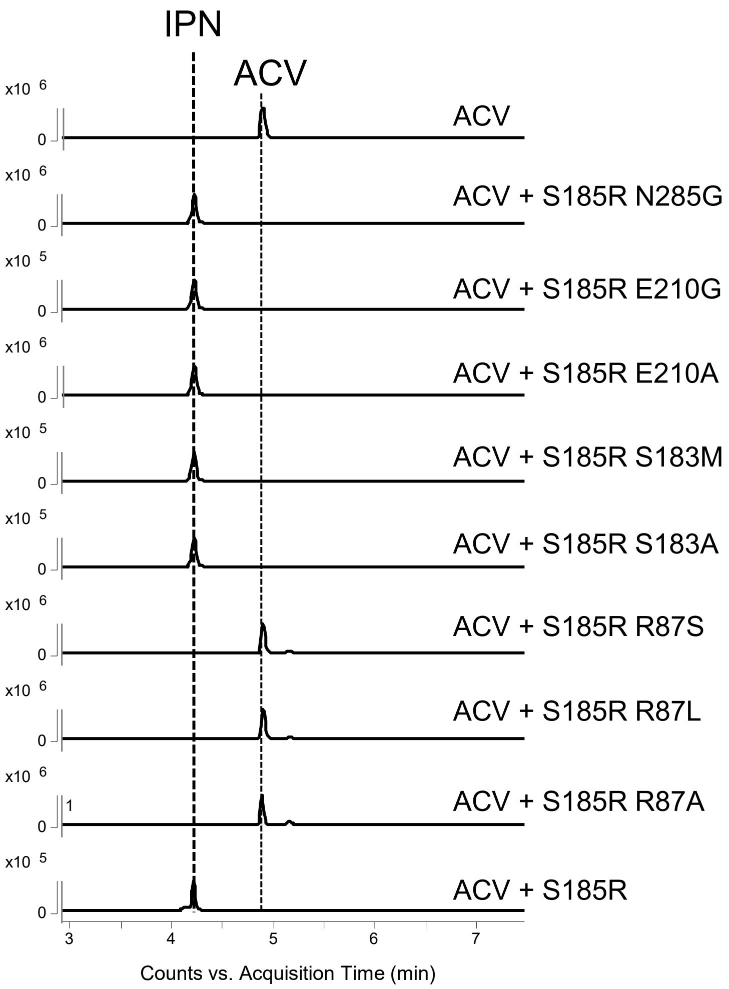

Supplementary Figure 13. *In vitro* enzymatic assay with ACV native tripeptide with caIPNS mutants.

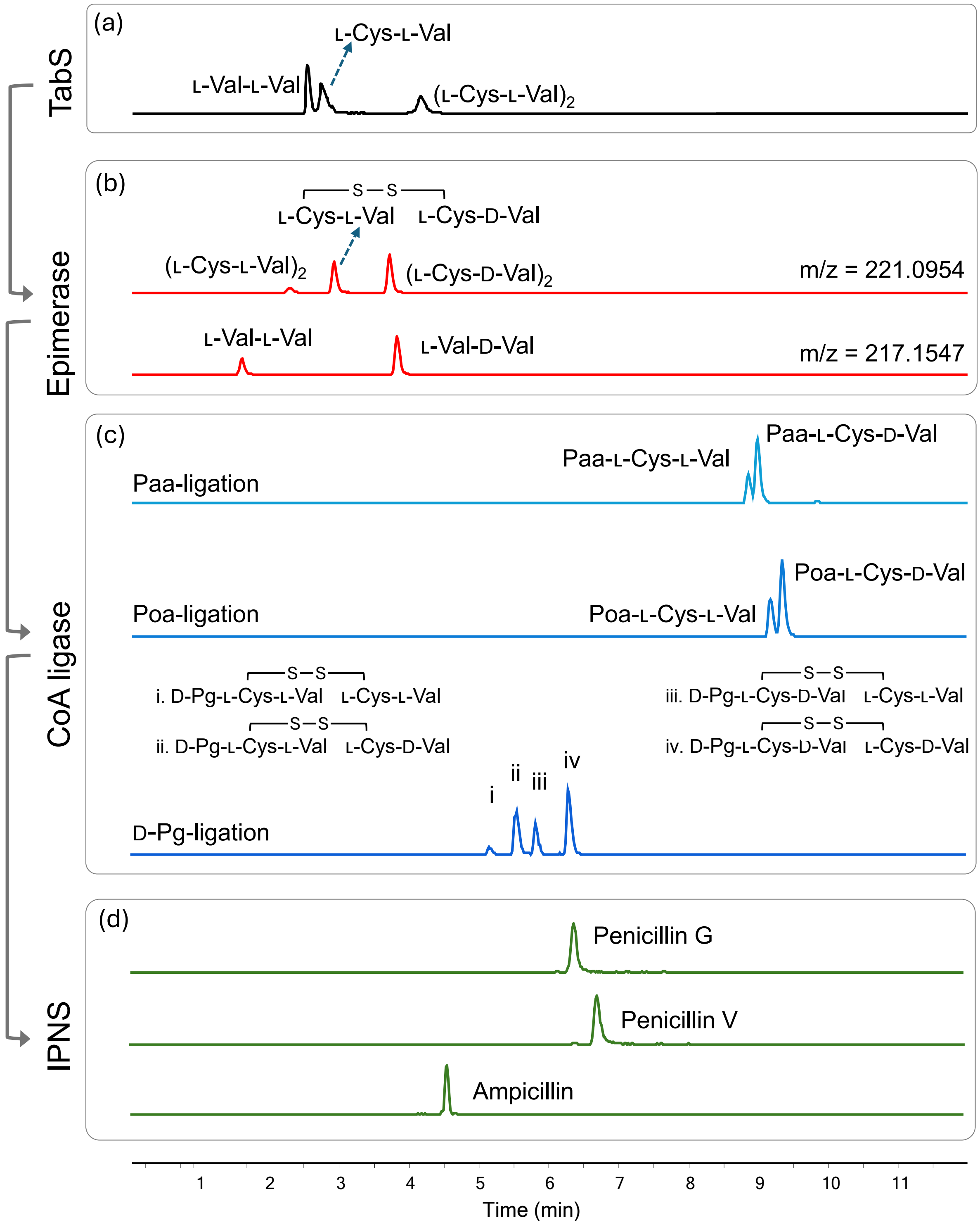

Supplementary Figure 14. EIC traces of the intermediates generated during the 4-enzyme cascade reaction starting from L-Cys and L-Val. Samples were taken after completion of each enzymatic reaction during the cascade reaction and analysed by LC-MS. (a) TabS reaction demonstrating the formation of the L-Cys-L-Val dipeptide and its dimeric disulphide form (L-Cys-L-Val)$_2$, together with the L-Val-L-Val side product. (b) Second reaction with epimerase enzyme showing the formation of enzymatic products L-Cys-D-Val and L-Val-D-Val, along with the unreacted substrate L-Cys-L-Val. (c) Third reaction using PhlA WT enzyme or A338G I266A mutant in the presence of Paa/Poa/D-Pg, demonstrating formation of N-acyl-L-Cys-L-Val and N-

acyl-L-Cys-D-Val tripeptides (this reaction also generates N-acyl-L-Cys as a by-product because of excess L-Cys required in the initial TabS reaction). (d) Final cyclisation reaction catalysed by caIPNS S185R R87L mutant enzyme resulting in the production of Penicillin G, Penicillin V or Ampicillin.

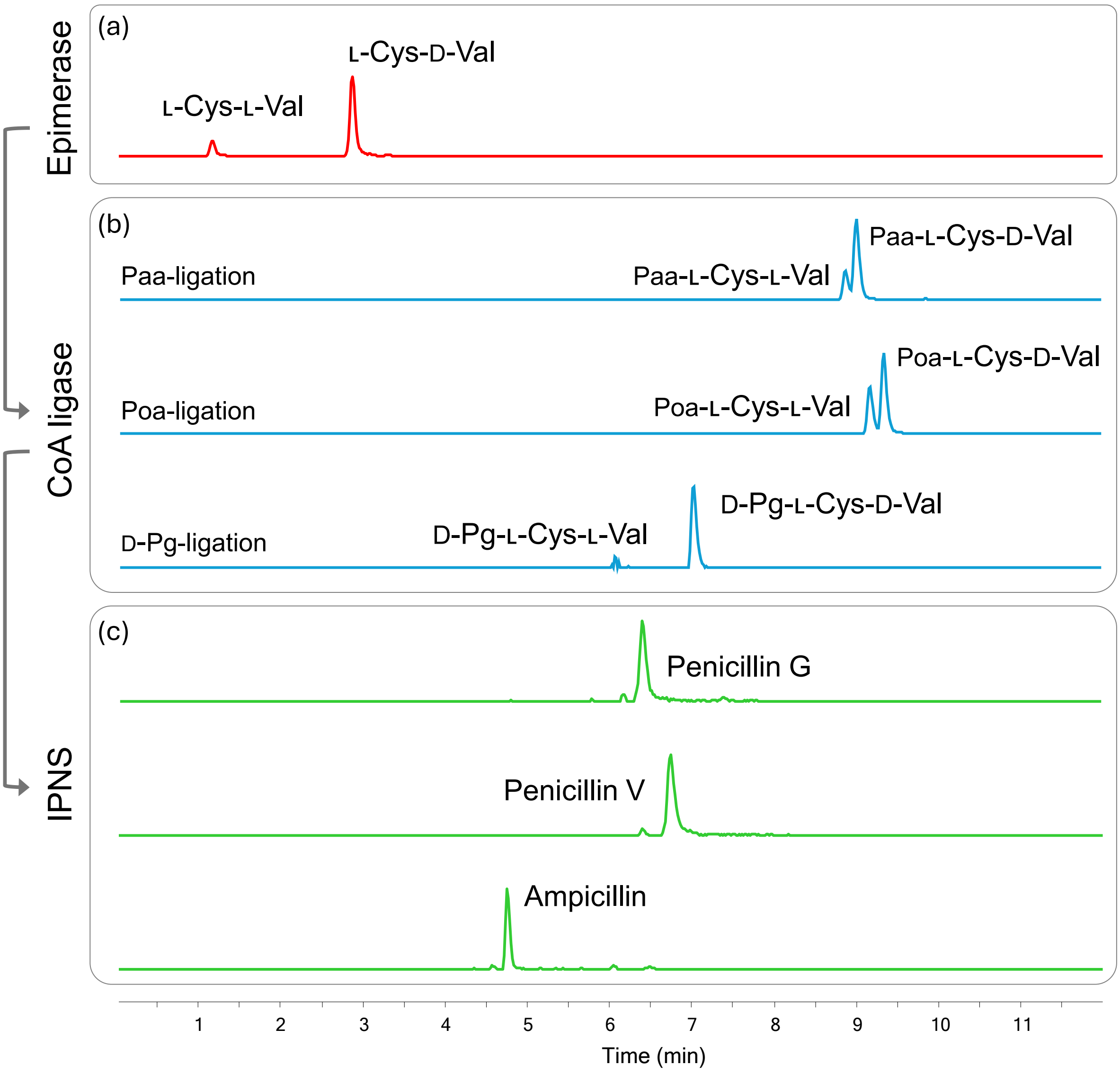

Supplementary Figure 15. EIC traces of the intermediates generated during the 3-enzyme cascade reaction starting from L-Cys-L-Val dipeptide. Samples were taken after completion of each enzymatic reaction during the cascade reaction and analysed by LC-MS. (a) Epimerase reaction demonstrating the production of enzymatic product L-Cys-D-Val along with the unreacted substrate L-Cys-L-Val. (b) The CoA ligase reaction using PhlA WT enzyme or A338G I266A mutant and Paa/Poa/D-Pg showing the formation of N-acyl-L-Cys-L-Val and N-acyl-L-Cys-D-Val peptides. (c) Final cyclisation reaction with caIPNS S185R R87L mutant enzyme resulting in the formation of Penicillin G, Penicillin V or Ampicillin.

NMR spectra of enzymatically synthesised compounds

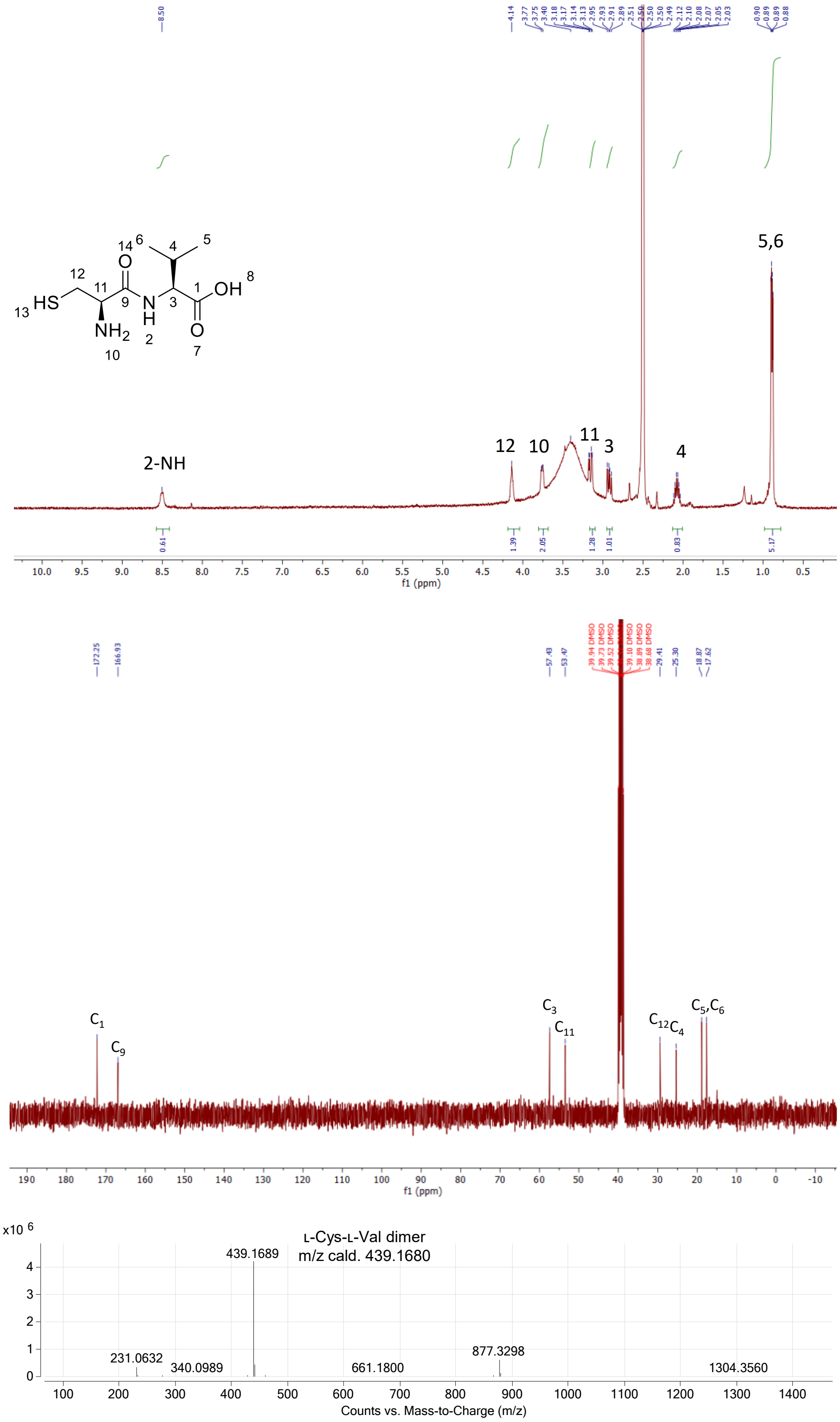

Supplementary Figure 16. ^{1}H and ^{13}C NMR and mass spectrum of L-Cys-L-Val dipeptide (m/z calcd. for $[M+H]^+$: 439.1680, observed 439.1689; Δppm = 2.04) derived from TabS assay.

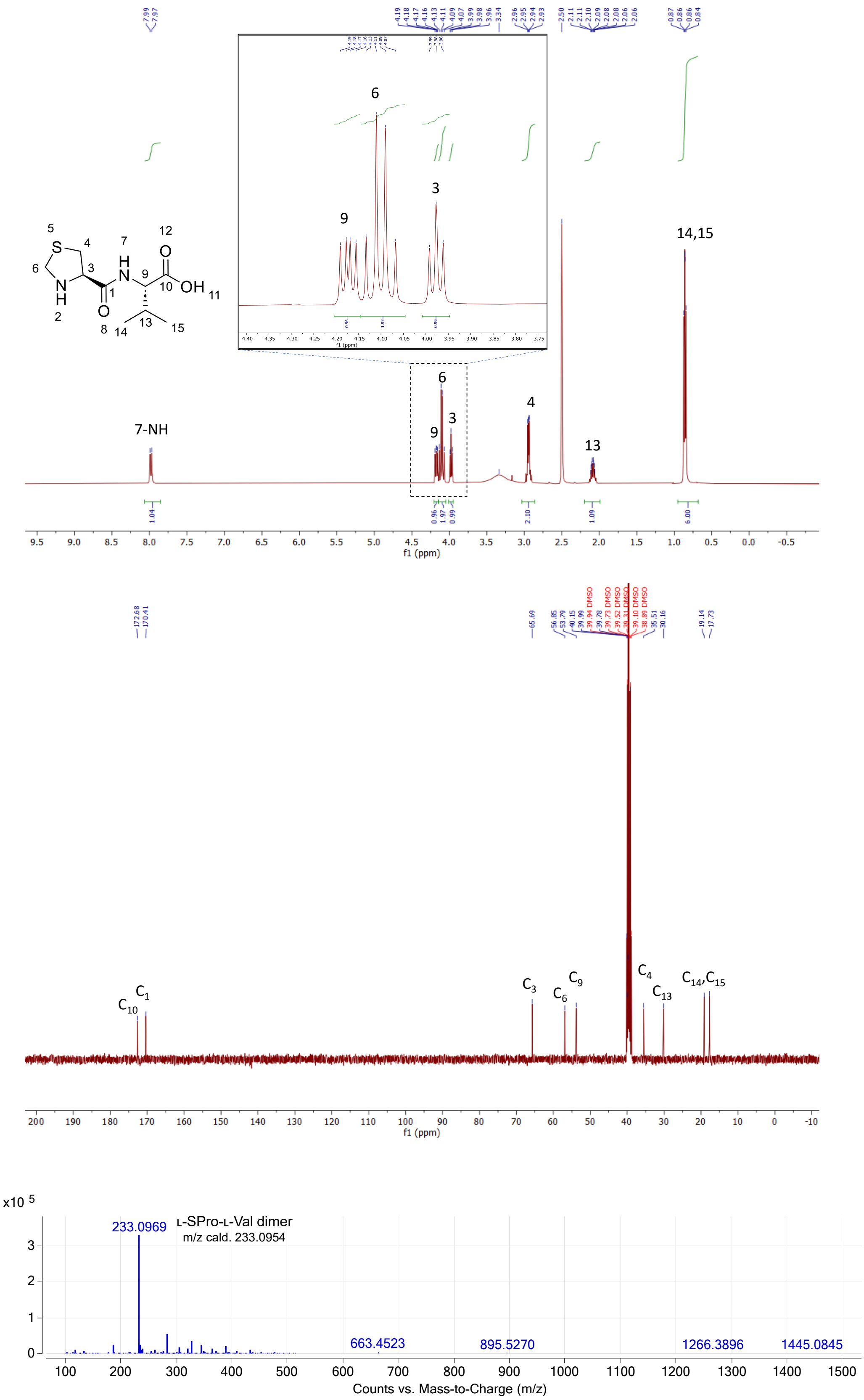

Supplementary Figure 17. ^{1}H and ^{13}C NMR and mass spectrum of L-SPro-L-Val dipeptide (m/z calcd. for $[M+H]^{+}$: 233.0954, observed 233.0969; Δppm = 6.4) derived from TabS assay.

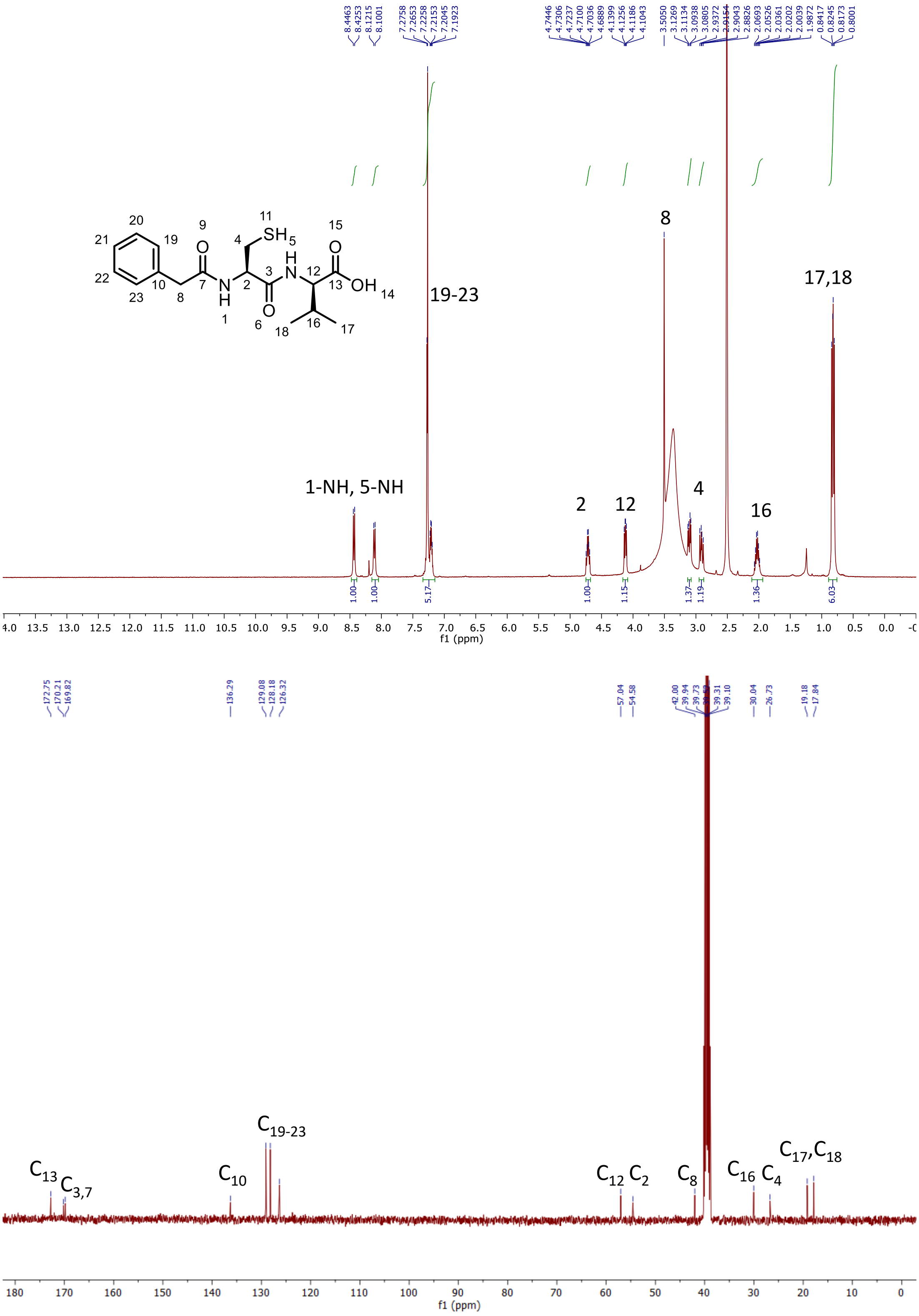

Supplementary Figure 18. 1H and 13C NMR of PaaCV derived from CoA ligase assay.

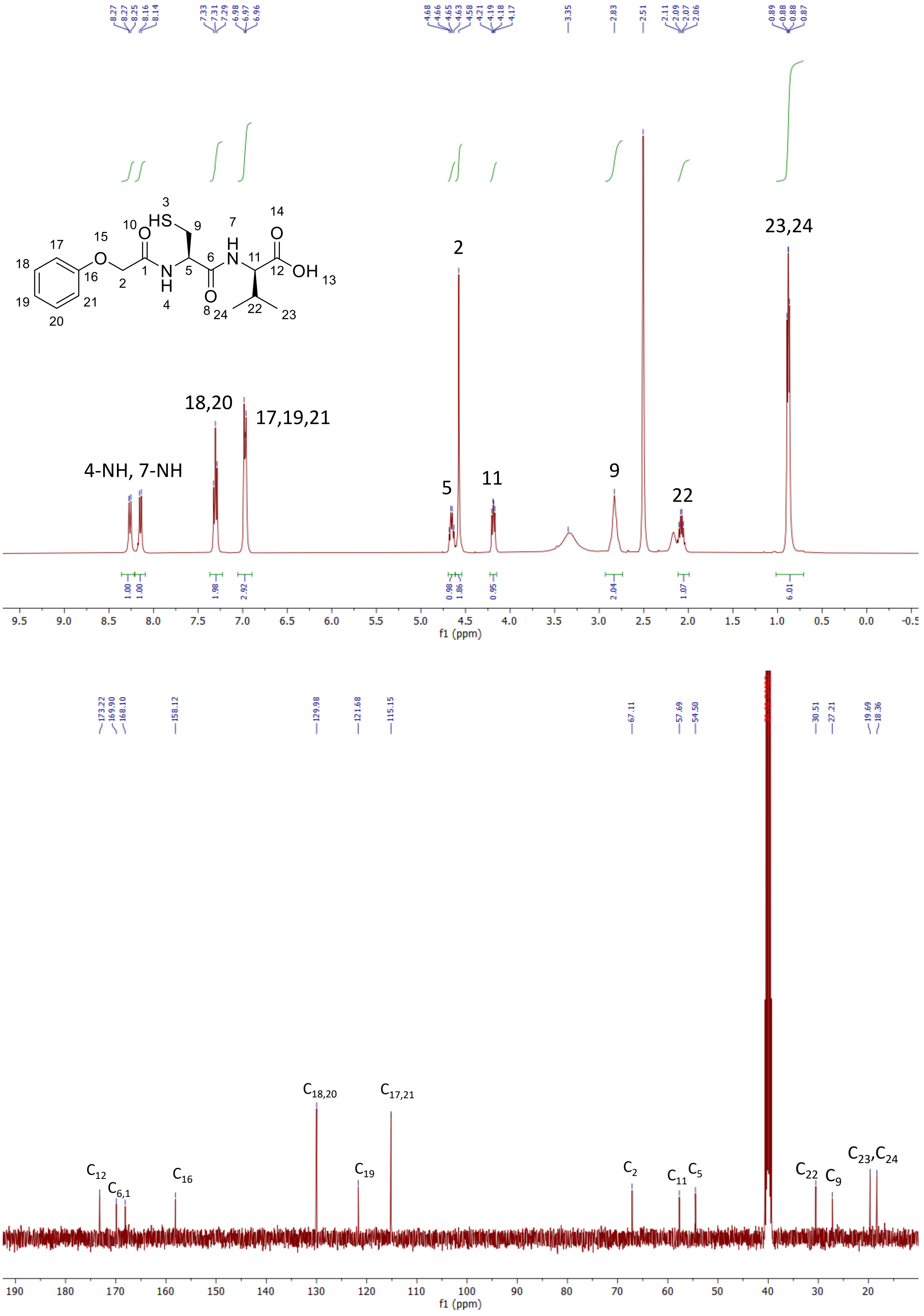

Supplementary Figure 19. ^{1}H and ^{13}C NMR of PoaCV isolated from CoA ligase assay.

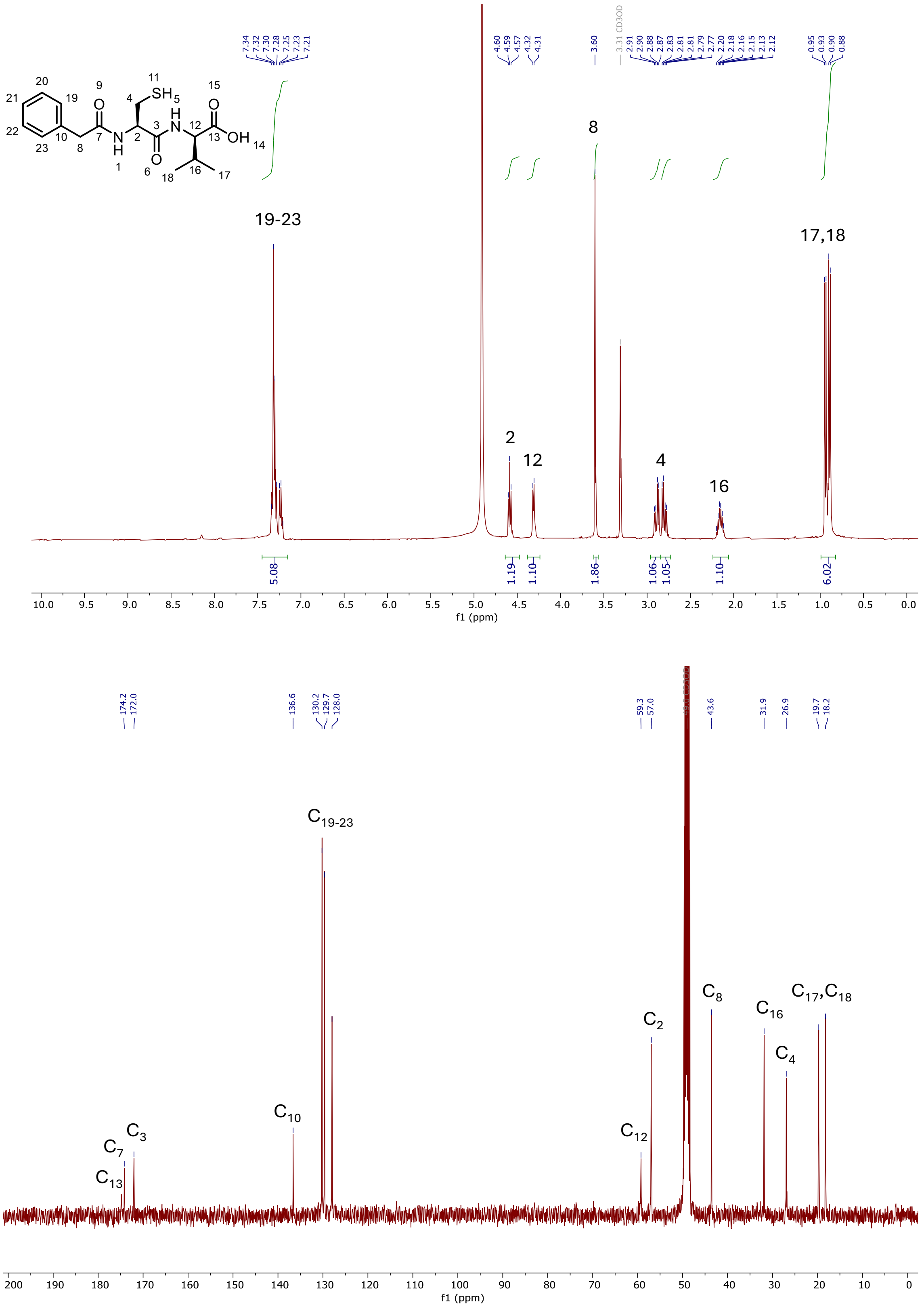

Supplementary Figure 20. ^{1}H and ^{13}C NMR spectra of PaaCV isolated from epimerase-PhlA cascade.

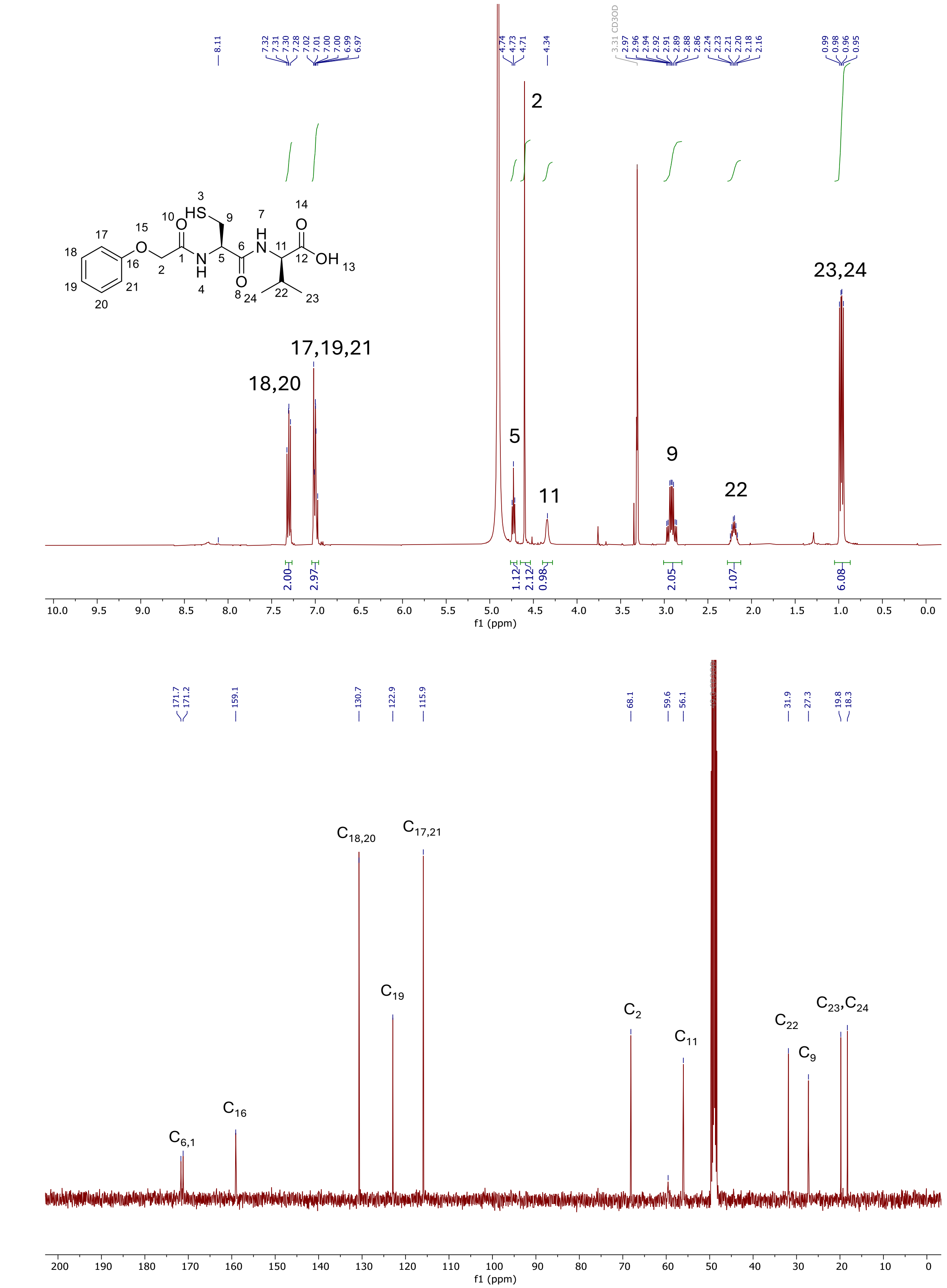

Supplementary Figure 21. ^{1}H and ^{13}C NMR spectra of PoaCV isolated from epimerase-PhlA cascade.

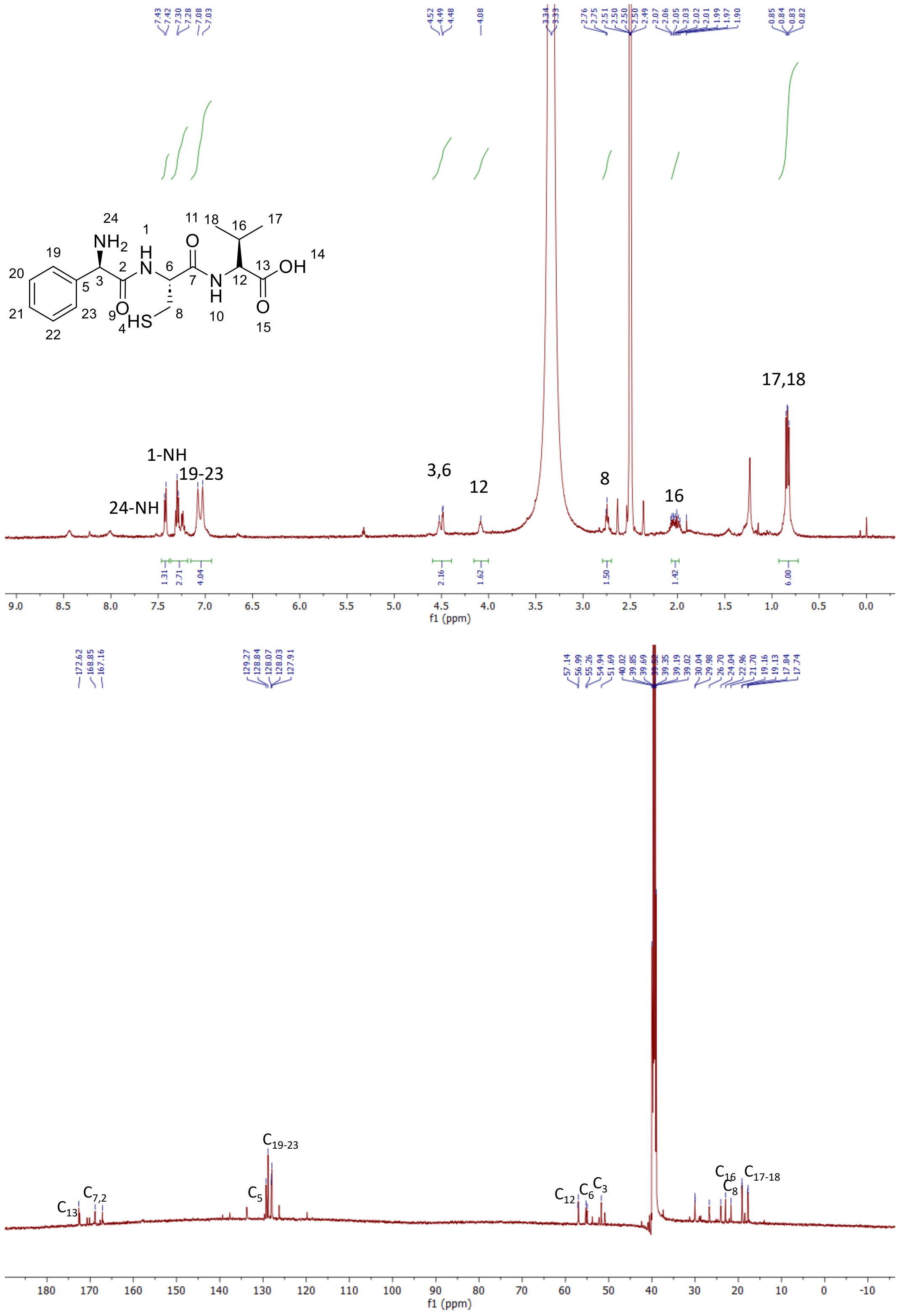

Supplementary Figure 22. ^{1}H and ^{13}C NMR of D-PgCV isolated from CoA ligase assay with PhlA A338G I266A.

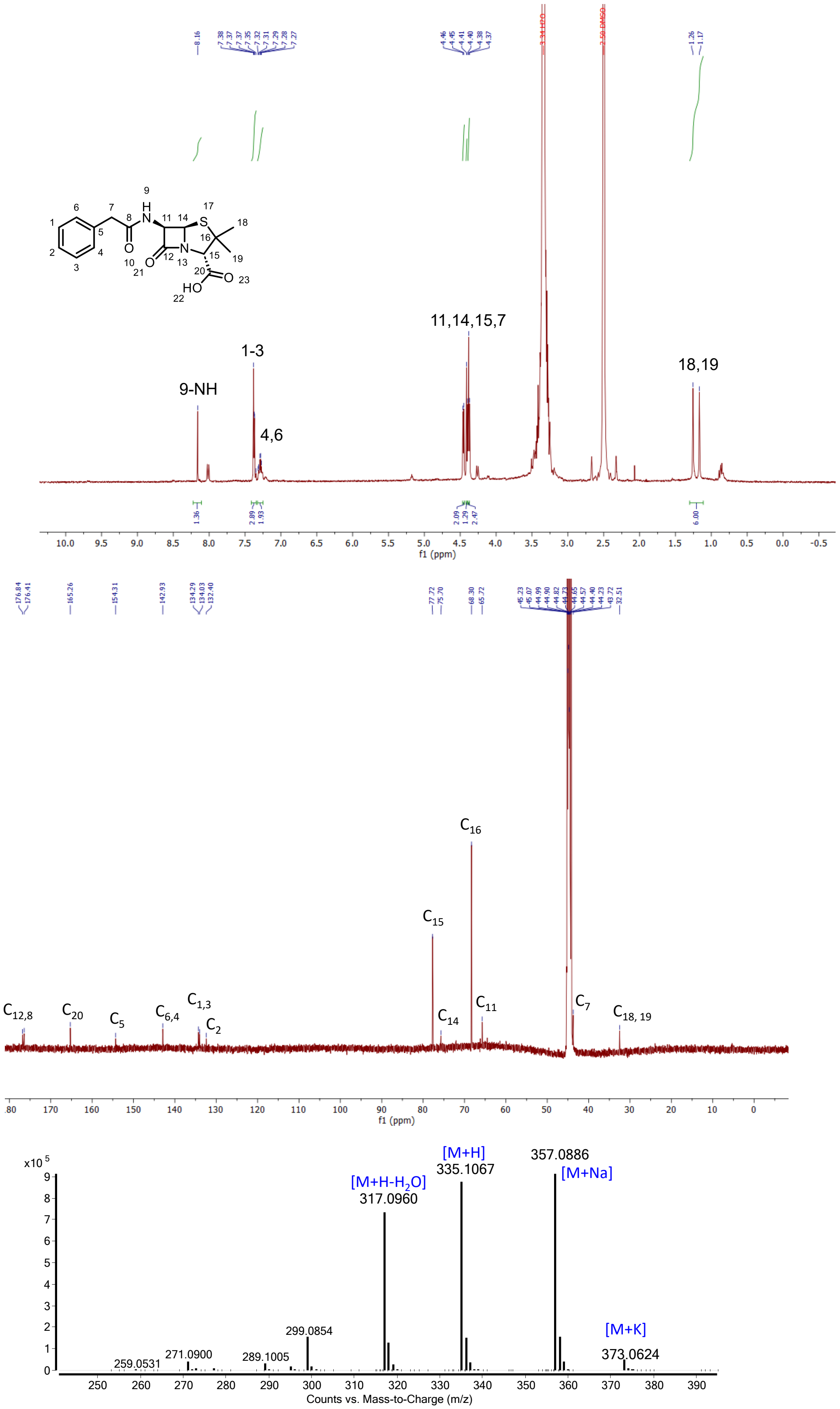

Supplementary Figure 23. ^{1}H and ^{13}C NMR and MS spectrum of Penicillin G isolated from IPNS assay with PaaCV (enzyme used: IPNS S185R R87L).

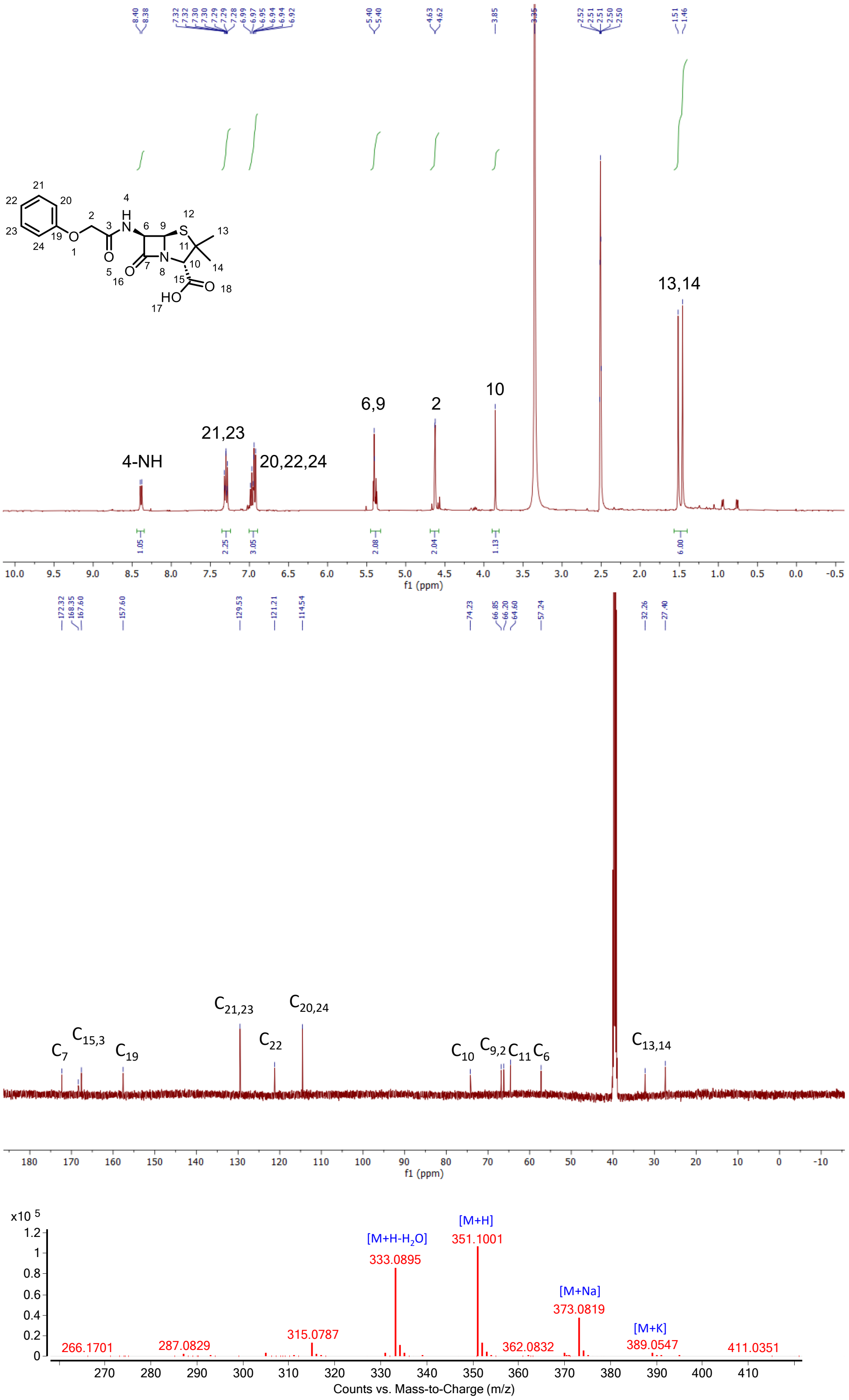

Supplementary Figure 24. ^{1}H and ^{13}C NMR and MS spectrum of Penicillin V isolated from IPNS assay with PoaCV (enzyme used: IPNS S185R R87L).

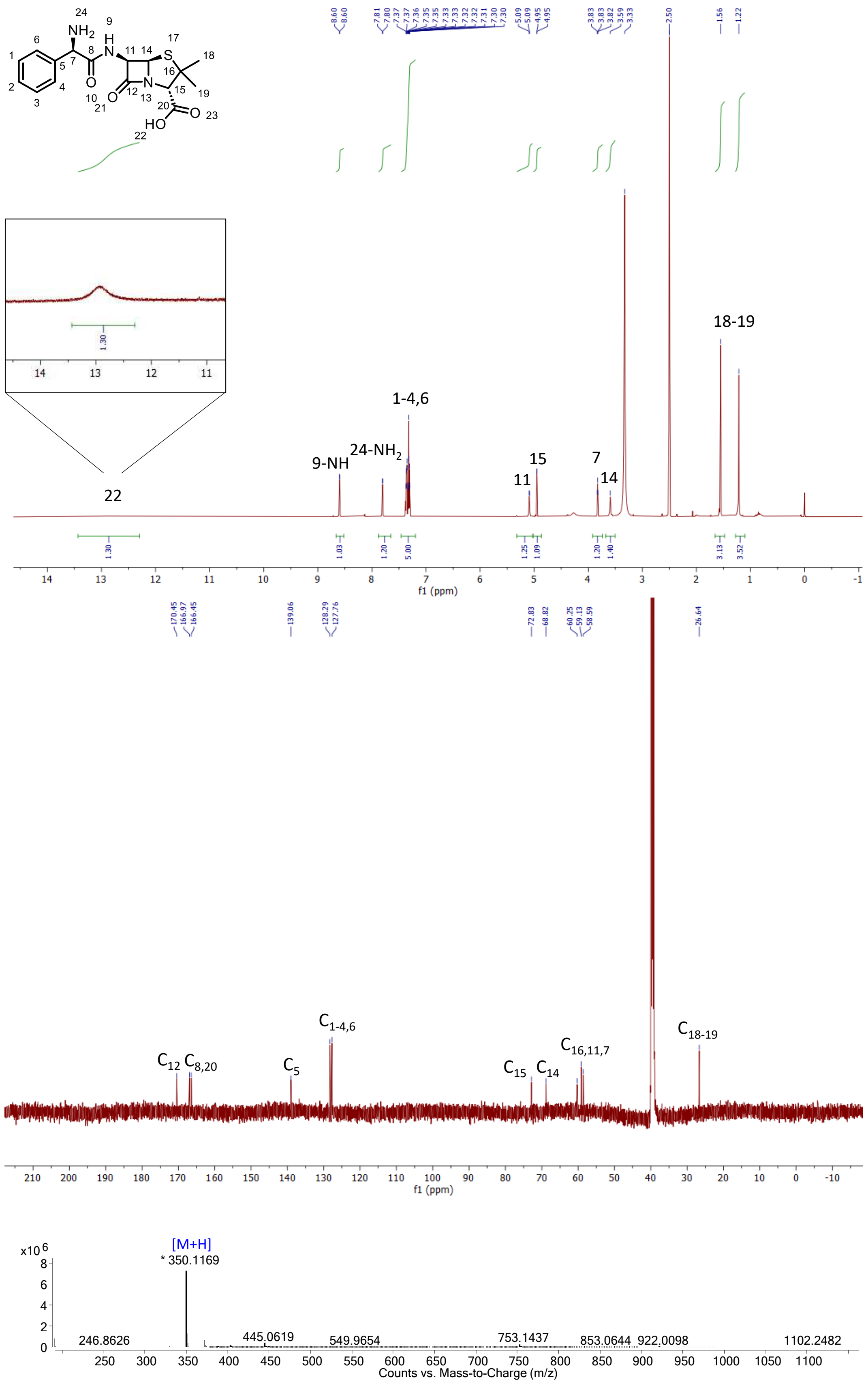

Supplementary Figure 25. ^{1}H and ^{13}C NMR and MS spectrum of Ampicillin isolated from IPNS assay with D-PgCV (enzyme used: IPNS S185R R87L).

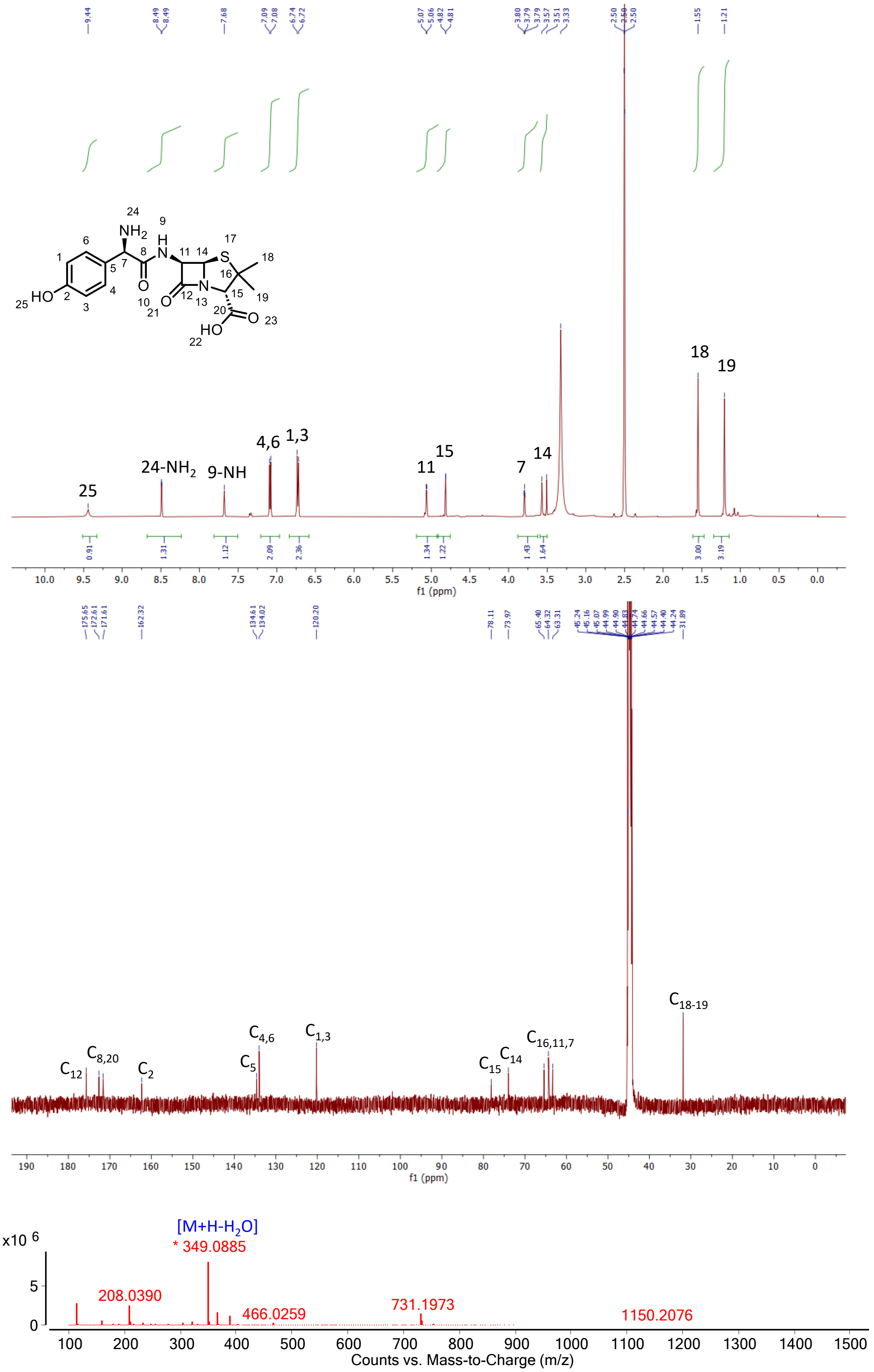

Supplementary Figure 26. ^{1}H and ^{13}C NMR and MS spectrum of Amoxicillin isolated from IPNS assay with D-HpgCV (enzyme used: IPNS S185R R87L).

NMR spectra of chemically synthesised compounds

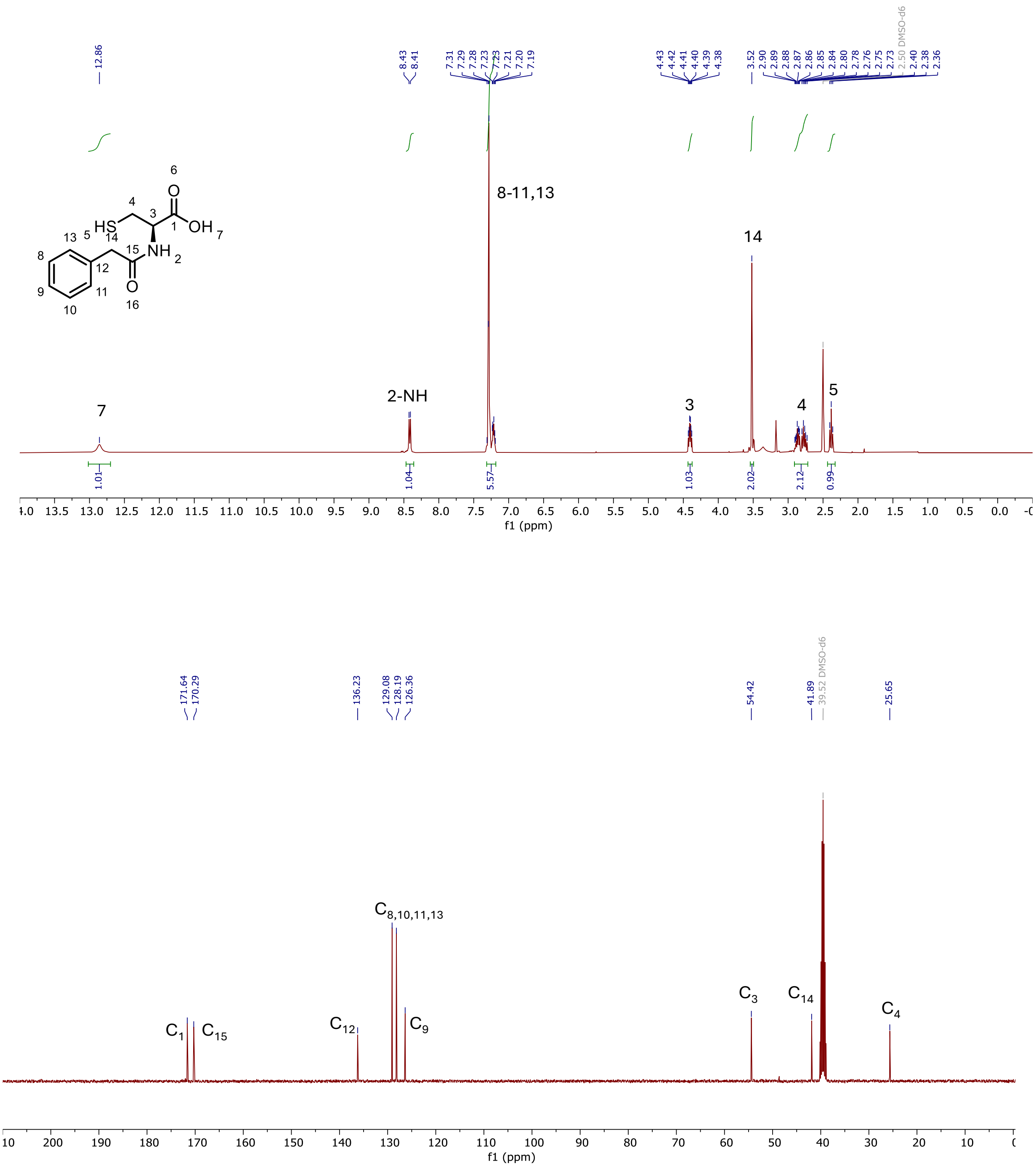

Supplementary Figure 27. ^{1}H and ^{13}C NMR of (2-Phenylacetyl)-L-cysteine.

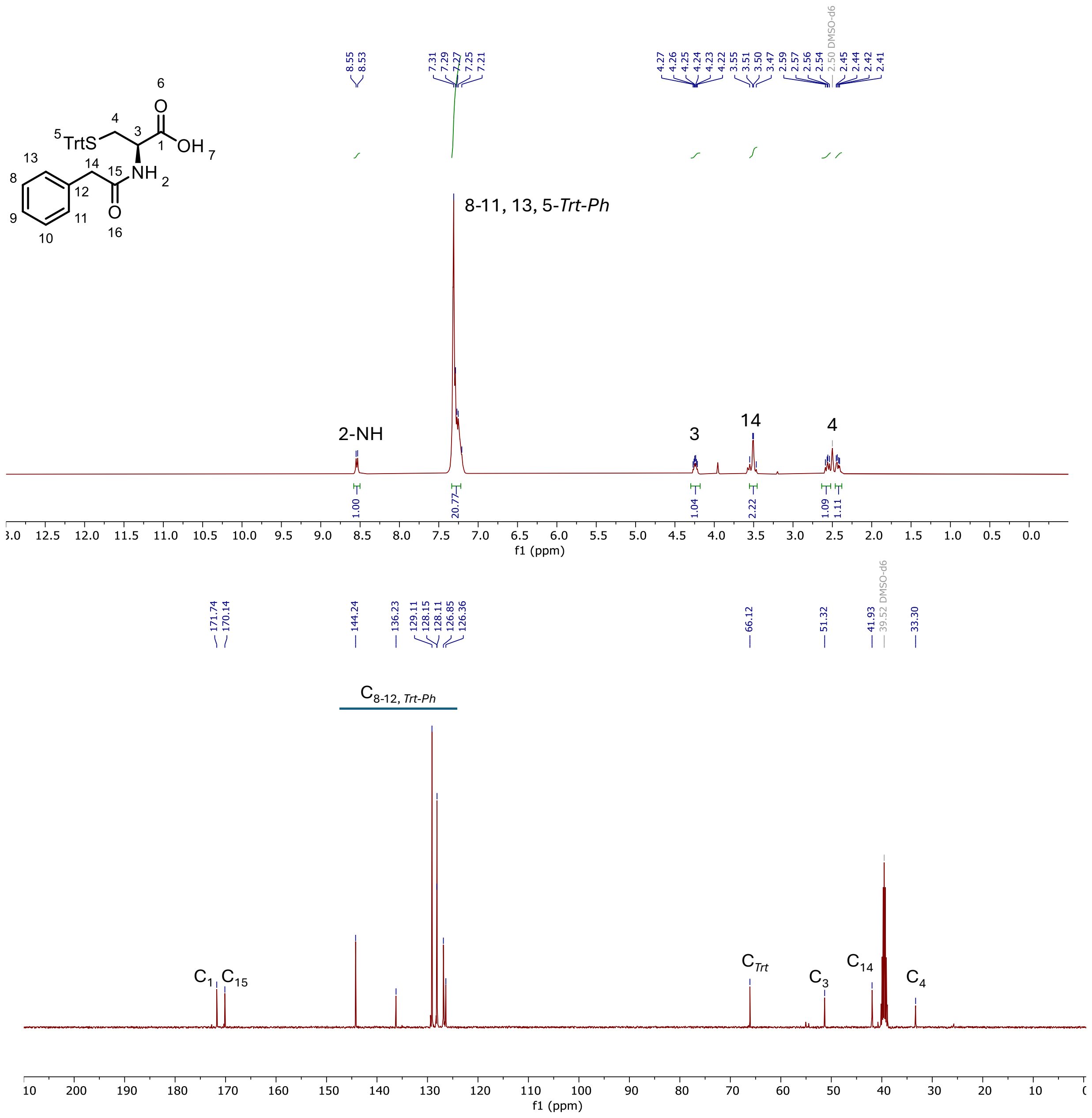

Supplementary Figure 28. ^{1}H and ^{13}C NMR of *N*-(2-phenylacetyl)-*S*-trityl-L-cysteine.

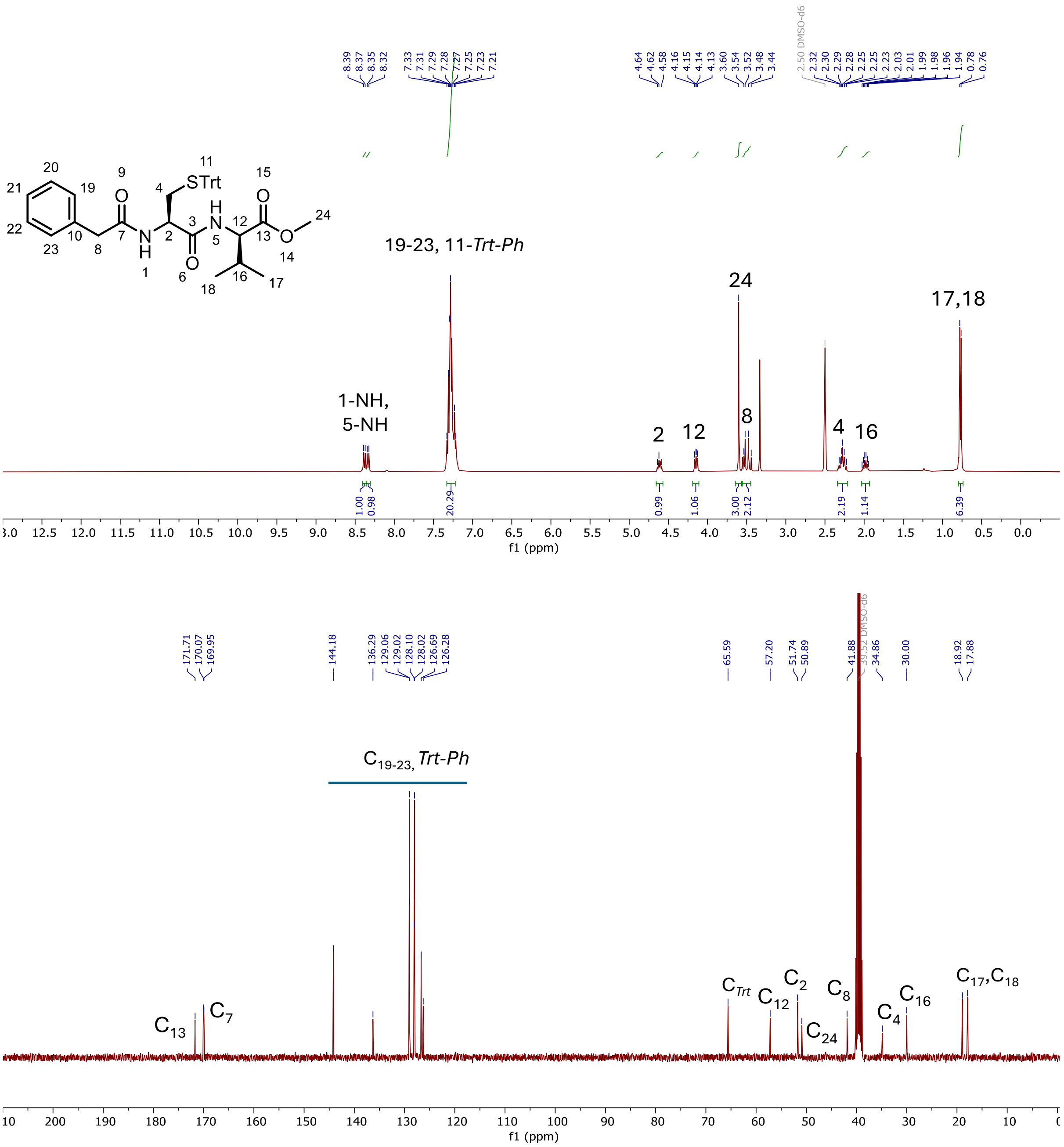

Supplementary Figure 29. ^{1}H and ^{13}C NMR of Methyl *N*-(2-phenylacetyl)-*S*-trityl-L-cysteinyl-D-valinate.

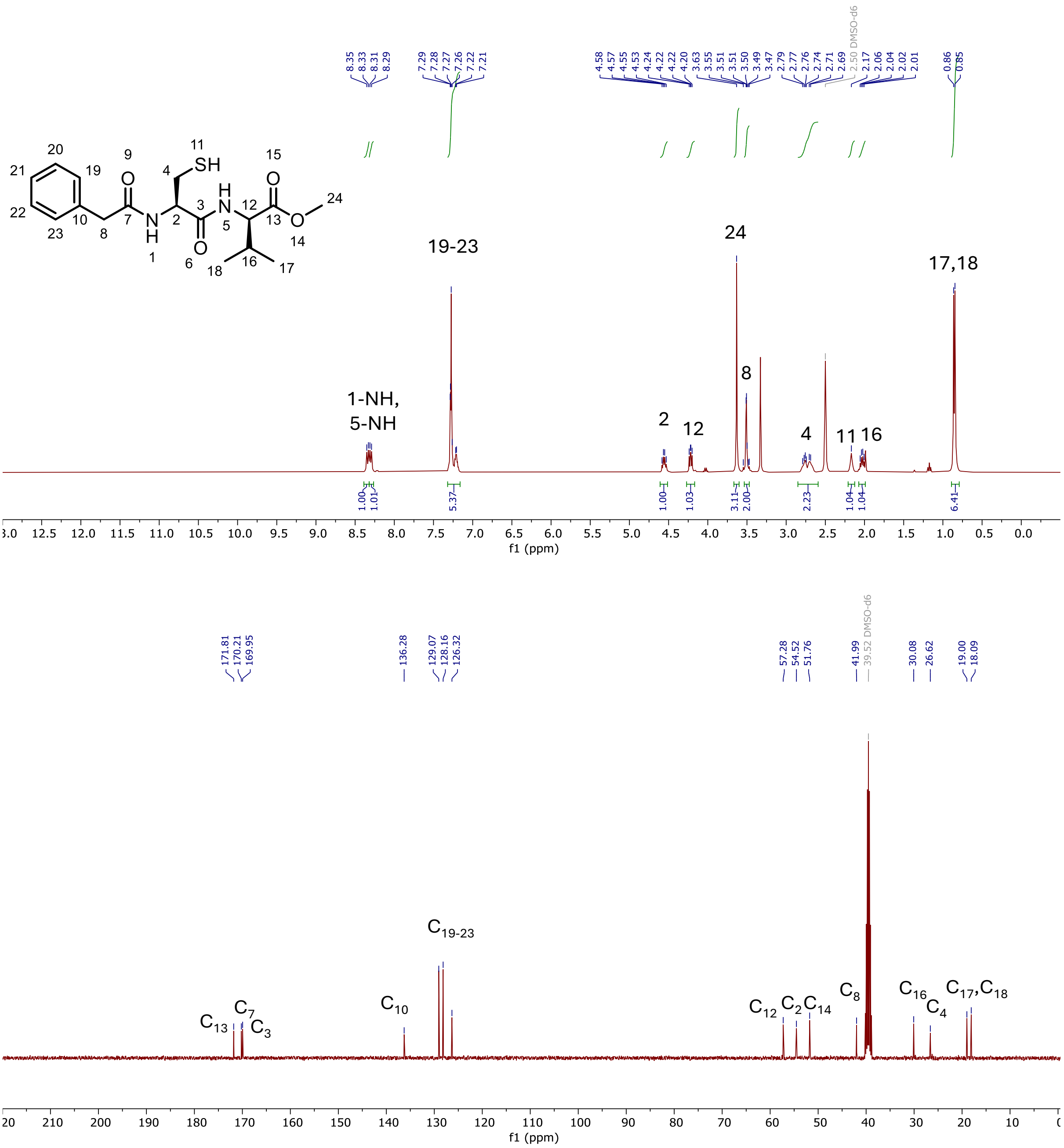

Supplementary Figure 30. ^{1}H and ^{13}C NMR of Methyl (2-phenylacetyl)-L-cysteinyl-D-valinate.

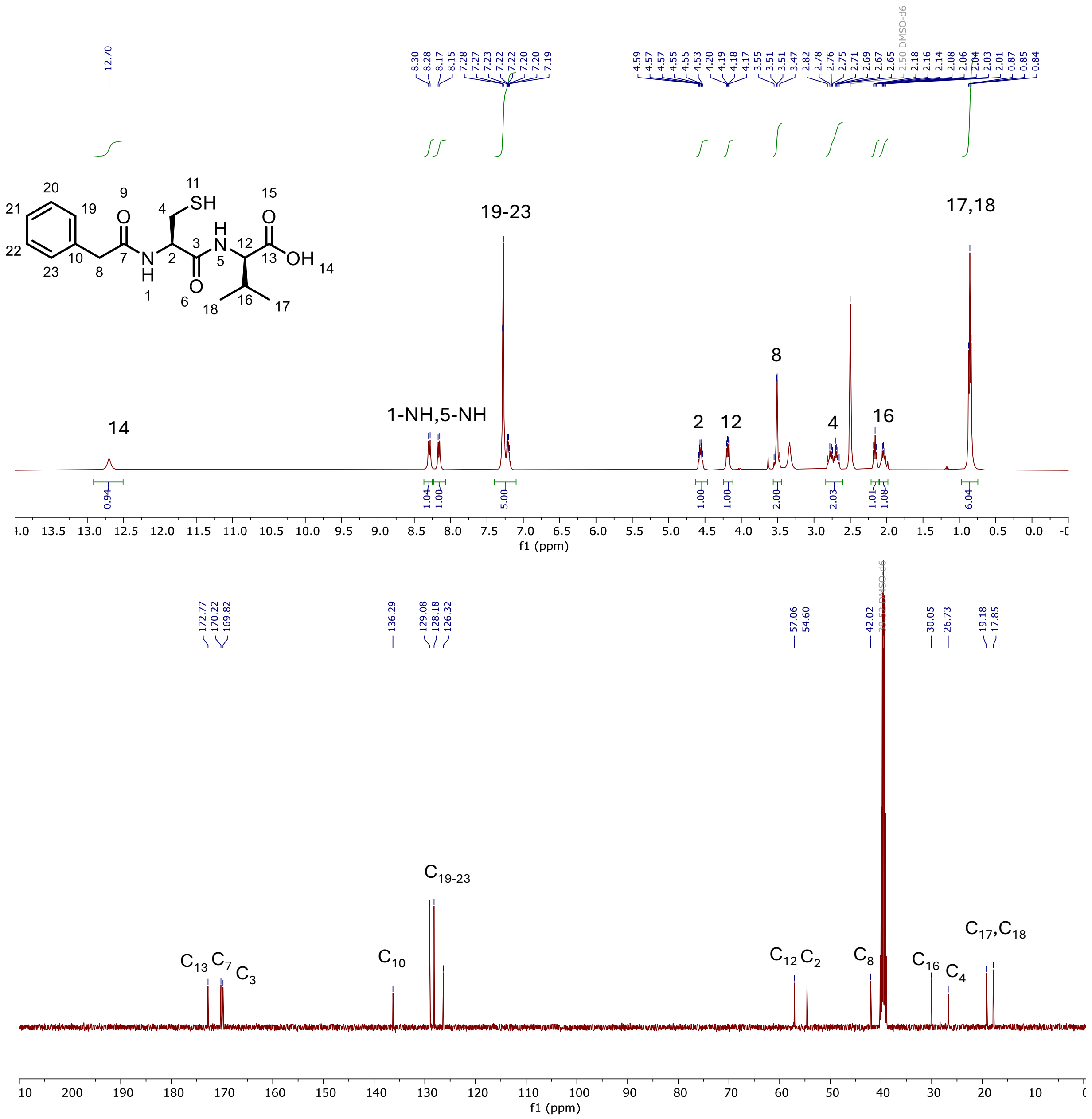

Supplementary Figure 31. ^{1}H and ^{13}C NMR of (2-Phenylacetyl)-L-cysteinyl-D-valine.

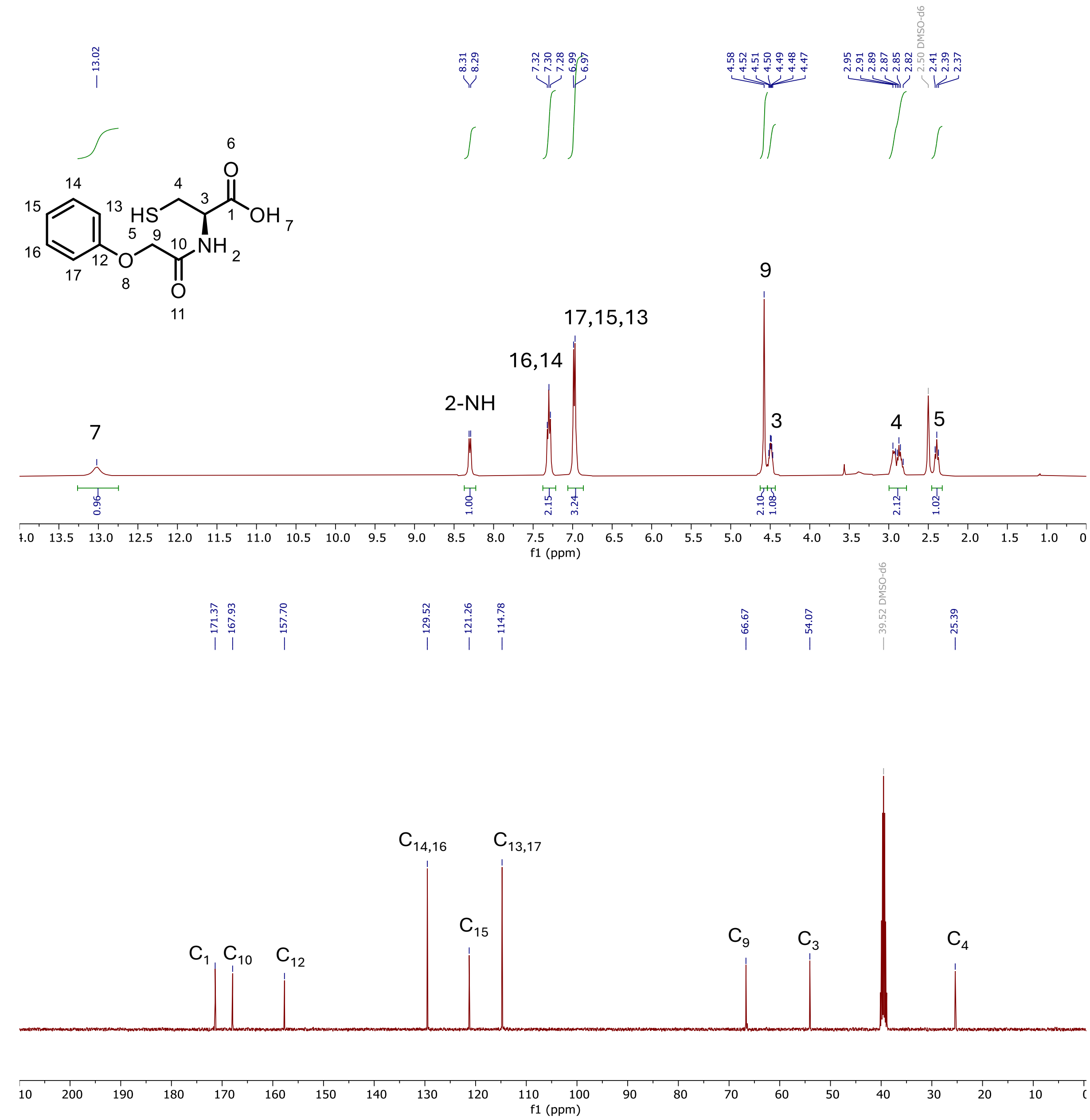

Supplementary Figure 32. ^1H and ^{13}C NMR of (2-phenoxyacetyl)-L-cysteine.

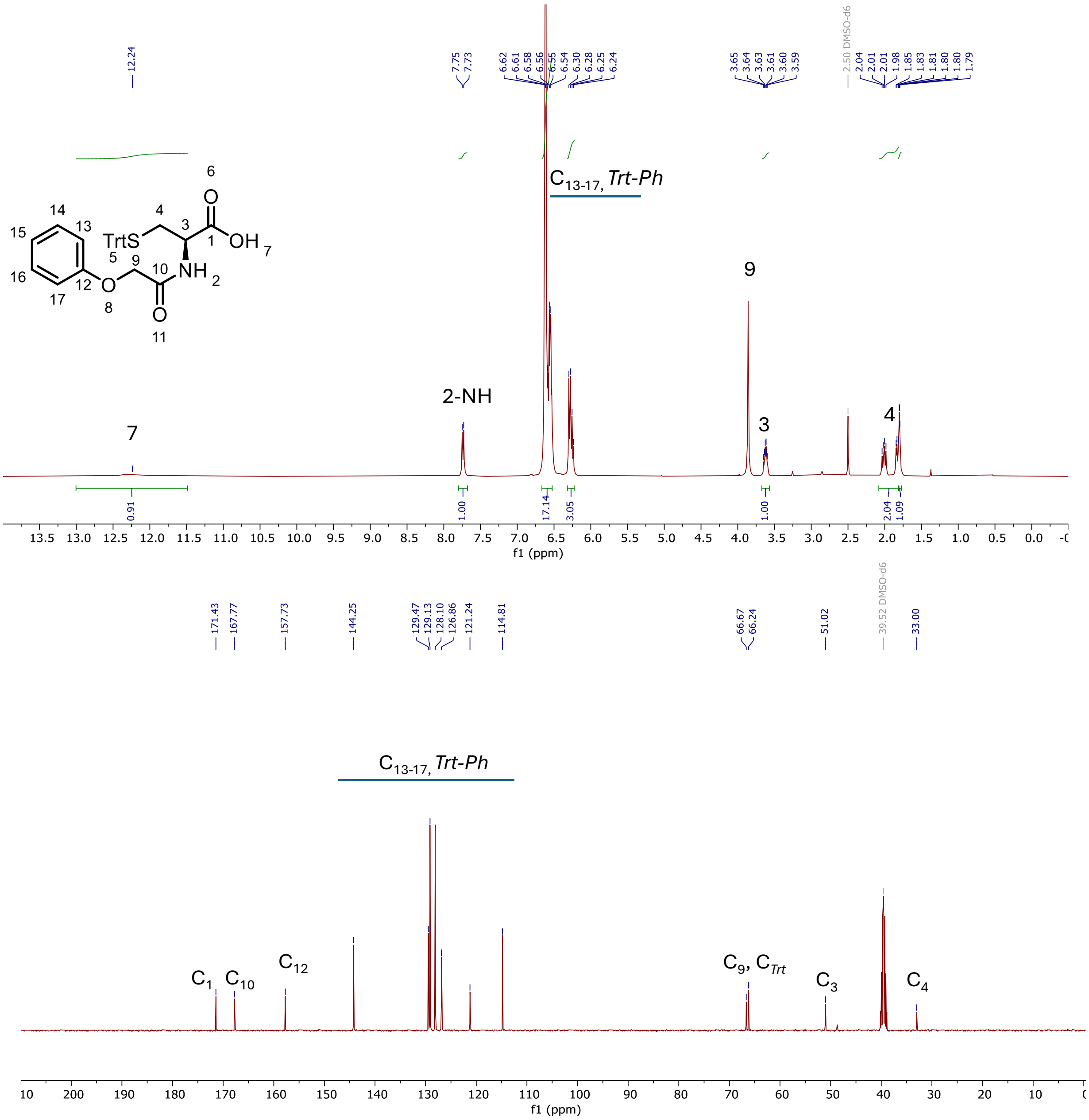

Supplementary Figure 33. ^{1}H and ^{13}C NMR of *N*-(2-phenoxyacetyl)-*S*-trityl-L-cysteine.

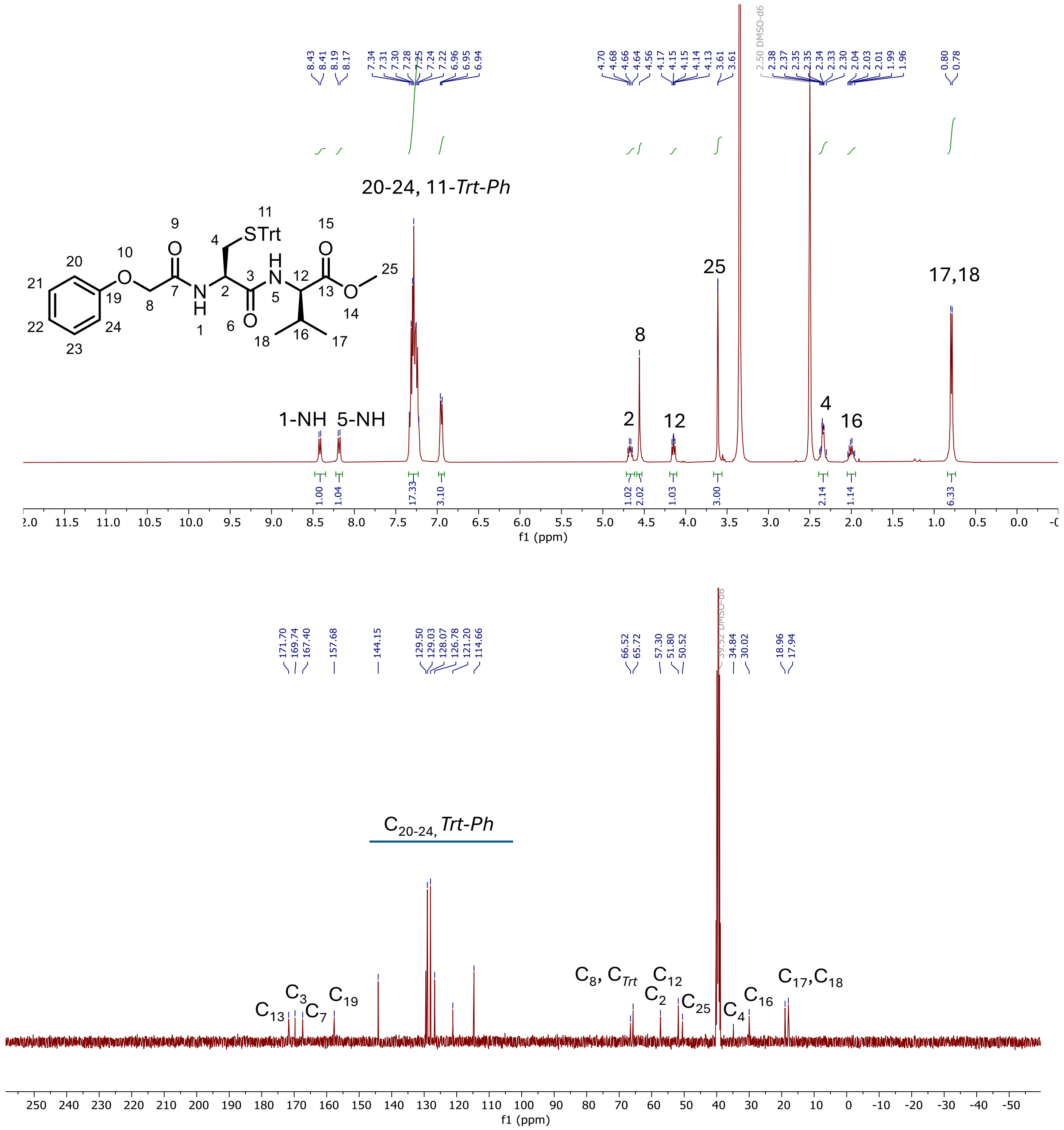

Supplementary Figure 34. ^{1}H and ^{13}C NMR of Methyl *N*-(2-phenoxyacetyl)-*S*-trityl-L-cysteinyl-D-valinate.

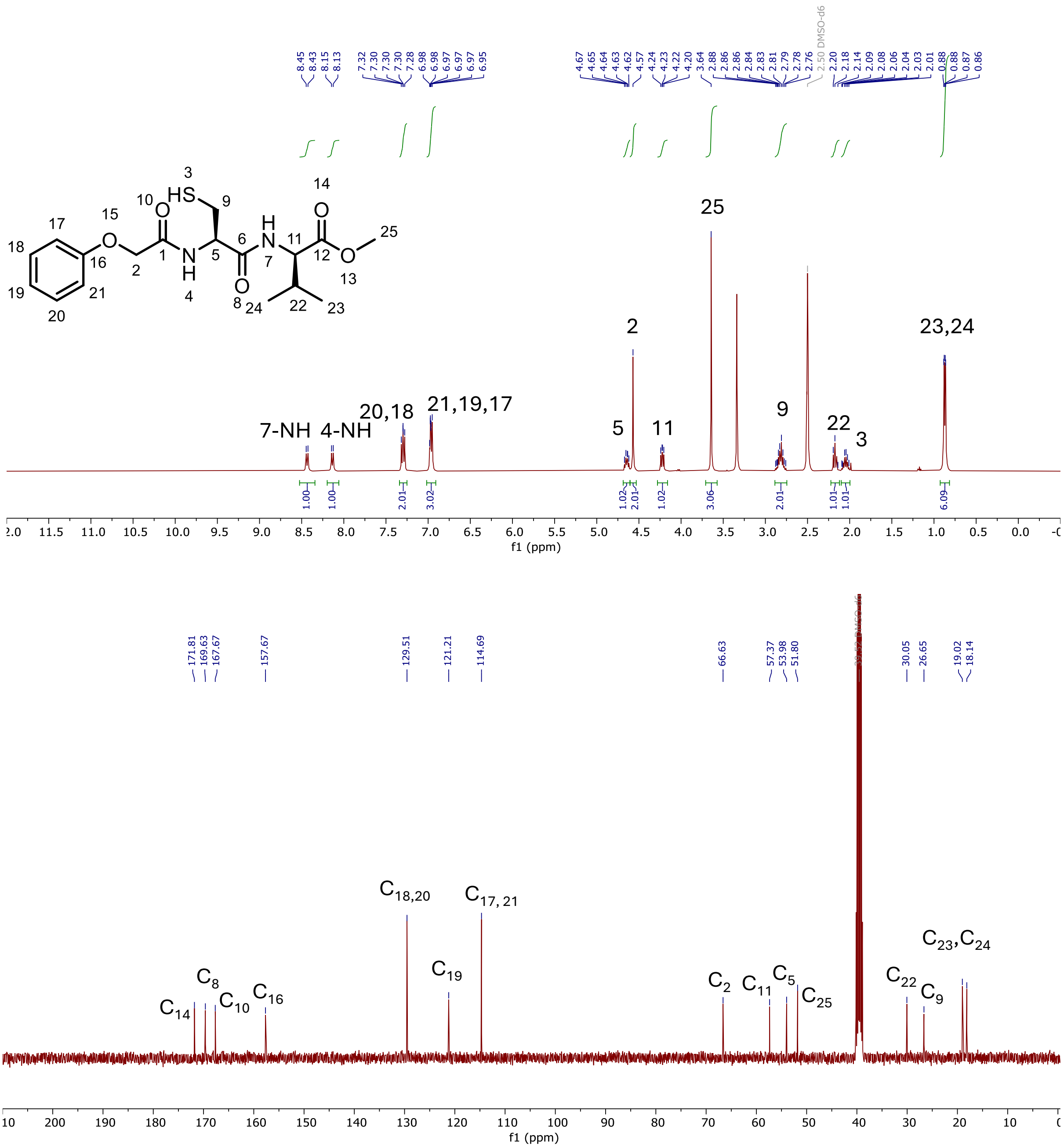

Supplementary Figure 35. ^{1}H and ^{13}C NMR of Methyl (2-phenoxyacetyl)-L-cysteinyl-D-valinate.

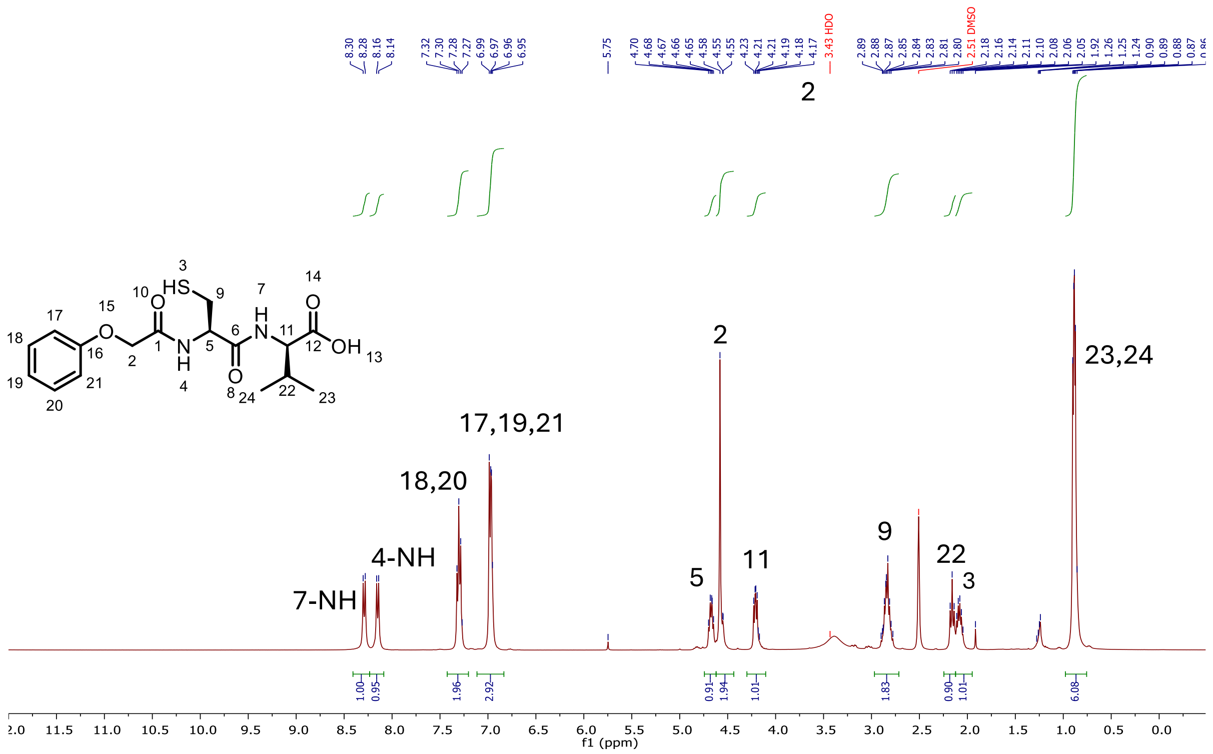

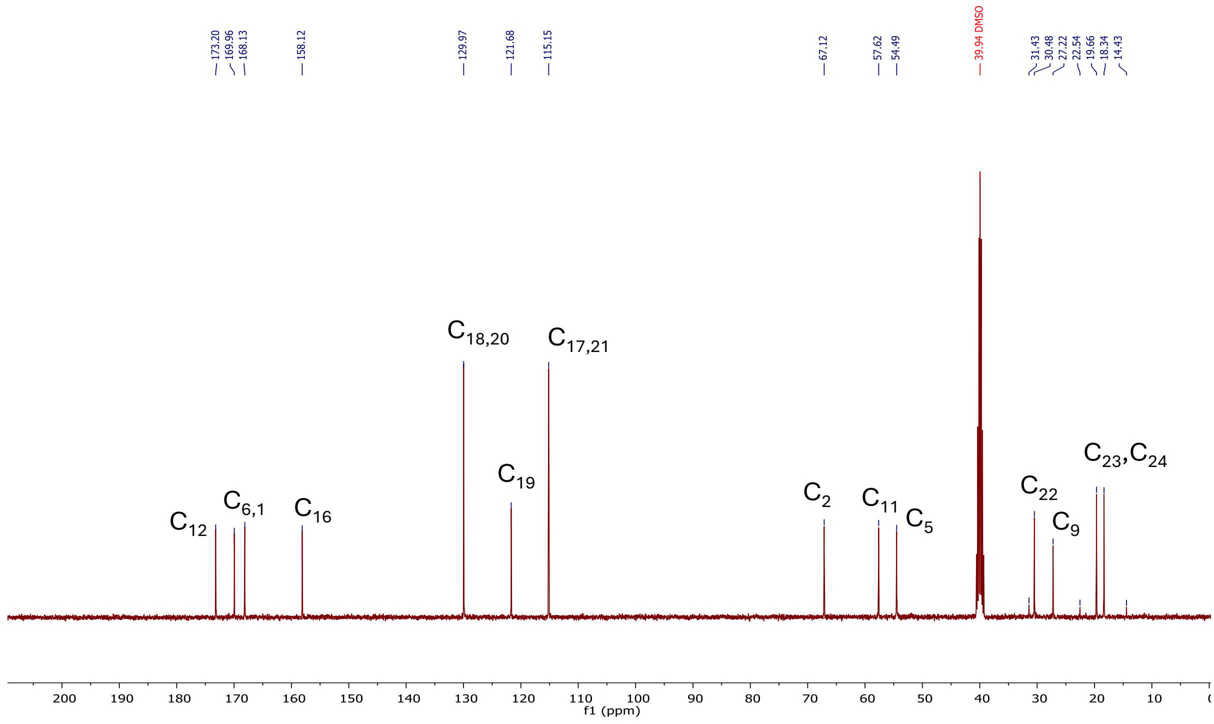

Supplementary Figure 36 ^{1}H and ^{13}C NMR of (2-Phenoxyacetyl)-L-cysteinyl-D-valine.

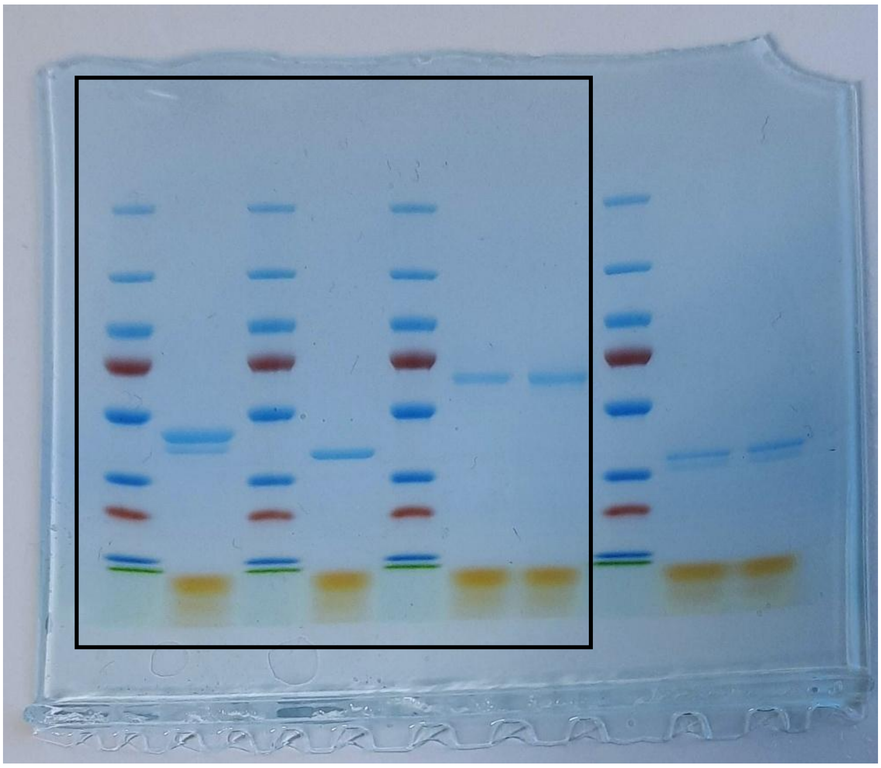

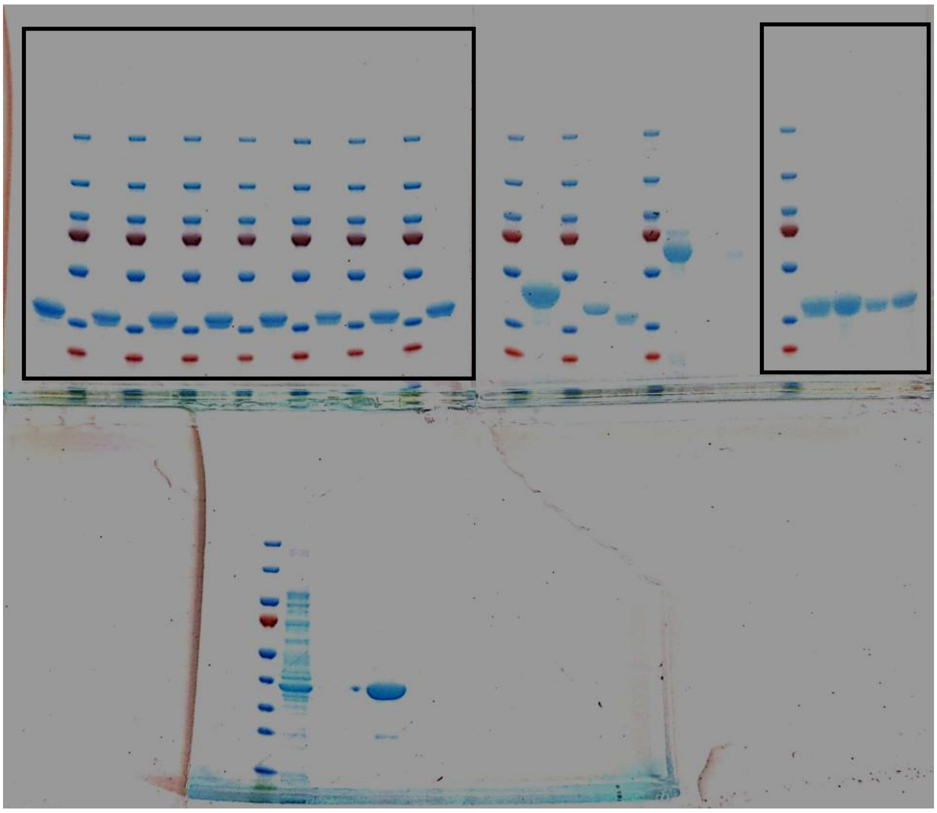

Supplementary Figure 37. The original scans of SDS-PAGE presented in Supplementary Figure 1.

Supplementary References

S1. Kalyanaraman, C. *et al.* Discovery of a dipeptide epimerase enzymatic function guided by homology modeling and virtual screening. *Structure* **16**, 1668–1677 (2008). https://doi.org/10.1128/AEM.01003-13

S2. Morris, G. M. *et al.* AutoDock4 and AutoDockTools4: Automated docking with selective receptor flexibility. *J. Comput. Chem.* **30**, 2785-2791 (2009). https://doi.org/10.1002/jcc.21256

S3. Li, Z., & Nair, S. K. Structural basis for specificity and flexibility in a plant 4-coumarate: CoA ligase. *Structure* **23**, 2032-2042 (2015). https://doi.org/10.1016/j.str.2015.08.012

S4. Oleg, T. & Arthur J, O. AutoDock Vina: Improving the speed and accuracy of docking with a new scoring function, efficient optimization, and multithreading. *J. Comput. Chem.* **31**, 455-461 (2010). https://doi.org/10.1002/jcc.21334

S5. Roach, P. L. *et al.* Structure of isopenicillin N synthase complexed with substrate and the mechanism of penicillin formation *Nature* **387**, 827–830 (1997). https://doi.org/10.1038/42990
