# Supplementary material for: Alternative enzymatic pathways to penicillin antibiotics
Source: Nat Commun. 2026 Apr 30;17:5891. doi: 10.1038/s41467-026-72466-w (PMC13338154; doi:10.1038/s41467-026-72466-w)
Supplement: Supplementary file 3 — Transparent Peer Review file [file 41467_2026_72466_MOESM3_ESM.pdf]

nature portfolio

Peer Review File

Alternative Enzymatic Pathways to Penicillin Antibiotics

Corresponding Author: Professor Jason Micklefield

This file contains all reviewer reports in order by version, followed by all author rebuttals in order by version.

Version 0:

Reviewer comments:

Reviewer #1

(Remarks to the Author)
In this work, Micklefield and coworkers demonstrate a novel biocatalytic route to access penicillin and derivatives. Instead of using the native NRPS biosynthetic machinery, they chose to utilize easier to evolve stand alone ligases and epimerases, in combination with native IPNS cyclases. They utilized TabS to generate the L-Val/L-Cys amide bond, taking advantages of its ability to utilize the unnatural L-SPro to avoid Val dimer formation and simplify purification. Epimerization of the L-Val to D-Val was accomplished using an epimerase from T. maritima, which was predicted to work based on docking studies with its crystal structure. N-acylation was cleverly install via utilization a Co-A ligase, which acylates the Cys, followed by an S- to N-acyl shift, similar to what occurs in NCL. While one of the ligases tested (PhlA) worked well for Paa and Poa (precursors to penicillin g and v) and even Phe to a small extent, it was unable to accommodate phenylglycine or hydroxyphenylglycine (precursors for ampicillin and amoxicillin). AlphaFold models combined with docking revealed several potential mutation sites, of which A338G and T369S enabled access to PgCV in moderated conversions. Unfortunately, no mutants had any activity towards hydroxyphenylglycine. After accessing the linear precursor, different IPNS enzymes were explored. It was found that an S185R mutant resulted in higher activities towards PaaCV and PoaCV. Ultimately, a mutant caIPNS was found to be the best for this conversion, in combination with a catalase buffer system to minimize ROS levels that are often observed with the Fe/ascorbate system. Further mutants of caIPNS S185R were explored based on AlphaFold3 models, with moderate improvements beings observed.

The manuscript is generally well written (a few grammar/typos) with a strong Supplementary Section supporting the main manuscript. The ability to biocatalytically produce penicillin and derivatives could be quite useful. However, it is unclear to me whether the described method is truly superior to the current semisynthetic method. As written, it appears that each intermediate is isolated before being subjected to the next enzyme in the cascade. It is also unclear whether this method is superior to simply chemically synthesizing the linear molecule followed by enzymatic cyclization by IPNS. If the authors could show a one-pot biocatalytic total synthesis using the enzymes that they discovered, this would greatly improve the paper and demonstrate improvements over the current methods. Additionally, the discussion about the ligases potentially being ancient biosynthetic pathways for the penicillins seems a bit far-fetched/not supported. While it is possible that the original penicillins were made by ligases, there is no evidence (e.g. ancient DNA with an IPNS present but no NRPS) that supports this. Overall, despite these issues, it is a strong paper that would be appropriate for publication in Nat. Commun. after major revisions. Please see below for other smaller revisions that are required in addition to those described earlier in this paragraph.

Major Revisions:
-ability to do a one-pot total biocatalytic synthesis (or at least the ability to feed unpurified products into the next enzyme) are needed to demonstrate the true utility of this approach over other approaches (e.g. semisynthesis or linear synthesis followed by IPNS)
-The discussion of the evolution seems far-fetched

Minor Revisions:
- In figure 1a, “ACV” the molecule and “IPNS” the enzyme are in the same font/bolded – some distinction should be made
- Figure 1a, cephalosporin spelled wrong – spelled “cephalosprin C”
- Line 60, after “cyclisation by IPNS would generate ampicillin and amoxicillin” they should add “, respectively”
- Line 66, they say “ACVS” for the first time but do not define its definition – previously “ACV synthetase”
- Line 65, change “group, combined with the necessity” to “group; this, combined with the necessity”
- Line 88, “cyclised by IPNS variant” to “cyclised by an IPNS variant”

- Line 97, remove either “involving” or “using”
- Val dimerization is observed. Is cysteine dimerization every seen with the TabS? Just curious
- In general, some of the grammar needs correcting.
- Line 118, clain that L-Val dimer is 4%. That said, Figure 2C, it looks like there is more than 4% of the dimer. Is the graph correct?
- Line 191 – Any thoughts on why this did not work w D-Hpg? More explanation is needed
- The mutagenesis studies should be summarized in a table rather than shown in the bar graph. That would make it easier to determine the differences
- Figure 2, there is a box around TabS that should be removed
- Line 122 – need to define methoxyamine hydrochloride acronym for figure 2a
- Need to be clearer/more consistent with labeling of compounds/acronyms
- Figure 3e – duplicate only? Why not triplicate; also, if only duplication, I do not think it is correct to show error bars
- Figure 3f – a “no enzyme” control should be shown
- Figure 4c – needs to be better quality/a little bigger (or shown as a table rather than a bar graph)
- In general, the organization of the SI could be improved. It would be better to have all of the supplemental methods together then the SI Figures then the SI tables.
- SI Cloning and Mutagenesis section, what antibiotic and what concentration was used for protein expressions?
- Need to give sequences of primers used
- SI fig 9 – table form would be better or a good addition
- SI fig 10 – need to a), b) , c) to figure legend. Also, how many replications for SI figure 10 panel C?
- Add delta ppms for all mass specs
- SI Figure 17, C13 for the PaaCV dimer does not have good enough S/N
- SI Figure 21, no 13C? Also the annotation for this 1H NMR is different from others (do not outline which proton goes with what)

Reviewer #2

(Remarks to the Author)
I co-reviewed this manuscript with one of the reviewers who provided the listed reports. This is part of the Nature Communications initiative to facilitate training in peer review and to provide appropriate recognition for Early Career Researchers who co-review manuscripts.

Reviewer #3

(Remarks to the Author)

I like the concept underlying this paper, i.e. to 'replace; natural occurring non ribosomal peptide synthetases with engineered small peptide synthetases to make precursor peptides for cyclising to medicines, in this exemplar case penicillins.

The sequence is promoted as being a potentially useful alternative to the established semi-synthesis route to penicillins. At the concept stage commercial viability is not a prime concern, however, The cost of producing even one, let alone multiple recombinant proteins. is surely going to be greater than that of a fermented penicillin. The current procedure is in fact highly efficient and penicillin availability is principally limited by regulations narrowing its production to specialized facilities.

Nonetheless, to repeat I really do like the concept, but there are several key points that need clarifying. Firstly as I understand from the manuscript, all the individual enzyme reactions were optimized to produce products that could be used by the next enzyme in the sequence. It was not clear to me however, whether the products that were produced in one enzyme catalysed step were passed on to the next enzyme or whether synthetic materials were used. What is the overall efficiency of the entire enzyme catalyzed procedure?
Further can the entire enzyme catalysed procedure be used in the same pot? If not what is the most efficient procedure? At least some attempts to optimise the overall procedure should be presented. The authors did manage to combine the the epimerase and PhlA enzymes productively, so must have tried other combinations?

Ideally of course the entire process would be engineered into cells - was this considered or is it the subject of ongoing work. It would seem likely this is the only way that the new procedure could possibly compete with the existing highly efficient procedure.

There is also very little information on the kinetics of the individual enzyme catalyzed reactions. Information on what scale the entire enzyme catalysed procedure can be carried out on would be useful.

Our final point is whether or not the work really relates to evolution - it's attractive to propose that small molecule synthetases evolved before the more complex non ribosomal peptide synthetases, but I'm not sure there's any actual evidence for this, at least for those studied (glutathione is not in all bacteria?). My own feeling is the work is better presented as a novel

sequence of reactions leading to the precursor tripeptide for penicillin formation, rather than as an ancestral / evolution type story.

Overall I'm keen on the work but the issues above need to be addressed in particular with respect to whether or not the entire sequence can be catalyzed in an enzyme catalyzed way and whether some or all the individual reactions can be combined.

Comments on the main text

Please think about the title - is the use of ancestral really justified? What is the evidence? Maybe" "A novel enzymatic pathway to penicillins" or some variation on this. The abstract is fine - it does not overclaim.

Introduction

L47 IPNS is not a synthetase as far as I know - ATP not involved?

L50 delete " laborious" - the route is super-efficient - penicillins are cheap.

Results

please comment the efficiency of on the ligase, epimerase, cylation reactions- at least in terms of specific activities and in selected cases by kcat / Km values.

Line 141 "therefore proceeded to the next step with the ʟ-Cys-D-Val dipeptide"

Cyclisation of peptides into β-lactam antibiotics.
Line 196 IPNS is an oxidase not an oxygenase as no O2 derived O atoms in its product? Please correct.

Please comment on the catalytic efficiency of the mutants - at least in terms of specific activities and in selected cases by kcat / Km values.

Discussion
Perhaps a bit of clarification useful: These antibiotics likely arose through
convergent evolution with respect to the b-lactam ring forming reactions. ??
Please check are all beta-lactam formed from peptides - what about clavams?

Figure 2. "the ʟ-Val-ʟ-Val side product."

(d) Assay showing the epimerisation

Figure 3e/4c

D-phenylglycine/D-hydroxyphenylglycine

for (e) define number of repeats

Comments on the SI

The procedures are well described - please give a final proof read.

Examples of small points:

With %s, define if v/v, m/v....

Protein Expression & Purification

"The lysate was`' to "The lysates were"

Fig S1. Define MW markers. Were protein identities confirmed by MS analysis?

Supplementary Figure 2. Please define errors.

TabS assays

"An equal volume...."

Epimerase assays

CoA ligase assays

Assays with CoA ligases were set up containing 3 mM ATP (pH 8.0), 1.5 mM coenzyme A, 10 mM MgCl2, 4 mM phenylacetic acid (Paa) or phenoxyacetic acid (Poa)

Computational Modelling & Docking Studies

A few more details would be good - was this docking or molecular dynamics?

Supplementary Figure 9. Add error definition please.

Reviewer #4

(Remarks to the Author)
I found this to be a very well written and constructed manuscript that details the idea to use alternate enzymes to replicate the function of ACV synthase in the assembly of the classic peptide antibiotic penicillin. The work details how the authors identified and/or engineered enzymes for each of the necessary steps in this biosynthetic pathway (2x peptide bond formation steps, an epimerization step and the final conversion of the peptide into the active antibiotic structure). Each of these enzymes has good to moderate activity, and the authors also use some non-specific chemistry taking advantage of the presence of a cysteine residue within the peptide structure to generate one of the two peptide bonds.

Whilst I like the study and think it makes an novel contribution, I feel that the discussion of such a pathway as being the likely ancestral mechanism for penicillin biosynthesis to be dramatically overstated - such a pathway does not in any way explain how an ancestral ligase-driven pathway would evolve into a modern NRPS pathway for example. To my mind this seems to be replacing a difficult challenge with an even more difficult one!

The work also suffers from the major flaw that none of the steps are “linked/telescoped”, and are all performed in isolation. This means that the individual specificities for some of the processes can be “hidden” as it were, given that the authors control the entire reaction conditions for each enzymatic step. One of the most telling example in this case is the use of an engineered fatty acyl-CoA ligase to activate the N-terminal residue, and then adding the downstream dipeptide to intercept the activated intermediate by virtue of a nature chemical ligation type mechanism from the N-terminal cysteine residue. This reaction is entirely driven by the presence of an activated intermediate (i.e. thioester), which means that in a cellular environment this reaction would comprise a tiny minority of the likely products to be formed due to the high cellular concentration of acetyl-CoA (as one example). This is also a major limitation in the assertion that this pathway is a likely ancestral penicillin biosynthesis pathway, and in fact highlights why nature would use a “complex” NRPS for such a pathway - the requirement for enzymes to enforce specificity at each of the steps of the biosynthesis.

Thus, whilst I appreciate the work that has been performed and the ideas behind it, I do think the manuscript should include the combinations of multiple steps (1+2, 2+3) and also an entire “one-pot” reaction to truly assess how such a pathway compares to the NRPS machinery we see in ACV synthase. I also think that the discussion is very much a one-sided view of the data rather than an objective attempt to see how well such a pathway compares to the native pathway. With more complete data presented for the assembled ligase pathway (also with competing substrates to assess how such factors would influence this kind of pathway in vivo) this would mean a more nuanced discussion could be engaged in, where the impressive nature of what nature has achieved could be better highlighted.

Some more points to consider:

Figure 1 neglects the remnant half C-domain that is at the N-terminus of the NRPS (DOI: 10.1002/mbo3.1145)

Figure 3 - it is a touch misleading to show the structure of Hpg in the figure when this is unable to be activated by the engineered enzymes - perhaps show in grey to distinguish?

The paper drives very nicely through the different logical enzyme steps that need to be achieved to generate penicillin analogues using an engineered pathway; aspects that are missing include not testing the compatibility of these enzymes in a one pot assay to truly make these equivalent to an ACV synthase.

Discussion - TE domains can also epimerize residues; may not need a separate epimerize but this could be an optimization.

I think the idea of a ligase pathway shown here is interesting but can't be used in of itself to say that an early pathway for penicillin existed in such a manner - if so, this makes me ask how then did this become an NRPS pathway? That's arguably harder than evolving related enzymes (like those from lysine biosynthesis in fungi, see DOI: 10.1002/cbic.201500190, which share both the N-terminal A-domain as well as the remnant C-domain)

In terms of spontaneous NCL shift chemistry - with the balance of thioesters in the cell overwhelmingly in favor of molecules such as Ac-CoA this would make such a "non-guided" pathway difficult to see being in any way effective. This should be at least discussed and likely also tested to see the effects of competition on synthesis.

Finally, the overall conversion should be indicated for the individual steps, as well as how this is affected by combining steps once this has been performed.

Version 1:

Reviewer comments:

Reviewer #1

(Remarks to the Author)
The authors have been very responsive and made many improvements to the manuscript (most notably doing a 4 pot cascade reaction and a higher yielding 3 pot cascade as well as removing discussion of the evolution). Based on these revisions, this manuscript is now well suited for publication at Nat. Comm.

Reviewer #2

(Remarks to the Author)
I co-reviewed this manuscript with one of the reviewers who provided the listed reports. This is part of the Nature Communications initiative to facilitate training in peer review and to provide appropriate recognition for Early Career Researchers who co-review manuscripts.

Reviewer #3

(Remarks to the Author)
All 3 reviewers raised the same points for the authors to consider, most importantly to demonstrate the 'one-pot' viability of the approach. The authors have done this and whilst there is obvious scope for optimisation the procedure clearly works. Other minor points including removing evolutionary speculation have been addressed.

The only remaining minor point I have is that the authors and editors may consider a better title than "Alternative Enzymatic Pathways to Penicillin Antibiotics", which seems a bit vague. I know journals don't like novel / new but in this case it may be justified.

Anyway, congratulations on a very nice study!

Reviewer #4

(Remarks to the Author)
The authors have made a commendable effort in responding to the comments of the reviewers. The removal of the speculation regarding the evolution of penicillin biosynthesis is welcome, and investigating the efficacy of both 3-step and 4-step enzyme reactions brings balance to the discussion in terms of the challenges that still remain for such a pathway to be competitive with existing in vivo approaches; the one exception to this was that the discussion of the effect of the thioester (CoA) pool in vivo as mentioned in the response to the reviewers comments has not been noted in the manuscript - it should be. Characterization of the activities for each of enzymes involved in the cascade is also very important data that is now included.

Some specific points:

1. The introduction mentions "3-hydroxyphenylglycine (Hpg)" - Hpg usually refers to 4-hydroxyphenylglycine.

2 Regarding the challenge with TCEP, have the authors considered using TCEP resin (commercially available) as this would simplify removal without requiring extraction.

3. Is the overall yield reported from the concentrations of Cys/ Val? Clarification of this in the text would be helpful.

4. Discussion regarding the challenges with using native chemical ligation has not been included in the manuscript - I would suggest some form of the following text (taken from the authors response to reviewers) would be appropriate given that this would be a significant hurdle to overcome in moving the assay into an in vivo setting:

"...we agree with the reviewer that developing an in vivo cascade that relies on native chemical ligation (NCL) to couple CoA-thioesters with L-Cys-D-Val could be complicated due to competition with other thioesters (e.g. acetyl-CoA)."

Open Access This Peer Review File is licensed under a Creative Commons Attribution 4.0 International License, which permits use, sharing, adaptation, distribution and reproduction in any medium or format, as long as you give appropriate credit to the original author(s) and the source, provide a link to the Creative Commons license, and indicate if changes were made.
In cases where reviewers are anonymous, credit should be given to 'Anonymous Referee' and the source.
The images or other third party material in this Peer Review File are included in the article's Creative Commons license, unless indicated otherwise in a credit line to the material. If material is not included in the article's Creative Commons license and your intended use is not permitted by statutory regulation or exceeds the permitted use, you will need to obtain permission directly from the copyright holder.
To view a copy of this license, visit https://creativecommons.org/licenses/by/4.0/

Responses to Reviewers comments:

Reviewer #1 and #2: In this work, Micklefield and coworkers demonstrate a novel biocatalytic route to access penicillin and derivatives. Instead of using the native NRPS biosynthetic machinery, they chose to utilize easier to evolve stand alone ligases and epimerases, in combination with native IPNS cyclases. They utilized TabS to generate the L-Val/ L-Cys amide bond, taking advantages of its ability to utilize the unnatural L-SPro to avoid Val dimer formation and simplify purification. Epimerization of the L-Val to D-Val was accomplished using an epimerase from T. maritima, which was predicted to work based on docking studies with its crystal structure. N-acylation was cleverly install via utilization a Co-A ligase, which acylates the Cys, followed by an S- to N-acyl shift, similar to what occurs in NCL. While one of the ligases tested (PhlA) worked well for Paa and Poa (precursors to penicillin g and v) and even Phe to a small extent, it was unable to accommodate phenylglycine or hydroxyphenylglycine (precursors for ampicillin and amoxicillin). AlphaFold models combined with docking revealed several potential mutation sites, of which A338G and T369S enabled access to PgCV in moderated conversions. Unfortunately, no mutants had any activity towards hydroxyphenylglycine. After accessing the linear precursor, different IPNS enzymes were explored. It was found that an S185R mutant resulted in higher activities towards PaaCV and PoaCV. Ultimately, a mutant caIPNS was found to be the best for this conversion, in combination with a catalase buffer system to minimize ROS levels that are often observed with the Fe/ascorbate system. Further mutants of caIPNS S185R were explored based on AlphaFold3 models, with moderate improvements beings observed.

The **manuscript is generally well written** (a few grammar/typos) with a **strong Supplementary Section** supporting the main manuscript. The ability to biocatalytically produce penicillin and derivatives could be quite useful. However, it is unclear to me whether the described method is truly superior to the current semisynthetic method. As written, it appears that each intermediate is isolated before being subjected to the next enzyme in the cascade. It is also unclear whether this method is superior to simply chemically synthesizing the linear molecule followed by enzymatic cyclization by IPNS. If the authors could show a one-pot biocatalytic total synthesis using the enzymes that they discovered, this would greatly improve the paper and demonstrate improvements over the current methods.

Additionally, the discussion about the ligases potentially being ancient biosynthetic pathways for the penicillins seems a bit far-fetched/not supported. While it is possible that the original penicillins were made by ligases, there is no evidence (e.g. ancient DNA with an IPNS present but no NRPS) that supports this. Overall, despite these issues, **it is a strong paper that would be appropriate for publication in Nat. Commun. after major revisions**. Please see below for other smaller revisions that are required in addition to those described earlier in this paragraph.

We are grateful for the reviewer's helpful comments which we have fully addressed below.

Major Revisions:

[1.1] ability to do a one-pot total biocatalytic synthesis (or at least the ability to feed unpurified products into the next enzyme) are needed to demonstrate the true utility of this approach over other approaches (e.g. semisynthesis or linear synthesis followed by IPNS)

As suggested by the reviewer, we have carried out a cascade reaction comprising all four enzymes (TabS, epimerase, CoA ligase, and IPNS). The four-enzyme cascade reaction, starting from L-Val and L-Cys, produced penicillin G and penicillin V in modest yields of 3% and 6.5%, respectively, with ampicillin produced in trace quantities. The first three reactions of the four-enzyme cascade (TabS, epimerase and CoA-ligase) forming N-acyl-L-Cys-L-Val peptides are all carried out in the "one-pot." As IPNS is incompatible with TCEP, we need to remove TCEP by solvent extraction before adding IPNS to produce the final penicillin products, without any intermediate purification steps. We also developed a three-enzyme cascade reaction starting from the dipeptide L-Cys-L-Val which produced penicillin G, penicillin V, and ampicillin in improved yields of 18.2%, 22.7%, and 4%, respectively. **The cascade reactions are discussed in the paper p6, line 251-281, materials and methods (p6 of SI), and in additional supplementary Figure 14-15 (p31-32, SI).** We acknowledge that our enzymatic cascades to penicillin derivatives do not currently compete with the semi-synthetic methods used in industry. The industrial routes have been developed and optimised over more than 50 years. Nevertheless, we outline how our approach **could be improved through enzyme and reaction engineering (p6, line 275-281)**.

In the conclusion (see p7, line 303-306), we also note that there are very few examples where a new totally biocatalytic route have been developed to produce important synthetic therapeutic agents (as in our paper). A prominent example is Merck's enzymatic cascade to islatrivir (ref. 56). We have worked independently with the Merck's biocatalysis team, which comprises a very large group of experts with resources that few academic teams could match. Even with these capabilities, the final overall yield of the islatrivir cascade was only 51%. Furthermore, their paper (ref. 56) shows that each enzyme in the cascade underwent multiple rounds (2-12) of directed evolution to improve the initial conversions (some started at < 1%). While our team and resources cannot compete with Merck and Codexis in terms of high-throughput enzyme engineering, we believe that, should an industrial group wish to optimise our penicillin route, it could be further developed to achieve substantially improved performance. **We also highlight that the concept we introduced for using ligases for peptide synthesis may offer broader value for production of many other peptide-derived pharmaceuticals (see p7, line 307-316).**

[1.2] The discussion of the evolution seems far-fetched

There is evidence, which we cited in the original paper, that some ligase-based pathways (e.g. to glutathione and bacterial cell wall peptides) are more ancient than the NRPS pathways responsible for peptide natural products. Nevertheless, we agree that it is difficult to provide definitive experimental evidence to support the evolutionary ideas we put forward. In light of this, **we have removed the discussion on pathway evolution from the revised manuscript. The title and abstract have been slightly modified, and the conclusions section (p7, line 296-316) has been re-worked**. Although these evolutionary considerations initially inspired our approach, we are pleased that the reviewer concludes the paper is strong and suitable for *Nature Commun*, despite their reservations regarding the evolutionary discussion.

Minor Revisions:

[1.3] In figure 1a, "ACV" the molecule and "IPNS" the enzyme are in the same font/bolded – some distinction should be made

Figure 1a have been updated. The enzyme names are now in bold and product names are in standard text.

[1.4] Figure 1a, cephalosporin spelled wrong – spelled "cephalosprin C"

The spelling has been corrected in Figure 1a.

[1.5] Line 60, after "cyclisation by IPNS would generate ampicillin and amoxicillin" they should add ", respectively"

The grammatical error on page 2, line 60, has been corrected.

[1.6] Line 66, they say "ACVS" for the first time but do not define its definition – previously "ACV synthetase"

ACVS is now defined as '*L-δ-(α-aminoadipoyl)-L-cysteinyl- D-valine synthetase (ACVS)*' on P2, line 45.

[1.7] Line 65, change "group, combined with the necessity" to "group; this, combined with the necessity"

The grammatical error in line 65 has been corrected.

[1.8] Line 88, "cyclised by IPNS variant" to "cyclised by an IPNS variant"

The correction has been included in line 87 (*previously line 88*).

[1.9] Line 97, remove either "involving" or "using"

The word 'involving' is removed from line 93 (*previously line 97*).

[1.10] Val dimerization is observed. Is cysteine dimerization every seen with the TabS? Just curious

TabS does not produce the other two combinations of L-Cys and L-Val, i.e. L-Cys-L-Cys and L-Val-L-Cys, as L-Cys is not preferred as the C-terminal (nucleophilic) substrate for TabS.

[1.11] In general, some of the grammar needs correcting.

We have proofread the revised manuscript very carefully, and where there are any additional grammatical errors, we have fixed them.

[1.12] Line 118, claim that L-Val dimer is 4%. That said, Figure 2C, it looks like there is more than 4% of the dimer. Is the graph correct?

We have rechecked the calculation and it was observed that the L-Val-L-Val dipeptide was formed in 9% yield in the TabS reaction carried out with 2:1 L-SPro and L-Val. The information has been corrected (p3, line 116). However, we would like to note that the L-Val-L-Val displays higher ionization (protonation) efficiency under ESI-MS conditions, resulting in a higher peak area even at lower concentrations compared to L-Cys-L-Val or L-SPro-L-Val. The Cys-Val or SPro-Val dipeptide has less protonation efficiency likely due to the presence of the thiol functionality, leading to lower observed MS signal intensities at comparable concentrations.

[1.13] Line 191 – Any thoughts on why this did not work w D-Hpg? More explanation is needed

We have provided some explanation why the PhlA mutants did not work with D-Hpg (p5, line 189-193). In PhlA active site, the aromatic ring of the Paa-AMP intermediate is predominantly surrounded by hydrophobic residues (**Figure 3d**), which may not favour binding of the more polar phenol side chain of D-Hpg. Mutations of PhlA substrate-binding pocket, have so far been insufficient to establish productive interactions with this substrate. Further engineering of PhlA is likely to be required accommodate the molar polar D-Hpg side chain.

[1.14] The mutagenesis studies should be summarized in a table rather than shown in the bar graph. That would make it easier to determine the differences

The activities of the PhlA mutant variants are now presented in Supplementary Table 1 on page 19 of Supporting Information.

[1.15] Figure 2, there is a box around TabS that should be removed

The box around TabS structure in Figure 2 has been removed.

[1.16] Line 122 – need to define methoxyamine hydrochloride acronym for figure 2a

The acronym has been described in line 121 as follows; 'Incubation of L-SPro-L-Val with methoxyamine hydrochloride ($MeONH_2$.HCl) and TCEP, afforded L-Cys-L-Val in >95% yield.'

[1.17] Need to be clearer/more consistent with labeling of compounds/acronyms

We identified some possible labelling issues and have fixed these.

[1.18] Figure 3e – duplicate only? Why not triplicate; also, if only duplication, I do not think it is correct to show error bars

The triplicate data set has been included in Figure 3e.

[1.19] Figure 3f – a "no enzyme" control should be shown

LC-MS trace for the no enzyme control is included in Figure 3f.

[1.20] Figure 4c – needs to be better quality/a little bigger (or shown as a table rather than a bar graph)

We have replaced the bar chart, in Figure 4c with a table, as suggested.

[1.21] In general, the organization of the SI could be improved. It would be better to have all of the supplemental methods together then the SI Figures then the SI tables.

We have revised the SI, and all supplemental methods are presented together at the beginning, followed by the SI Tables and SI Figures and NMR spectra at the end of the SI.

[1.22] SI Cloning and Mutagenesis section, what antibiotic and what concentration was used for protein expressions?

The antibiotic details and concentration used for protein purification are described on p3 of the Supporting Information.

[1.23] Need to give sequences of primers used

We have provided a table including the sequence of the primers used (p18 of SI).

[1.24] SI fig 9 – table form would be better or a good addition- SI fig 10 – need to a), b) , c) to figure legend. Also, how many replications for SI figure 10 panel C?

The data represented in Supplementary figure 9 are now represented in Supplementary table 1 (p19 of SI). The figure numberings are now included in the revised Supplementary Figure 9 (previously Supplementary Figure 10). The panel c, of the new Supplementary figure 9, represents data from three independent replicates and further details are included in the figure legend.

[1.25] Add delta ppms for all mass specs

Δ*ppm* calculations for the mass spec analysis are included in the Supplementary information (please see p22, 24-25, and 28).

[1.26] SI Figure 17, C13 for the PaaCV dimer does not have good enough S/N

An improved ^{13}C NMR spectra for the PaaCV is now included as Supplementary Figure 18 (*previously SI Figure 17)*.

[1.27] SI Figure 21, no 13C? Also the annotation for this 1H NMR is different from others (do not outline which proton goes with what)

The ^{13}C NMR of D-PgCV isolated from CoA ligase assay is now included in Supplementary figure 22. The annotations (atom numbering) have been removed from all the NMR spectra as suggested.

Reviewer #2: I co-reviewed this manuscript with one of the reviewers who provided the listed reports. This is part of the Nature Communications initiative to facilitate training in peer review and to provide appropriate recognition for Early Career Researchers who co-review manuscripts.

We are grateful to this co-reviewer for contributing to the process.

Reviewer #3: **I like the concept underlying this paper**, i.e. to 'replace; natural occurring non ribosomal peptide synthetases with engineered small peptide synthetases to make precursor peptides for cyclising to medicines, in this exemplar case penicillins.

The sequence is promoted as being a potentially useful alternative to the established semi-synthesis route to penicillins. At the concept stage commercial viability is not a prime concern, however, The cost of producing even one, let alone multiple recombinant proteins. is surely going to be greater than that of a fermented penicillin. The current procedure is in fact highly efficient and penicillin availability is principally limited by regulations narrowing its production to specialized facilities.

[3.1] Nonetheless, to repeat **I really do like the concept**, but there are several key points that need clarifying. Firstly as I understand from the manuscript, all the individual enzyme reactions were optimized to produce products that could be used by the next enzyme in the sequence. It was not clear to me however, whether the products that were produced in one enzyme catalysed step were passed on to the next enzyme or whether synthetic materials were used. What is the overall efficiency of the entire enzyme catalyzed procedure?

Further can the entire enzyme catalysed procedure be used in the same pot? If not what is the most efficient procedure? At least some attempts to optimise the overall procedure should be presented. The authors did manage to combine the epimerase and PhlA enzymes productively, so must have tried other combinations?

The individual enzyme reactions were first optimized with synthetic substrates, and the enzymatic products were isolated and subjected to NMR for structure determination. The isolated enzymatic products can be taken forward into the next enzymatic reaction sequentially. In addition, as suggested by the reviewer, we have carried out a cascade reaction comprising all four enzymes (TabS, epimerase, CoA ligase, and IPNS). The four-enzyme cascade reaction, starting from L-Val and L-Cys, produced penicillin G and penicillin V in modest yields of 3% and 6.5%, respectively, with ampicillin produced in trace quantities. The first three reactions of the four-enzyme cascade (TabS, epimerase and CoA-ligase) forming N-acyl-L-Cys-L-Val peptides are all carried out in the “same pot.” As the IPNS enzyme is incompatible with TCEP, we needed to remove TCEP by solvent extraction before adding IPNS to produce the final penicillin products, without any intermediate purification steps. We also developed a three-enzyme cascade reaction starting from the dipeptide L-Cys-L-Val which produced penicillin G, penicillin V, and ampicillin in improved yields of 18.2%, 22.7%, and 4%, respectively. **The cascade reactions are discussed in the paper p6, line 251-281, materials and methods (p6 of SI), and in an additional supplementary Figure 14-15 (p31-32, SI).**

We agree, with this reviewer, that it is difficult to compete with commercial routes to semi-synthetic penicillin derivatives which have been developed over more 50 years. We are providing a proof-of-concept, for a completely different approach, and not an industrially optimised process. Nevertheless, we outline how our approach **could be improved through**

enzyme and reaction engineering (p6, line 275-281). We also highlight that the concept we introduced, using ligases for peptide synthesis, **may offer broader value to produce other peptide-derived pharmaceuticals (see p7, line 307-316).**

[3.2] Ideally of course the entire process would be engineered into cells - was this considered or is it the subject of ongoing work. It would seem likely this is the only way that the new procedure could possibly compete with the existing highly efficient procedure.

We agree that implementing the full pathway in a cellular system would be an important direction for future research. However, the focus of the present study was the development and engineering of the necessary enzyme activities to produce the penicillin derivatives *in vitro*. We do believe it may be possible to establish an alternative pathway to the penicillin antibiotics *in vivo*, but significant additional enzyme engineering and metabolic engineering of a suitable host strain would be required, which would be beyond the scope (time-scale) of this current paper.

[3.3] There is also very little information on the kinetics of the individual enzyme catalyzed reactions. Information on what scale the entire enzyme catalysed procedure can be carried out on would be useful.

We have determined the specific activity of each enzyme (TabS, epimerase, PhlA, and IPNS) and some of the mutants used in the study. **The new data on the specific activity of the enzymes is discussed in the paper p6, line 266-268, materials and methods (p6 of SI), and in an additional supplementary Table 2 (p20, SI).** The scale/volume of all of the enzyme catalysed are also included in the materials and methods section of Supplementary Information (p4-7).

[3.4] Our final point is whether or not the work really relates to evolution - it's attractive to propose that small molecule synthetases evolved before the more complex non ribosomal peptide synthetases, but I'm not sure there's any actual evidence for this, at least for those studied (glutathione is not in all bacteria?). My own feeling is the work is better presented as a novel sequence of reactions leading to the precursor tripeptide for penicillin formation, rather than as an ancestral / evolution type story.

Following the reviewers' suggestions, **we have removed the discussion around the evolutionary origins of ligase pathways from the paper. We have modified the title and abstract, and we have reworked the conclusion (p7, lines 287–316)** emphasizing our broader approach for developing novel enzymatic routes to peptide derived pharmaceuticals. The enzymes described in our paper are simpler, more versatile, and potentially easier to engineer than NRPSs and other complex biosynthetic machinery responsible for producing most peptides in nature. Beyond penicillin antibiotics, our approach could provide alternative routes for the synthesis of other valuable peptide therapeutics (also see ref 57-58).

[3.5] Overall I'm keen on the work but the issues above need to be addressed in particular with respect to whether or not the entire sequence can be catalyzed in an enzyme catalyzed way and whether some or all the individual reactions can be combined.

We are pleased that the reviewer is keen on the work. As stated above (3.1), **we have introduced cascade reactions into the paper (p6, line 251-281), materials and methods (p6 of SI), and in additional supplementary Figure 14-15 (p31-32, SI).** We appreciate that the yields of the cascades are low. However, we have not carried out extensive reaction or enzyme engineering. **We explain potential bottlenecks in the cascades and suggest ways in which the cascades could be improved (p6, line 275-281)**.

In the conclusion (see p7, line 303-306), we also note that there are very few examples in where a new totally biocatalytic route have been developed to produce important synthetic therapeutic agents (as in our paper). A prominent example is Merck's enzymatic cascade to islatrivir (ref. 56). We have worked independently with the Merck's biocatalysis team,

which comprises a very large group of experts with resources that few academic teams could match. Even with these capabilities, the final overall yield of the islatrivir cascade was only 51%. Furthermore, their paper (ref. 56) shows that each enzyme in the cascade underwent multiple rounds (2-12) of directed evolution to improve the initial conversions (some started at < 1%). While our team and resources cannot compete with Merck and Codexis in terms of high-throughput enzyme engineering, we believe that, should an industrial group wish to optimise our penicillin route, it could be further developed to achieve substantially improved performance.

[3.6] Please think about the title - is the use of ancestral really justified? What is the evidence? Maybe" "A novel enzymatic pathway to penicillins" or some variation on this. The abstract is fine - it does not overclaim.

We have changed the title to "Alternative enzymatic pathways to penicillin antibiotics." We agree this work is novel, but the journal may prefer we avoid using this word in the title.

[3.7] Introduction: L47 IPNS is not a synthetase as far as I know - ATP not involved?

The reviewer is correct, ATP is not required by IPNS, and we have changed line 47 to include synthase.

[3.8] L50 delete "laborious" - the route is super-efficient - penicillins are

cheap. The laborious word has been deleted.

[3.9] Results: please comment the efficiency of on the ligase, epimerase, cylation reactions- at least in terms of specific activities and in selected cases by kcat / Km values.

Measuring k_{cat}/K_m is feasible for some of the enzymes studied; however, reliable kinetic characterisation is not straightforward for enzyme TabS. The TabS enzyme generates two reaction products, and kinetic measurements based on the inorganic phosphate assay do not accurately reflect the rate of L-Cys-L-Val formation, thereby the meaningful calculations for k_{cat}/K_m values for this product cannot be accurately determined.

As a result, direct comparison of k_{cat}/K_m values across the full set of enzymes and mutants would be problematic. We therefore report **specific activities for the four enzymes and selected mutants in Supplementary Table 3.** The specific activity measurements were determined using a standard approach for all enzymes, allowing for a more consistent and comprehensive comparison of catalytic performance. Based on these data, together with other considerations, we conclude that TabS and IPNS activities are lowest, and these enzymes would need further engineering to improve the cascade reactions.

[3.10] Line 141 "therefore proceeded to the next step with the L-Cys-D-Val dipeptide"

We corrected this sentence to read more clearly (line 140).

[3.11] Cyclisation of peptides into β-lactam antibiotics; Line 196 IPNS is an oxidase not an oxygenase as no O_2 derived O atoms in its product? Please correct.

This has been corrected.

[3.12] Please comment on the catalytic efficiency of the mutants - at least in terms of specific activities and in selected cases by kcat / Km values.

As mentioned above (3.3 & 3.9), specific activities for the four enzymes and selected mutants have been included in Supplementary Table 3.

[3.13] Discussion: Perhaps a bit of clarification useful: These antibiotics likely arose through convergent evolution with respect to the b-lactam ring forming reactions. ??
Please check are all beta-lactam formed from peptides - what about clavams?

The discussion about evolution of penicillin pathways has been removed, so this sentence is no longer present.

[3.14] Figure 2. "the ʟ-Val-ʟ-Val side product."

This grammatical error has been corrected in the figure caption.

[3.15] (d) Assay showing the epimerisation. Figure 3e/4c, D-phenylglycine/D-hydroxyphenylglycine, for (e) define number of repeats

The number of experimental repeats for Figure 3e and 4c has been described in figure captions in the paper (p10-11).

[3.16] Comments on the SI: The procedures are well described - please give a final proofread.

The Supplementary Information has been carefully checked, and any grammatical errors have been corrected to improve clarity and consistency.

[3.17] Examples of small points: With %s, define if v/v, m/v....

These have been included in the Supporting information (p2-7).

[3.18] Protein Expression & Purification: "The lysate was`' to "The lysates were"

This grammatical error has been corrected in Supporting information.

[3.19] Fig S1. Define MW markers. Were protein identities confirmed by MS analysis?

The MW markers for Western blot is now defined in the caption of the Supplementary Figure 1. The molecular weight of the purified proteins was determined using the Western blot and has not been confirmed by MS analysis.

[3.20] Supplementary Figure 2. Please define errors.

These have been included in the Supplementary Information figure caption.

[3.21] TabS assays: "An equal volume...."

This grammatical error has been corrected.

[3.22] Epimerase assays, CoA ligase assays; Assays with CoA ligases were set up containing 3 mM ATP (pH 8.0), 1.5 mM coenzyme A, 10 mM MgCl2, 4 mM phenylacetic acid (Paa) or phenoxyacetic acid (Poa)

We are not entirely sure what the reviewer is asking us to change here. However, experimental details for the assay have been edited to improve clarity.

[3.23] Computational Modelling & Docking Studies: A few more details would be good - was this docking or molecular dynamics?

The modelling was done using only molecular docking techniques, no molecular dynamics simulation was used in this study. More details have been added to the computational modelling section in the supplementary information (p3).

[3.24] Supplementary Figure 9. Add error definition please.

The error definition has been included in the caption of Supplementary Figure 9

Reviewer #4 I found this to be a **very well written and constructed manuscript** that details the idea to use alternate enzymes to replicate the function of ACV synthase in the assembly of the classic peptide antibiotic penicillin. The work details how the authors identified and/or engineered enzymes for each of the necessary steps in this biosynthetic pathway (2x peptide bond formation steps, an epimerization step and the final conversion of the peptide into the active antibiotic structure). Each of these enzymes has good to moderate activity, and the authors also use some non-specific chemistry taking advantage of the presence of a cysteine residue within the peptide structure to generate one of the two peptide bonds.

[4.1] Whilst **I like the study and think it makes an novel contribution**, I feel that the discussion of such a pathway as being the likely ancestral mechanism for penicillin biosynthesis to be dramatically overstated - such a pathway does not in any way explain how an ancestral ligase-driven pathway would evolve into a modern NRPS pathway for example. To my mind this seems to be replacing a difficult challenge with an even more difficult one!

Following the reviewers' suggestions, **we have removed the discussion around the ancestral ligase pathways from the paper. We have modified the title and abstract, and we have reworked the conclusion (p7, lines 287–316)** emphasizing our broader approach for developing novel enzymatic routes to peptide derived pharmaceuticals. The enzymes described in our paper are simpler, more versatile, and potentially easier to engineer than NRPSs and other complex biosynthetic machinery responsible for producing most peptides in nature. Beyond penicillin antibiotics, our approach could provide alternative routes for the synthesis of other valuable peptide therapeutics (also see ref 57-58).

[4.2] The work also suffers from the major flaw that none of the steps are "linked/telescoped", and are all performed in isolation. This means that the individual specificities for some of the processes can be "hidden" as it were, given that the authors control the entire reaction conditions for each enzymatic step. One of the most telling example in this case is the use of an engineered fatty acyl-CoA ligase to activate the N-terminal residue, and then adding the downstream dipeptide to intercept the activated intermediate by virtue of a nature chemical ligation type mechanism from the N-terminal cysteine residue. This reaction is entirely driven by the presence of an activated intermediate (i.e. thioester), which means that in a cellular environment this reaction would comprise a tiny minority of the likely products to be formed due to the high cellular concentration of acetyl-CoA (as one example). This is also a major limitation in the assertion that this pathway is a likely ancestral penicillin biosynthesis pathway, and in fact highlights why nature would use a "complex" NRPS for such a pathway - the requirement for enzymes to enforce specificity at each of the steps of the biosynthesis.

Thus, whilst I appreciate the work that has been performed and the ideas behind it, I do think the manuscript should include the combinations of multiple steps (1+2, 2+3) and also an entire "one-pot" reaction to truly assess how such a pathway compares to the NRPS machinery we see in ACV synthase. I also think that the discussion is very much a one-sided view of the data rather than an objective attempt to see how well such a pathway compares to the native pathway. With more complete data presented for the assembled ligase pathway (also with competing substrates to assess how such factors would influence this kind of pathway in vivo) this would mean a more nuanced discussion could be engaged in, where the impressive nature of what nature has achieved could be better highlighted.

We have carried out *in vitro* cascade reactions comprising all four enzymes (TabS, epimerase, CoA ligase, and IPNS). The four-enzyme cascade reaction, starting from L-Val and L -Cys, produced penicillin G and penicillin V in modest yields of 3% and 6.5%, respectively, with ampicillin produced in trace quantities. We also developed a three-enzyme cascade reaction starting from the dipeptide L-Cys-L-Val, which produced penicillin G, penicillin V, and ampicillin in improved yields of 18.2%, 22.7%, and 4%, respectively. **The cascade reactions are discussed in the paper p6, line 251-281, materials and methods (p6 of SI), and in additional supplementary Figure 14-15 (p31-32, SI).** The first three reactions of the four-enzyme cascade (TabS, epimerase and CoA-ligase) forming N-acyl-L-Cys-D-Val peptides are all carried out in "one-pot." Because IPNS is incompatible with TCEP, we needed to remove TCEP by solvent extraction before adding IPNS to produce the final penicillin products. If this cascade was running in the reducing environment of the cell (i.e. *in vivo),* this would not be an issue. However, we agree with the reviewer that developing an *in vivo* cascade that relies on native chemical ligation (NCL) to couple CoA-thioesters with L-Cys-D-Val could be complicated due to competition with other thioesters (e.g. acetyl-CoA).

Although it would be challenging and beyond the timescale of the review process, it may be possible to develop an *in vivo* cascade. It is likely that we would require a ligase that catalyses both activation and amide bond formation, rather than NCL, to generate N-acyl-L-Cys-D-Val

intermediates. We have discovered several new ATP-ligases from other natural product pathways (unpublished), which with engineering may be candidates for N-acylation of L-Cys-D-Val. Also in peptidoglycan biosynthesis, some of the stand-alone pentapeptide forming ligases are suggested to be involved in larger multiprotein complexes, which may facilitate proximity channelling [Pareek *et al*]. Engineering protein clustering to control the diffusion of intermediates (e.g. thioesters) is therefore another potential approach to improve the selectivity and efficiency of *in vivo* cascades [Castellana *et al.* & Wheeldon *et al.*].

We also believe that it should be possible to improve the *in vitro* cascades. While the yields of the *in vitro* cascades we describe in the paper are low, we have carried out minimal enzyme engineering. **We have discussed some of the challenges (bottlenecks) we have encountered in developing the *in vitro* cascades, and suggested ways to improve the cascades (p6, line 275-281).**

In the conclusion (see p7, line 303-306), we also note that there are very few examples in where a new totally biocatalytic route have been developed to produce important synthetic therapeutic agents (as in our paper). A prominent example is Merck's enzymatic cascade to islatrivir (ref. 56). We have worked independently with the Merck's biocatalysis team, which comprises a very large group of experts with resources that few academic teams could match. Even with these capabilities, the final overall yield of the islatrivir cascade was only 51%. Furthermore, their paper (ref. 56) shows that each enzyme in the cascade underwent multiple rounds (2-12) of directed evolution to improve the initial conversions (some started at < 1%). While our team and resources cannot compete with Merck and Codexis in terms of high-throughput enzyme engineering, we believe that, should an industrial group wish to optimise our penicillin route, it could be further developed to achieve substantially improved performance. **We also highlight that the concept we introduced for using ligases for peptide synthesis may offer broader value, for the production of other peptide-derived pharmaceuticals (see p7, line 307-316).**

[4.3] Figure 1 neglects the remnant half C-domain that is at the N-terminus of the NRPS (DOI: 10.1002/mbo3.1145)

The remaining half of the C-domain of the ACVS NRPS is now depicted in the schematic diagram (Figure 1a).

[4.4] Figure 3 - it is a touch misleading to show the structure of Hpg in the figure when this is unable to be activated by the engineered enzymes - perhaps show in grey to distinguish?

As suggested, the structure of D-Hpg in Figure 3a is now represented in light grey to distinguish it from other substrates that can be activated by the PhlA enzymes.

[4.5] The paper drives very nicely through the different logical enzyme steps that need to be achieved to generate penicillin analogues using an engineered pathway; aspects that are missing include not testing the compatibility of these enzymes in a one pot assay to truly make these equivalent to an ACV synthase.

We have performed three- and four-enzyme cascades as described above (see 4.2).

[4.6] Discussion - TE domains can also epimerize residues; may not need a separate epimerize but this could be an optimization.

We agree with the referee that some TE domains can catalyse epimerisation, and we think they are suggesting using a TE domain as a standalone biocatalyst, rather than the epimerase from the thermophilic bacteria we use in our approach. Using a TE is a good idea, that we have considered, however there are some complications that may make this difficult to implement in our pathway. For example, the TE domain of ACVS can epimerise the C-terminal L-Val residue of L-aminoadipate-L-Cys-L-Val (ACV). This TE domain might therefore be used to epimerise the C-terminal residue of alternative N-acyl-L-Cys-L-Val peptides. However, given that native TEs recognise peptidyl-S-PCP thioester substrates, if we were to use a stand-alone TE, we would first need to activate and thiolate the C-terminal carboxyl group of N-acyl-L-Cys-L-Val to generate

C-terminal thioester (e.g. PCP, SNAC or CoA thioester). Also given that L-aminoadipate, is quite different in structure/polarity to Paa, Poa, Pg or Hpg, it is possible that the TE may not accept the non-native peptidyl-thioesters and would need engineering. An additional enzyme activity would also be required to activate and thiolate the C-terminus of the N-acyl-L-Cys-L-Val peptide intermediates. Although this might be achievable, it would be significantly more challenging than using the epimerase we describe in the paper.

[4.7] I think the idea of a ligase pathway shown here is interesting but can't be used in of itself to say that an early pathway for penicillin existed in such a manner - if so, this makes me ask how then did this become an NRPS pathway? That's arguably harder than evolving related enzymes (like those from lysine biosynthesis in fungi, see DOI: 10.1002/cbic.201500190, which share both the N-terminal A-domain as well as the remnant C-domain)

Given the reviewers are not entirely convinced by the arguments around the evolution of pathways to peptide precursors for penicillin biosynthesis, we have removed this discussion form the paper (see response to 4.1 above).

[4.8] In terms of spontaneous NCL shift chemistry - with the balance of thioesters in the cell overwhelmingly in favor of molecules such as Ac-CoA this would make such a "non-guided" pathway difficult to see being in any way effective. This should be at least discussed and likely also tested to see the effects of competition on synthesis.

We agree that cellular thioesters (e.g. Ac-CoA) could complicate implementation of *in vivo* cascades that rely on NCL. We have discussed this above (see 4.2). We have focussed on developing *in vitro* rather that *in vivo* cascades.

[4.9] Finally, the overall conversion should be indicated for the individual steps, as well as how this is affected by combining steps once this has been performed.

We have included yields for all enzymatic reactions including the cascades.

Point-by-point response to reviewer's comments:

Reviewer #1 and #2: The authors have been very responsive and made many improvements to the manuscript (most notably doing a 4 pot cascade reaction and a higher yielding 3 pot cascade as well as removing discussion of the evolution). Based on these revisions, this manuscript is now well suited for publication at Nat. Comm.

We thank the reviewers for the positive evaluation of our revised manuscript and for recognizing the improvements made during the revision process. We are pleased that the reviewers now consider the manuscript suitable for publication in Nature Communications.

Reviewer #3: All 3 reviewers raised the same points for the authors to consider, most importantly to demonstrate the 'one-pot' viability of the approach. The authors have done this and whilst there is obvious scope for optimisation the procedure clearly works. Other minor points including removing evolutionary speculation have been addressed.

The only remaining minor point I have is that the authors and editors may consider a better title than "Alternative Enzymatic Pathways to Penicillin Antibiotics", which seems a bit vague. I know journals don't like novel / new but in this case it may be justified.

Anyway, congratulations on a very nice study!

We thank the reviewer for the positive and encouraging comments on our work. We also appreciate the suggestion to consider refining the manuscript title. After careful consideration, we have decided to retain the current title, as we feel that its broader wording appropriately reflects the scope of the study and may appeal to a wide readership with interests in biosynthesis and biocatalysis. Nevertheless, we would be happy to consider any suggestions from the editors regarding the final title.

Reviewer #4: The authors have made a commendable effort in responding to the comments of the reviewers. The removal of the speculation regarding the evolution of penicillin biosynthesis is welcome, and investigating the efficacy of both 3-step and 4-step enzyme reactions brings balance to the discussion in terms of the challenges that still remain for such a pathway to be competitive with existing in vivo approaches; the one exception to this was that the discussion of the effect of the thioester (CoA) pool in vivo as mentioned in the response to the reviewers comments has not been noted in the manuscript - it should be. Characterization of the activities for each of enzymes involved in the cascade is also very important data that is now included.

4.1 The introduction mentions "3-hydroxyphenylglycine (Hpg)" - Hpg usually refers to 4-hydroxyphenylglycine.

As suggested, this has been corrected as 'an NRPS that incorporates phenylglycine (Pg) or 4-hydroxyphenylglycine (Hpg) may provide tripeptides.....' (Line 59, Page 2)

4.2 Regarding the challenge with TCEP, have the authors considered using TCEP resin (commercially available) as this would simplify removal without requiring extraction.

We thank the reviewer for this excellent suggestion. The use of TCEP immobilized on resin could, in principle, be advantageous for the cascade reaction as it may eliminate the need for an additional extraction step. However, in the present system we observed that the activity of IPNS is compromised not only by the presence of TCEP but also by other

components from the preceding enzymatic step. To investigate this, we performed control IPNS assays in the presence of individual components from the upstream CoA ligase reaction. These experiments showed that IPNS activity is inhibited both in the presence of TCEP and in the presence of the PhlA enzyme. Therefore, an extraction step is necessary in this case to remove these components before initiating the IPNS reaction.

4.3 Is the overall yield reported from the concentrations of Cyst Val? Clarification of this in the text would be helpful.

This has been clarified in the caption of Figure 5 in the main article.

4.4 Discussion regarding the challenges with using native chemical ligation has not been included in the manuscript - I would suggest some form of the following text (taken from the authors response to reviewers) would be appropriate given that this would be a significant hurdle to overcome in moving the assay into an in vivo setting:

“...we agree with the reviewer that developing an in vivo cascade that relies on native chemical ligation (NCL) to couple CoA-thioesters with L-Cys-D-Val could be complicated due to competition with other thioesters (e.g. acetyl-CoA).”

We appreciate the reviewer’s suggestion to include in the manuscript the discussion regarding the potential influence of the intracellular thioester (CoA) pool that was mentioned in our previous response to the reviewers. In response, we have now added a short discussion of this point to the manuscript (page 6, line 279-282).

‘Extension to an in vivo cascade may be feasible in the future. However, the use of NCL could be complicated due to endogenous thioesters (e.g., acetyl-CoA) and may require a different ligase enzyme for N-acylation of the L-Cys-D-Val precursor.’
